# Chemotherapeutic Activity of Pitavastatin in Vincristine Resistant B-Cell Acute Lymphoblastic Leukemia

**DOI:** 10.3390/cancers15030707

**Published:** 2023-01-24

**Authors:** Debbie Piktel, Javohn C. Moore, Sloan Nesbit, Samuel A. Sprowls, Michael D. Craig, Stephanie L. Rellick, Rajesh R. Nair, Ethan Meadows, John M. Hollander, Werner J. Geldenhuys, Karen H. Martin, Laura F. Gibson

**Affiliations:** 1 West Virginia University Cancer Institute, Robert C. Byrd Health Sciences Center, West Virginia University, Morgantown, WV 26506, USA; 2 Department of Microbiology, Immunology and Cell Biology, West Virginia University School of Medicine, Morgantown, WV 26506, USA; 3 Department of Pharmaceutical Sciences, West Virginia University School of Pharmacy, Morgantown, WV 26506, USA; 4 Departments of Cardiovascular and Metabolic Sciences, Lerner Research Institute, Cleveland Clinic, Cleveland, OH 44195, USA; 5 Case Comprehensive Cancer Center, Cleveland, OH 44195, USA; 6 Queen’s Health System, Honolulu, HI 96813, USA; 7 Department of Physiology and Pharmacology, School of Medicine, West Virginia University, Morgantown, WV 26506, USA; 8 Mitochondria, Metabolism & Bioenergetics Working Group, West Virginia University, Morgantown, WV 26506, USA; 9 Department of Neuroscience, School of Medicine, West Virginia University, Morgantown, WV 26506, USA

**Keywords:** sirtuins, statins, residual disease, bone marrow

## Abstract

**Simple Summary:**

Chemoresistance leads to poor prognostic outcomes in leukemic patients. Investigating the effects of pitavastatin in chemo-resistant ALL cells provides support for repurposing this FDA-approved drug to contribute to novel interventions to eradicate residual tumor cells that thrive in the protective niche of the bone marrow microenvironment.

**Abstract:**

B-cell acute lymphoblastic leukemia (ALL) is derived from an accumulation of malignant, immature B cells in the bone marrow and blood. Relapse due, in part, to the emergence of tumor cells that are resistant to front line standard chemotherapy is associated with poor patient outcomes. This challenge highlights the need for new treatment strategies to eliminate residual chemoresistant tumor cells. Based on the use of pitavastatin in acute myeloid leukemia (AML), we evaluated its efficacy in an REH ALL cell line derived to be resistant to vincristine. We found that pitavastatin inhibited the proliferation of both parental and vincristine-resistant REH tumor cells at an IC_50_ of 449 nM and 217 nM, respectively. Mitochondrial bioenergetic assays demonstrated that neither vincristine resistance nor pitavastatin treatment affected cellular oxidative phosphorylation, beta-oxidation, or glycolytic metabolism in ALL cells. In a co-culture model of ALL cells with bone marrow stromal cells, pitavastatin significantly decreased cell viability more robustly in the vincristine-resistant ALL cells compared with their parental controls. Subsequently, NSG mice were used to develop an in vivo model of B-cell ALL using both parental and vincristine-resistant ALL cells. Pitavastatin (10 mg/kg i.p.) significantly reduced the number of human CD45+ REH ALL cells in the bone marrow of mice after 4 weeks of treatment. Mechanistic studies showed that pitavastatin treatment in the vincristine-resistant cells led to apoptosis, with increased levels of cleaved PARP and protein-signaling changes for AMP-activated protein kinase/FoxO3a/Puma. Our data suggest the possible repurposing of pitavastatin as a chemotherapeutic agent in a model of vincristine-resistant B-cell ALL.

## 1. Introduction

B-cell acute lymphoblastic leukemia (ALL) is a neoplasm of precursor B cells, or B lymphoblasts. Although primarily a childhood disease, the prognosis is poor in both pediatric and adult patients who relapse. For adult patients who relapse due to chemoresistance, survival is poor, with an overall survival of 3–6 months [1]. The bone marrow microenvironment has been shown to be a unique sanctuary niche for leukemic cells, providing protective signaling to circumvent chemotherapy-induced death [1,2,3,4]. New approaches are necessary to eradicate hematologic malignancies residing in the bone marrow to blunt the relapse of aggressive disease. Targeting disease with conventional chemotherapy requires drug concentrations that are not well tolerated and could ultimately lead to chemoresistance and relapse due to the presence of minimal residual disease (MRD) [5]. Several studies have shown that metabolic changes in cancer cells, including both the mitochondria and lipids, can contribute to chemoresistance in multiple cancer types [6,7,8].

The repurposing of FDA-approved drugs is an important strategy to resolve clinical issues related to tolerance or side effects, with the overall goal of improving patient outcomes [9]. The identification of compounds capable of altering cancer metabolism may provide new avenues for manipulating the specialized metabolic changes found in hematologic malignancies, specifically targeting the leukemic cells that contribute to MRD.

The statin drug class has been shown to be a novel group of drugs with pharmacological benefits in multiple solid tumors [10]. The mechanism by which the statin drug, pitavastatin, elicits its anticancer activity has been investigated in solid tumors [11,12]; in contrast, pitavastatin has not been as extensively evaluated in hematologic malignancies, although the use of statin drugs has been investigated in chronic myeloid leukemia (CML) [13,14]. Recently, a phase I clinical trial was established for the use of pitavastatin in acute myeloid leukemia (AML), but no data have been published on the use of pitavastatin in B-cell ALL. In this study, we investigated the effect of pitavastatin on a vincristine-resistant B-cell ALL leukemia cell line to determine its potential utility as a novel treatment for chemoresistant MRD in the lymphoid setting.

## 2. Materials and Methods

All chemicals and cell culture reagents were obtained from commercial sources. Pitavastatin was obtained from Tocris and dissolved in dimethyl sulfoxide (DMSO), and the latter was used as vehicle control for cellular studies where noted.

### 2.1. Cell Lines

The cell line used in this study includes the leukemia REH cells (ATCC, Manassas, VA, #CRL-8286), which were grown using RPMI 1640 cell culture media with additional 10% fetal bovine serum, and the antibiotics penicillin/streptomycin and 0.05 mM 2-mercaptoethanol. A chemotherapy-resistant REH ALL cell line (REH-VR) was derived by exposing REH cells to increasing concentrations of vincristine. To select for vincristine-resistant cells, vincristine was added to the culture and cell viability was evaluated after 1 week. If cell viability was determined to be greater than 70%, the concentration of vincristine was increased by 0.1 nM. The resulting REH-VR cell line was treated with 2 nM vincristine on a weekly basis, and all experiments using REH-VR cells were carried out following 48 h of exposure to 2 nM vincristine. Additional chemotherapy-resistant REH ALL cell lines were developed as described for REH-VR, with the resulting REH-AR cell line resistant to 1 µM cytarabine (Ara-C) and REH-MR cell line resistant to 4.5 nM methotrexate (MTX). Concentrations of Ara-C, MTX and Vinc were based on previous work carried out in our group [7] as well as others [15,16]. De-identified primary normal donor stromal cells, acute myeloid leukemia (AML), and acute lymphoid leukemia (ALL) cells were provided by the WVU Cancer Institute Biospecimen Processing Core and the WVU Pathology Laboratory Tissue Bank. The REH cell line used in the current study was authenticated by short tandem repeat analysis (University of Arizona Genetics Core, Tucson, AZ) and maintained in 5% CO_2_ in normoxia at 37 °C.

### 2.2. Vincristine Resistance Stability

REH-VR cells were treated with 2 nM vincristine 48 h prior to the start of the experiment and were not grown in the presence of vincristine for the remainder of the experiment. Both REH and REH-VR cells were plated at 1 million cells/mL 48 h prior to the experiment set-up each week. After 48 h, cells were counted and plated at 50,000 cells per well in 100 µL volume in a 96-well flat-bottom plate, in triplicate, with or without 2 nM vincristine. After 48 h, viability was evaluated by Trypan Blue exclusion.

### 2.3. Cell Viability Studies

For the cell proliferation and viability studies, we performed this experiment by plating parental REH cells and REH-VR cells at 50,000 cells per well in 100 µL volume in a 96-well flat-bottom plate and counting the number of live and dead cells by trypan blue exclusion at the indicated time points. Apoptosis was measured using the Caspase-Glo 3/7 Glo kit (Promega) per assay instructions using white 96-well plates.

### 2.4. Cell Proliferation Assays

Cells were plated in 96-well flat-bottom clear plates at a density of 50,000 cells per well in 100 µL. Cells were exposed to pitavastatin at indicated concentrations, and proliferation was measured 48 h later using a Cell Counting Kit-8 (CCK-8/WST-8) (Dojindo Molecular Technologies, Rockville, MD), following the kit instructions. In this assay, we used 10 μL of the WST-8 reagent, which was added to each well; incubated for 120 min at 37 °C, and then read on the Cytation 5-plate (Bio-Tek) reader using the absorbance at 450 nm. Vehicle treated cells were considered to be controls. The IC_50_ was determined using a dose–response curve spanning seven log units and calculated using Prism 6 (GraphPad). For the combination treatment studies, we tested whether pretreatment of vincristine resistant REH-VR cells with pitavastatin would augment/sensitize the resistant cells to vincristine. Cells were pretreated with pitavastatin at a concentration of 450 nM for 24 h, after which vincristine (2 nM) was added to the indicated wells, and cell proliferation was measured 48 h later as described, without any media changes.

### 2.5. Co-Culture Experiments

Healthy donor bone marrow stromal cells were plated in 24-well plates, grown to 90% confluence, and then 100,000 REH or REH-VR were added to each well in 1 mL of medium. At days 4 and 8, 750 µL of medium was removed from the co-cultures and replaced with 750 µL of fresh medium. On day 8, pitavastatin was added to wells at the indicated concentrations for 72 h. On day 11, tumor cells floating in the medium and cells lightly adhered to the stroma were removed as one population for analysis. Trypsin was used to detach the stromal cells and the remaining REH cells physically associated with it, and cell viability was determined in both populations using Trypan Blue exclusion. Based on the significant difference in size, stromal cells were excluded from the viability counts.

### 2.6. Western Blot Analysis

Standard Western blot techniques were used. The REH or REH-VR cells were lysed in RIPA buffer and the protein concentration determined using a BCA kit (Pierce-Thermo-Fisher Scientific, Waltham, MA USA). An equal amount of protein was loaded onto a 4%–20% Mini-Protean TGX Precast Protein Gels (Bio-Rad Laboratories, Hercules, CA, USA) and then transferred to a nitrocellulose membrane. The latter membranes were blocked in Tris-buffer saline pH 7.4/0.1% Tween 20 (TBST) with a 5% nonfat dry milk for 60 min at RT. The membrane blots were then probed with a primary antibody overnight while at 4 °C, and then rinsed three times with TBST. Secondary antibodies with horseradish peroxidase conjugated were used to detect the primary antibody, with a 60 min incubation at RT, rinsed three times with TBST. The signal from the HRP was visualized using an Immobilon Western Chemiluminescent HRP Substrate (MilliporeSigma, Burlington, MA, USA) on an Amersham Imager (AI 680). The antibodies used in this study were (Cell Signaling Technologies (Danvers, MA, USA)): AMP-activated protein kinase alpha (AMPKα) (Cat#5832), pAMPKα (Cat#2535), Akt (cat#9272), pAkt (cat#4058), sirtuin 1 (SIRT1) (cat#9475), sirtuin 3 (SIRT3) (cat#2627), FoxO3a (cat#12829), pFoxO3a (Ser413) (cat#8174), pFoxO3a (Ser253) (cat#13129), Glut1 (cat#12939), TIGAR (cat#14751), poly-ADP ribose polymerase (PARP) (cat#9532), cleaved PARP (cat#5625), β-actin (cat#8457), Puma (E2P7G; cat# 98672), glyceraldehyde-3-phosphate dehydrogenase GAPDH (cat#5174), and anti-rabbit IgG horseradish peroxidase linked (cat#7074). Blots were stripped with Restore Western Blot Stripping Buffer (ThermoFisher, Waltham, MA cat#21059). When blots were stripped and re-probed, blots were washed with 1X TBST to remove chemiluminescent substrate and then incubated with 5 mL of stripping buffer for 10 min at 37⁰C. To validate that the stripping of antibodies worked, the blots were then washed with TBST before adding secondary antibody for 1 h at room temperature. After 1 h, blots were washed with TBST before imaging with chemiluminescent reagent to test for sufficient removal of antibody. Western blots are representative of at least three independent experiments (Amersham Imager 680 Analysis Software).

### 2.7. Cellular Respiration

The cellular respiration studies were carried out using a Seahorse XFe96 Bio-analyzer (Agilent Technologies, Santa Clara, CA, USA). (1) *Mito Stress Test assay:* sixty minutes before the assay, the cells were suspended in a Mito Stress assay medium (with glucose, L-glutamine, and sodium pyruvate) and transferred into Seahorse 96-well XF cell culture microplates which were coated with Cell-Tak Cell and Tissue Adhesive (Corning Inc., Glendale, AZ) at a density of 100,000 cells/well. The Cell-Tak solution was prepared to contain 0.09 M sodium bicarbonate, 0.02 N NaOH, and 3.3% *v/v* of Cell-Tak, with 10 µL added to wells; discarded after 20 min, followed by two 100 µL washes with water. Oxygen consumption rate (OCR) was measured using a sequential addition step of oligomycin, carbonyl cyanide p-trifluoromethoxyphenylhydrazone (FCCP), and lastly a combination of antimycin-A and rotenone. (2) *Glycolysis stress test*: a culture medium consisting of 49.5 mL of sterile XF Dulbecco’s Modified Eagle’s Medium, pH 7.4, and 500 µL of Seahorse XF 200 mM glutamine was added to the REH cells. The Seahorse 96-well XF cell culture microplate was centrifuged at 200× *g* for 60 s, placed in a non-CO_2_ incubator for 60 min prior to the start of the experiment to equilibrate. Glucose, oligomycin and 2-deoxyglucose were added per kit instructions to the cells during the assay. (3) *Beta Oxidation assay:* sixty minutes prior to the start of the assay, cells were suspended in assay media containing 48.5 mL of sterile XF DMEM Medium, pH 7.4 with 500 μL each of Seahorse XF 1.0 M glucose, Seahorse XF 100 mM pyruvate, and Seahorse XF 200 mM glutamine. Cells were seeded at a volume of 50 μL into the 96-well plate, then centrifuged at 200× *g* for 60 s and placed into a non-CO_2_ incubator for 30 min. Following this incubation, 130 μL of remaining DMEM-based assay media was added to the wells, bringing the total volume to 180 μL. The plate was again incubated for 30 min prior to beginning the experiment. OCR was measured. Etomoxir, oligomycin, carbonyl cyanide p-trifluoromethoxyphenylhydrazone (FCCP), and lastly antimycin-A/rotenone were added to each well per kit instructions with final concentrations of 4 μM, 1.5 μM, 1 μM, and 0.5 μM, respectively.

### 2.8. In Vivo Murine All Study

All animal procedures were reviewed and approved by the WVU Institutional Animal Care and Use Committee. Male NOD.Cg-*Prkdc^scid^ Il2rg^tm1Wjl^*/SzJ (NSG) mice aged 4–6 months were injected intravenously with either 1 × 10^6^ REH cells (*n* = 10) or 1 × 10^6^ REH-VR cells (*n* = 10). One day post-injection, the mice were randomized into two groups of five mice and treated with 10 mg/kg pitavastatin or vehicle control by i.p. injection every day for 4 weeks, similar to previous studies with compounds which are proposed to be utilized in combination with standard of care, for assessment of inhibition of cell proliferation [7,12,17,18,19,20,21]. Pitavastatin was dissolved in DMSO: polyethylene glycol 300 at 1:19; this latter vehicle was used for the control group; each mouse was dosed at 1 μL per gram, with each mouse receiving less than 5 μL of DMSO. At the end of 4 weeks, the mice were euthanized, femurs removed, and the bone marrow stained with PE-Cy5 mouse anti-human CD45 (cat#555484) purchased from BD Biosciences (San Jose, CA, USA). Samples were stained for 1 h at room temperature in phosphate-buffered saline (PBS) + 3% fetal bovine serum, washed with PBS, and then fixed in 1% paraformaldehyde for 1 h before resuspending in PBS for analysis of mean fluorescence intensity by flow cytometry (BD LSR Fortessa). The percent CD45+ cells were compared to the isotype control. The human antibody against CD45 was tested prior to use with bone marrow from mice, with mouse IgG1Kappa isotype PE-Cy5 (BD#555750) used as the isotype control.

### 2.9. Statistical Analysis

For the statistical analysis, Prism 6 and Instat 3 from GraphPad were used. All experiments were carried out with three or four independent experiments. All the data are represented as average + S.D., and a *p* < 0.05 was considered to be statistically significant. Statistical significance comparing three or more groups used a one-way ANOVA followed by a post-hoc Tukey’s test, while significance between two groups used a Student’s *T*-test.

## 3. Results

### 3.1. Development of a Vincristine-Resistant Cell Line

**Figure 1:**
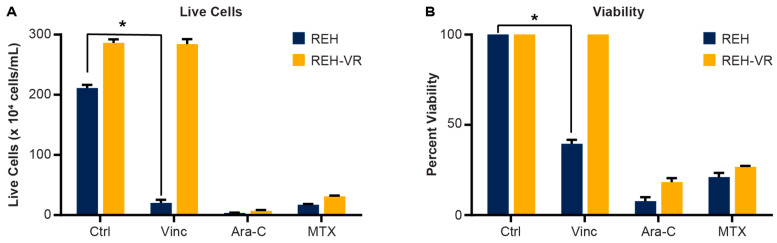
Development of a vincristine-resistant REH cell culture model. REH cells were cultured in the presence of increasing concentrations of vincristine to select for a vincristine-resistant cell line (REH-VR). The REH and REH-VR cells were treated with DMSO vehicle control (Ctrl), vincristine (Vinc; 2 nM), cytarabine (Ara-C; 1 μM) or methotrexate (MTX; 4.5 nM). A Trypan Blue exclusion assay shows the (**A**) number of live cells and (**B**) percent viability where the data have been normalized to the DMSO control viability set at 100% following treatment. While the REH-VR cells showed resistance to vincristine, they were still sensitive to other standard of care agents Ara-C and MTX. Each bar is an average ± S.D., where *n* = 3; * *p* < 0.05 denotes statistical significance.

Our lab generated a chemotherapy-resistant cell line (REH-VR) by exposing cells to increasing, sub-lethal doses of vincristine for several months. When treated with 2 nM vincristine, the parental control cells (REH) had significantly decreased viability, whereas REH-VR cells did not show any significant change in the number of live cells or viability (Figure 1). While REH-VR cells are resistant to vincristine treatment, they remain sensitive to other standard of care drugs, including Ara-C and MTX. The dose–responses of vincristine, Ara-C and MTX inhibition of cell proliferation with the REH, and the REH cells which were developed into resistant cells, are shown in Figure S1. While the IC_50_ indicated that Ara-C and vincristine become significantly less effective in preventing proliferation in the resistant cell lines, no change was seen with the MTX treatment.

### 3.2. Stability of Vincristine-Resistant Cell Line

We evaluated the stability of the chemotherapy-resistant cell line REH-VR to vincristine. After 6 weeks without weekly exposure to vincristine, REH-VR cells were still resistant to the chemotherapy drug vincristine with 100% viability maintained after exposure to 2 nM for 48 h (Figure S2).

### 3.3. Pitavastatin Reduces Cell Proliferation in Vincristine-Resistant REH Cells

**Figure 2:**
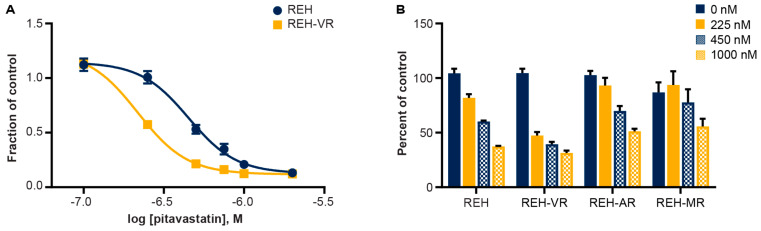
Pitavastatin reduces cell proliferation in leukemic cells. (**A**) REH and REH-VR were treated with pitavastatin for 48 h. (**B**) REH, REH-VR, REH-AR, and REH-MR cells were treated with pitavastatin for 48 h. The concentration of pitavastatin used was based on the IC_50_ values obtained from the inhibition curves (**A**), as well as an additional 2X the IC_50_ of REH. CCK-8 (WST-8) assay was used to determine cell proliferation. Data shown as fraction of control, or percent of control, when compared to vehicle control. The calculated IC_50_ values for the REH and REH-VR cells were 449 nM and 217 nM, respectively. Each symbol is average ± S.D., where *n* = 3.

We next measured the effect of pitavastatin on the proliferation of REH cells. Pitavastatin exposure of REH and REH-VR cells inhibited cell proliferation at an IC_50_ of 449 nM in REH cells and an IC_50_ of 217 nM in REH-VR cells (Figure 2A). Two additional REH cell lines were derived with resistance to other standard of care agents, Ara-C (REH-AR) and methotrexate (REH-MR). While pitavastatin reduced viability in all of the parental and chemoresistant cell lines, the REH-VR cells were the most sensitive to the lowest concentrations of this treatment (Figure 2B).

### 3.4. Effect of Pitavastatin on Mitochondrial Bioenergetics in Vincristine-Resistant REH Cells

**Figure 3:**
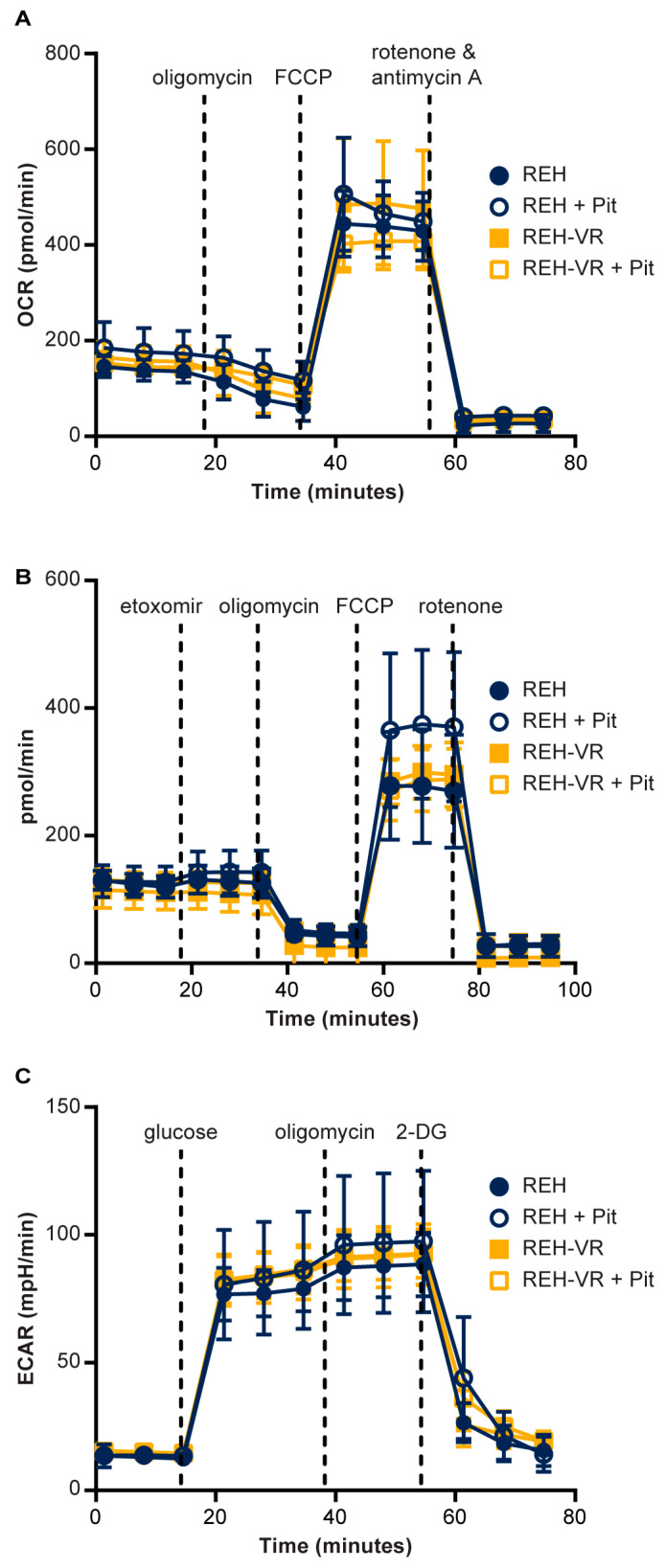
Pitavastatin treatment effects on mitochondrial bioenergetics. REH and REH-VR cells were treated with vehicle or 450 nM pitavastatin (Pit). Equal numbers of cells were plated in Seahorse 96-well plates. (**A**) Mitochondrial Stress Test. (**B**) Beta-oxidation assay. (**C**) Glycolysis assay. Pitavastatin did not significantly alter mitochondrial function in either cell type. Each data point represents average ± S.D., where *n* = 4. Abbreviations: 2-DG, 2-deoxyglucose; FCCP (carbonyl cyanide p-trifluoro methoxyphenylhydrazone); OCR (oxygen consumption rate); ECAR (extracellular acidification rate).

We then evaluated the effect of pitavastatin on cellular mitochondrial bioenergetics based on previous observations in a pancreatic cancer cell line that suggested mitochondrial involvement in pitavastatin’s mode of action [22]. A Mito Stress test assay showed pitavastatin treatment did not change the OCR in either REH or REH-VR cells (Figure 3A). To assess lipid metabolism-related activity in relation to mitochondrial function, cells were treated with pitavastatin and beta-oxidation activity was assessed (Figure 3B). There was a small increase in beta-oxidation observed in REH cells treated with pitavastatin, but this increase was not statistically significant, and pitavastatin did not affect beta-oxidation in REH-VR cells. No baseline differences in beta-oxidation were observed between the two cell lines in vehicle-treated cells. Finally, we evaluated the effect of pitavastatin on cellular glycolytic function and did not observe any differences between REH and REH-VR cells at baseline or with pitavastatin treatment (Figure 3C).

### 3.5. Pitavastatin Treatment Induces Apoptosis in Vincristine-Resistant Cells

**Figure 4:**
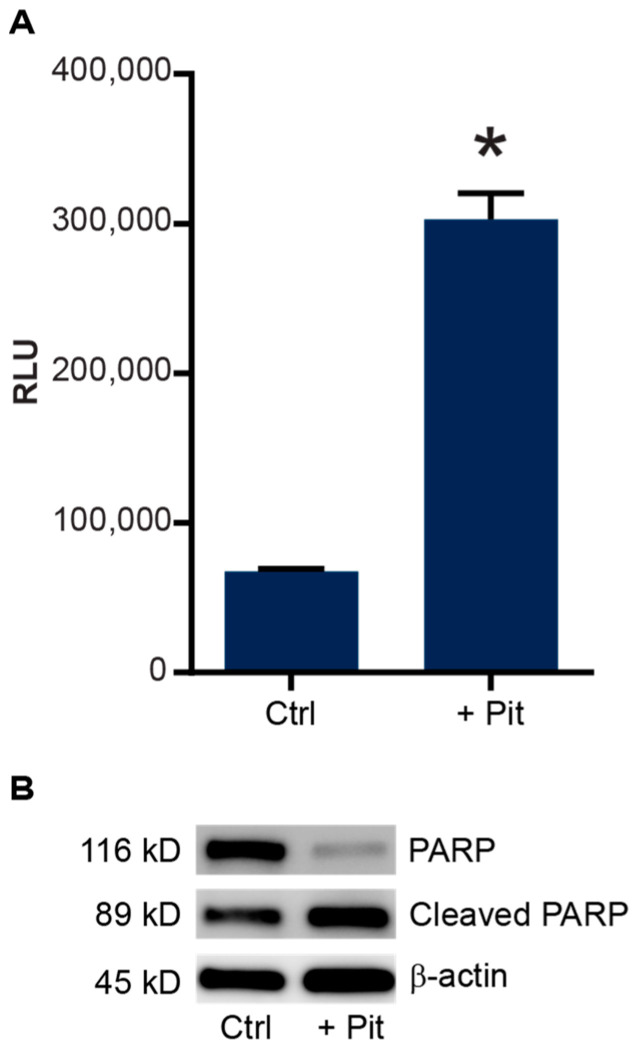
Pitavastatin treatment stimulates apoptosis in resistant cells. REH-VR cells were treated for 48 h with 450 nM pitavastatin (+ Pit) or DMSO vehicle control (Ctrl). (**A**) Increased activity of caspases-3 and -7 was observed in pitavastatin-treated REH-VR cells, where increase in relative luminescent units (RLU) indicated increased caspase activity. (**B**) Western blotting showed a decreased amount of PARP protein and an increased amount of cleaved PARP following treatment with pitavastatin. Bars represent average ± S.D., where *n* = 3; * *p* < 0.05 denotes statistical significance.

**Figure 5:**
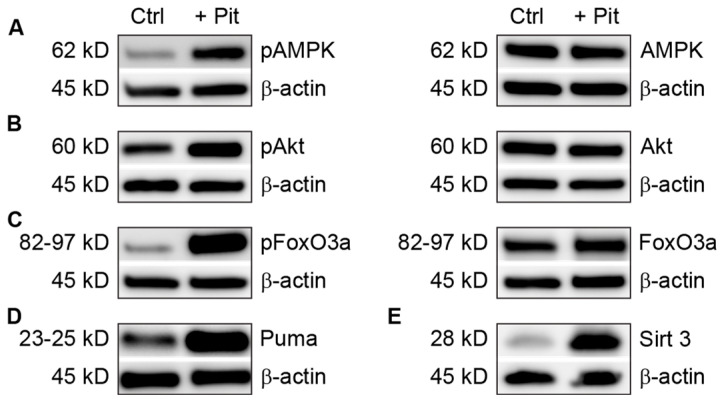
Pitavastatin effects on metabolic regulators. REH-VR cells were treated with 450 nM pitavastatin (+ Pit) or DMSO vehicle control (Ctrl). (**A**–**C**) Western blot analysis shows phosphorylated and total protein levels following 48 h treatment. (**D**) Puma and (**E**) Sirt3 expression were also increased with Pit treatment. Phospho-FoxO3a blot shown is for phospho-FoxO3a (Ser413).

Because previous studies with pitavastatin suggested that it induces apoptosis via a FoxO3a-mediated pathway [11], we examined the apoptotic-signaling effects of pitavastatin in REH-VR cells. Treatment of REH-VR cells with pitavastatin led to a significant increase in the activity of caspases 3 and 7 (Figure 4A). In addition, pitavastatin treatment resulted in increased cleaved PARP (2.0-fold over control) with a decrease in PARP (−1.7-fold compared to control) (Figure 4B). Taken together, these data suggest that pitavastatin induces apoptosis in REH-VR cells. We also evaluated the metabolic regulators AMPK, Akt, FoxO3a and SIRT3 in untreated and pitavastatin-treated REH-VR cells. Phospho-AMPK (1.8-fold increase), SIRT3 (2.9-fold increase), Puma (2.9-fold increase), phospho-AKT (2.9-fold increase) and phospho-FoxO3a (Ser 413) (7.6-fold increase) were increased with pitavastatin treatment as compared to vehicle controls (Figure 5). The same experiment was completed to evaluate the effects of pitavastatin on metabolic regulators in parental REH cells. Treatment with pitavastatin in the REH cells increased the levels of phospho-AKT, cleaved PARP, phospho-FoxO3a (Ser413) and Puma, all known regulators of apoptosis (Figure S3). Interestingly, we found that SIRT1, which is located primarily in the cytosol, was downregulated in REH-VR cells, whereas SIRT3, a mitochondrial sirtuin, was increased (Figure S4). We evaluated the effect of pitavastatin on primary patient samples of leukemia (Figure S5). Because pitavastatin is currently being tested in a clinical trial (NCT04512105) for AML, we compared the effect of pitavastatin treatment on AML as well as B-cell ALL patient samples. In both cases, we found an increase in phospho-FoxO3a protein, while FoxO3a was increased in AML only, consistent with the findings in the REH-VR cell line.

### 3.6. Pitavastatin Treatment Reduces the Proliferation of Leukemic Cells in a Co-Culture Model

**Figure 6:**
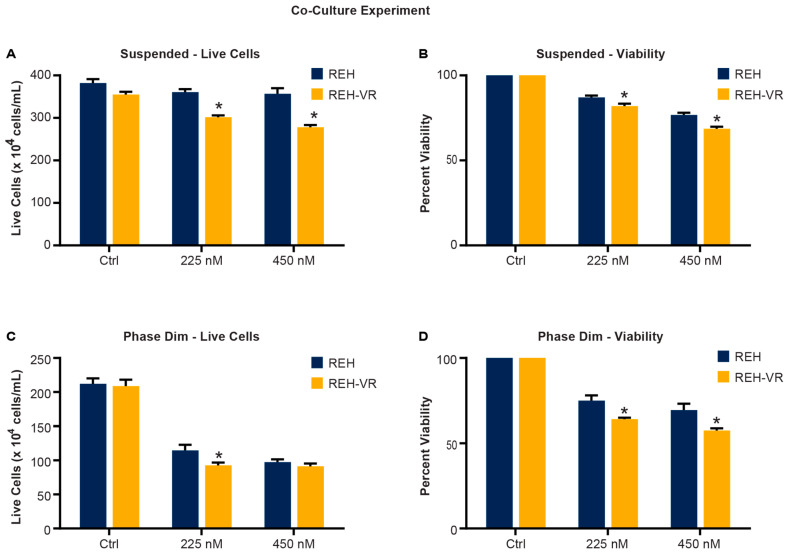
Co-culture model of leukemia cells with bone marrow stroma. Pitavastatin treatment reduced number of live cells in both the REH and REH-VR cells, at the concentrations tested. A Trypan Blue exclusion assay was used to analyze the suspended (S) (**A**,**B**) and phase dim (PD) (**C**,**D**) ALL cells from a co-culture following treatment with vehicle control (Ctrl) or pitavastatin (225 nM or 450 nM). Results are shown as the number of live cells (**A**,**C**) and the percent viable cells where the data have been normalized to the DMSO control viability set at 100% treatment (**B**,**D**). Each bar represents average ± S.D., where *n* = 3; * *p* < 0.05 denotes statistical significance when comparing REH and REH-VR for each treatment condition.

To evaluate whether pitavastatin would have the same effects in a more biologically relevant model, we co-cultured REH or REH-VR cells with human-derived bone marrow stromal cells [2,23,24]. This model mimics the hypoxic bone marrow microenvironment where leukemia cells reside in a protected niche and, thus, allowed us to consider pathways that may be relevant in the contribution of the microenvironment to MRD. We determined the effect of pitavastatin on two subpopulations of tumor cells within the co-culture: tumor cells suspended in the medium and tumor cells that had burrowed under the adherent bone marrow stromal cell layer, which were detectable with phase contrast microscopy and appeared dim (phase dim; PD) [2,3,23]. We observed a significant decrease in the number of live cells and total cell viability in the suspended and PD populations of the REH-VR co-culture treated with pitavastatin (225 nM and 450 nM) compared with the REH parental cells (Figure 6A–D). We thus observed a statistically significant reduction in cell proliferation in the PD population in the REH-VR co-culture treated with pitavastatin compared with the REH co-culture. While pitavastatin decreased the cell proliferation of PD tumor cells in both co-cultures, it was more effective in the co-cultured REH-VR PD cells. Lower numbers of live cells were recovered in the co-culture model, with a greater loss of cell proliferation, suggesting that pitavastatin might reduce cell proliferation in the bone microenvironment in vivo.

### 3.7. Combination Treatment with Pitavastatin and Vincristine In Vitro

**Figure 7:**
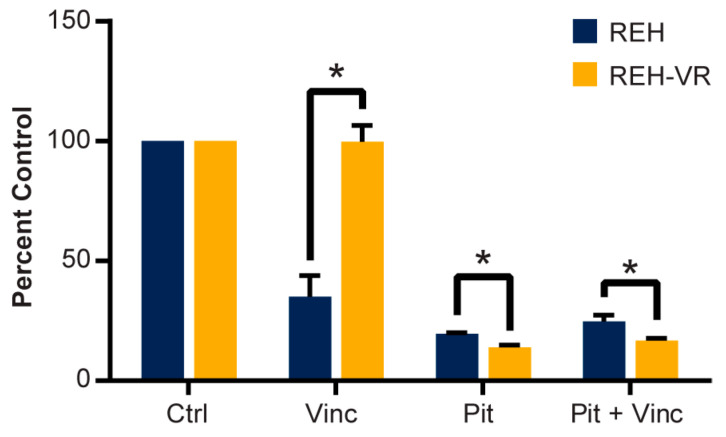
Combination treatment with pitavastatin and vincristine. REH and REH-VR cells were treated with either vehicle or pitavastatin (Pit, 450 nM) for 24 h, after which indicated cells were treated with either vincristine (Vinc, 2 nM), for an additional 48 h or total treatment time of 72 h and proliferation was determined with a CCK-8 (WST-8) assay. Pitavastatin did not show augmentation of vincristine activity. Each bar represents average ± S.D., where *n* = 3; * *p* < 0.05 denotes statistical significance.

To explore the effect of pitavastatin when combined in treatment with vincristine, we investigated the effects of concurrent treatment with pitavastatin and vincristine in both REH and REH-VR cells (Figure 7). As expected, vincristine treatment decreased cell proliferation in the parental REH cells but not in REH-VR cells. Treatment with pitavastatin alone significantly reduced cell proliferation in both REH and REH-VR cells. However, the combination of pitavastatin and vincristine did not further decrease cell proliferation compared with the effects of vincristine or pitavastatin alone in either cell line, indicating that the two drugs do not have an additive effect in this model system.

### 3.8. Pitavastatin Decreases the Number of Human CD45+ REH Cells in Mouse Bone Marrow

**Figure 8:**
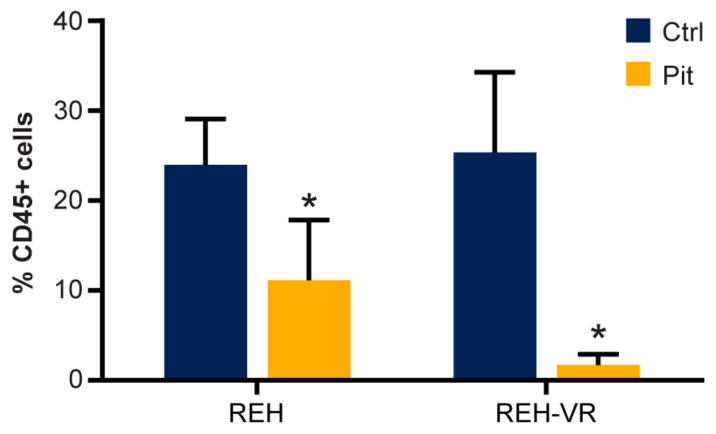
B-cell ALL mouse model. REH and REH-VR cells were engrafted in NSG mice. Mice were treated daily with pitavastatin (Pit, 10 mg/kg i.p.) for 4 weeks, while control mice were treated with vehicle (Ctrl). The percentage of human CD45+ ALL cells was determined in the bone marrow cells of mouse femurs. Pitavastatin significantly reduced the percentage of CD45+ cells in both parental and vincristine-resistant cells. No statistical difference was found for percent CD45+ cells between the REH and the REH-VR for vehicle control mice. Each bar represents average ± S.D., where *n* = 5 mice; * *p* < 0.05 denotes statistical significance.

Lastly, to determine if the effects of pitavastatin in vivo were similar to those observed in vitro, we injected REH and REH-VR cells into NSG mice and allowed the cells to engraft for 24 h. The mice were treated daily with pitavastatin (10 mg/kg) and after 4 weeks of treatment, bone marrow cells were collected. CD45 was used as the marker to identify the injected human cells that were recovered from the bone marrow of the mice. We did not find a difference between the number of CD45+ cells recovered in the REH or REH-VR vehicle control groups; there was a significant decrease in the number CD45+ cells in the pitavastatin-treated groups as compared with the vehicle control groups (Figure 8). Furthermore, this decrease in ALL cell numbers with the pitavastatin treatment was significantly larger in mice injected with REH-VR cells when compared to the mice injected with REH cells.

## 4. Discussion

In this study, we evaluated the effects of pitavastatin treatment on B-cell ALL using a chemoresistant cell line derived by long-term exposure to agents routinely used in the clinical setting. We found that pitavastatin treatment inhibited the proliferation of vincristine-resistant B-cell ALL (REH-VR) cells grown in monoculture as well as in a co-culture model with bone marrow stromal cells. Consistent with reduced viability, pitavastatin also decreased REH-VR total cell numbers recovered in vivo in a murine model of ALL.

Pitavastatin is an FDA-approved drug belonging to the statin family of compounds that is routinely used as a lipid-lowering medication [25]. Recently, pitavastatin has been approved for use in a phase I clinical trial (NCT04512105) in combination with venetoclax in patients with AML and chronic lymphocytic leukemia. One literature review [10] indicated that pitavastatin has been evaluated in several other cancer types, including lung [26,27], prostate [22], colon [21], and liver [12,28]. These studies have identified several mechanisms leading to apoptosis with pitavastatin alone, or in combination with other therapeutics, including targeting of the Ras/Raf/MEK or Akt-mediated signaling pathways [27]. In colon cancer, pitavastatin decreased the expression of several stem cell markers and induced apoptosis. These findings were further supported by studies in an in vivo xenograft model of colon cancer, where mice receiving pitavastatin exhibited a reduction in tumor growth [21]. Pitavastatin holds promise as a possible anti-leukemic therapy, as it has been shown to be well tolerated and safe in children and adolescents [29], and is routinely used clinically in adults [30].

B-cell ALL is a hematologic malignancy that affects both pediatric and adult patients. A clinically significant problem with B-cell ALL is the development of chemoresistance, leading to MRD and subsequent relapse. Several studies have shown that the bone marrow microenvironment can contribute to chemoresistance and MRD in a variety of ways, one of which is providing chemical cues that allow the leukemia cells to maintain a relatively low metabolic profile. Targeting this niche remains challenging, and identifying a compound that could be used either synergistically with current treatments, or as a single agent in patients with chemoresistant disease, is critical to improving patient outcomes. Previous work published by our group demonstrated that targeting metabolic pathways is an attractive avenue to explore, and may provide an alternative method of eradicating residual, chemoresistant disease [7,8,18,24,31,32].

In the current investigation, we focused on the development of resistance to the chemotherapy compound, vincristine, since this drug is part of the standard of care in B-cell ALL patients [33,34,35,36,37]. Other standard-of-care compounds include Ara-C and MTX. Ara-C is an antimetabolite, whereas MTX inhibits dihydrofolate reductase, both leading to a decrease in DNA synthesis. The REH-VR tumor cell line remained resistant to subsequent vincristine exposure, but exhibited sensitivity to both Ara-C and MTX. Based on the work of other teams suggesting that cancer cell metabolism may contribute to chemoresistance [38,39] in addition to the well-described efflux mechanisms with ABC Transporters [40], we evaluated the protein expression level of several key proteins related to metabolic activity, including the glucose transporters, Glut1, TIGAR (*T*P53-*i*nduced *g*lycolysis and *a*poptosis *r*egulator), and the Akt kinases. 

The finding that the IC_50_ of pitavastatin was lower in the REH-VR cells (217 nM) than in the REH cells (449 nM) was surprising since we expected the REH-VR cells to have metabolic adaptations that might circumvent the effects of pitavastatin treatment. Several studies have suggested that altered mitochondrial function and lipid metabolism may contribute to chemoresistance [38,41]. A pitavastatin plasma concentration of the REH-VR cells can be achieved as seen from pharmacokinetic studies previously published [42]. In our studies of mitochondrial bioenergetics, we observed no significant difference in oxygen consumption through beta-oxidation, or in glycolytic function, in the REH or REH-VR cells. These findings suggest that the mechanisms of vincristine resistance might involve other cell survival signaling pathways. Although other studies have shown that pitavastatin can restore the beta-oxidation of free fatty acids in models of hepatic steatosis, no significant changes in beta-oxidation were observed in our resistant leukemic cell model [43].

**Figure 9:**
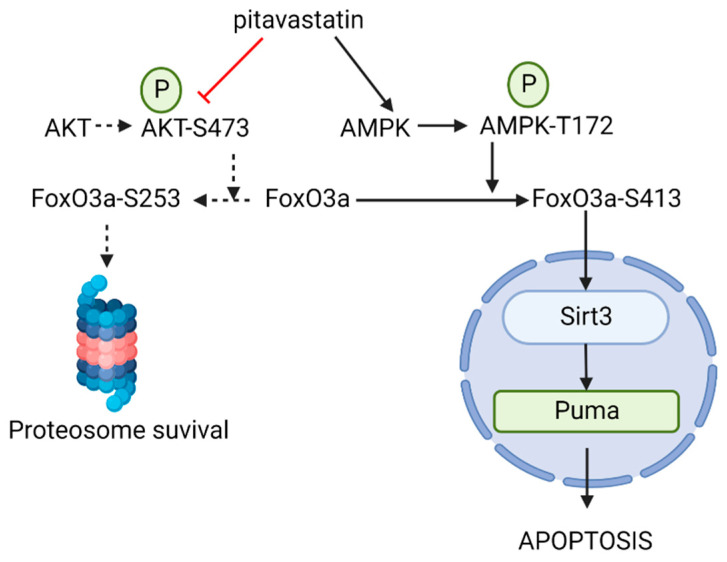
Pitavastatin in B-cell ALL. Pitavastatin treatment leads to increased activation of FoxO3a phosphorylation on S413, leading to Sirt3/Puma-mediated cell death. The AKT-mediated protection pathway was not activated with the treatment of pitavastatin [11]. Drawn using Biorender.

To gain further insight into the activity of pitavastatin in our REH-VR cells, we evaluated the expression levels of several proteins involved in metabolic signaling [11]. We found increased levels of phospho-AMPK, which is considered to be an energy sensor/master regulator in cancer cells [44,45]. We also found increased expression of SIRT3, which is one of the NAD(+)-dependent enzymes found in mitochondria [46]. In AML, the increased protein expression of SIRT3 has been associated with chemoresistance and the reprogramming of mitochondrial bioenergetics (oxidative phosphorylation) [47]. Based on the observed decrease in cell proliferation in leukemic cells treated with pitavastatin, we next determined the protein expression level of FoxO3a, a transcription factor associated with apoptosis and decreased cell proliferation [48]. The evaluation of phospho-FoxO3a showed phospho-FoxO3a (Ser413) was increased, but not phospho-FoxO3a (Ser253), which indicated an AMPK-Sirt3 pathway instead of an AKT-FoxO3a interaction. We found that FoxO3a phosphorylation was increased in REH-VR cells treated with pitavastatin, as well as Puma; the latter leads to apoptosis when triggered by FoxO3a. The evaluation of cleaved PARP and caspase 3/7 activity indicated that pitavastatin reduces cell proliferation via an apoptotic mechanism [11]. Similarly, we found that pitavastatin treatment of primary AML and ALL led to similar increases in phospho-FoxO3a. Our findings suggest that, similar to other studies, the AMPK/FoxO3a signaling pathways play a role in ALL chemotherapeutic activity (Figure 9).

The bone marrow microenvironment is a protective niche that provides a sanctuary for leukemic cells and contributes to poor prognostic outcomes in leukemia patients who develop chemoresistance [1,2,3,4,18,49,50,51,52,53,54,55]. To recapitulate one key element of this niche, the stromal cell contribution, and evaluate novel therapeutics in a more clinically relevant in vitro system, we developed a co-culture model [24,53]. In this model, pitavastatin treatment significantly reduced the numbers of live, drug-resistant REH-VR cells as compared with the effects on REH cells. These findings suggest that pitavastatin or similar compounds may be useful in eradicating chemoresistant MRD in the protective bone marrow microenvironment. Interestingly, pitavastatin treatment did not restore sensitivity to vincristine in REH-VR cells, suggesting that its therapeutic use would be as a single agent as part of a treatment regimen at diagnosis, or as maintenance therapy to eradicate chemoresistant cells and prevent relapse after completion of the standard of care regimen. Observations in vitro provided support for evaluating pitavastatin in a murine model of leukemia. The in vivo model corroborated our in vitro findings, in that mice engrafted with REH-VR cells that were treated with pitavastatin showed decreased levels of human CD45+ ALL cells in the bone marrow as compared with control (vehicle-treated) mice. At the end of the murine study, similar numbers of CD45+ cells were found in the vehicle control groups, resulting in similar tumor burdens, suggesting similar engraftment of the ALL cells, although more in-depth studies at different timepoints will need to be carried out to fully evaluate the mechanisms of engraftment. Additionally, tumor engraftment validation before pitavastatin treatment was not carried out in the current study, which is a caveat. As noted with earlier in vitro-based experiments, REH-VR cells were notably very sensitive to pitavastatin compared to the parental REH cell line which also responded, but to a lesser degree. Interestingly, we found a greater decrease in the tumor burden in mice injected with REH-VR cells with pitavastatin treatment, as compared to the REH injected mice corroborating the in vitro studies. Taken together, our data suggest that pitavastatin may have utility in reducing proliferation from chemoresistant MRD within the bone marrow niche, due to the more potent inhibition of the REH-VR cells. Limitations to the current study which need to be explored in future studies are the use of the pharmacological inhibition of AMPL, FoxO3a as well as PUMA, as well as the genetic knockdown of these proteins to confirm the cellular mechanism of pitavastatin in REH-VR cells.

Finally, we evaluated the effect of pitavastatin on primary patient samples of leukemia (Figure S4). Because pitavastatin is currently being tested in a clinical trial for AML, we compared the effect of pitavastatin treatment on AML as well as B-cell ALL patient samples. In both cases, we found an increase in phospho-FoxO3a protein, consistent with the findings in the REH-VR cell line.

## 5. Conclusions

The current study demonstrates for the first time that pitavastatin is an effective chemotherapeutic agent in models of chemoresistant B-cell ALL. Pitavastatin reduced the proliferation of REH-VR cells at a lower concentration than required to affect the parental REH cells, demonstrating a unique vulnerability in a biologically relevant model of tumor cells that could contribute to relapse of disease. Additionally, pitavastatin was effective in reducing the proliferation of REH-VR cells not only in media, but in a co-culture model with protective bone marrow stromal cells, and in vivo with a murine model of ALL. Taken together, our data suggest that exposing vincristine refractory ALL cells to pitavastatin may be an efficacious addition to treatment strategies in patients with chemoresistant B-cell ALL populations. Future studies will investigate additional B-cell ALL cell lines (Philadelphia-positive (Ph+) and Philadelphia-negative (Ph−)), as well as mechanistic and dosing-regimens identified here, in the bone marrow of an in vivo mouse model of ALL.

## Data Availability

The data supporting the findings of this study are available upon request.

## Supplementary Materials

The following supporting information can be downloaded at: https://www.mdpi.com/article/10.3390/cancers15030707/s1, Figure S1: Dose-response effect of chemotherapeutics on cell proliferation, using CCK-8 (WST-8) reagent; Figure S2: Stability of vincristine resistance; Figure S3: Pitavastatin effects on metabolic regulators; Figure S4: Differential expression of proteins in the vincristine-resistant cells; Figure S5: Pitavastatin treatment of primary leukemia samples.
