# Supplementary material for: Chemotherapeutic Activity of Pitavastatin in Vincristine Resistant B-Cell Acute Lymphoblastic Leukemia
Source: Cancers (Basel). 2023 Jan 24;15(3):707. doi: 10.3390/cancers15030707 (PMC9913300; doi:10.3390/cancers15030707)
Supplement: Supplementary file 1 [file cancers-15-00707-s001.zip › cancers-2070903-original-images.pdf]

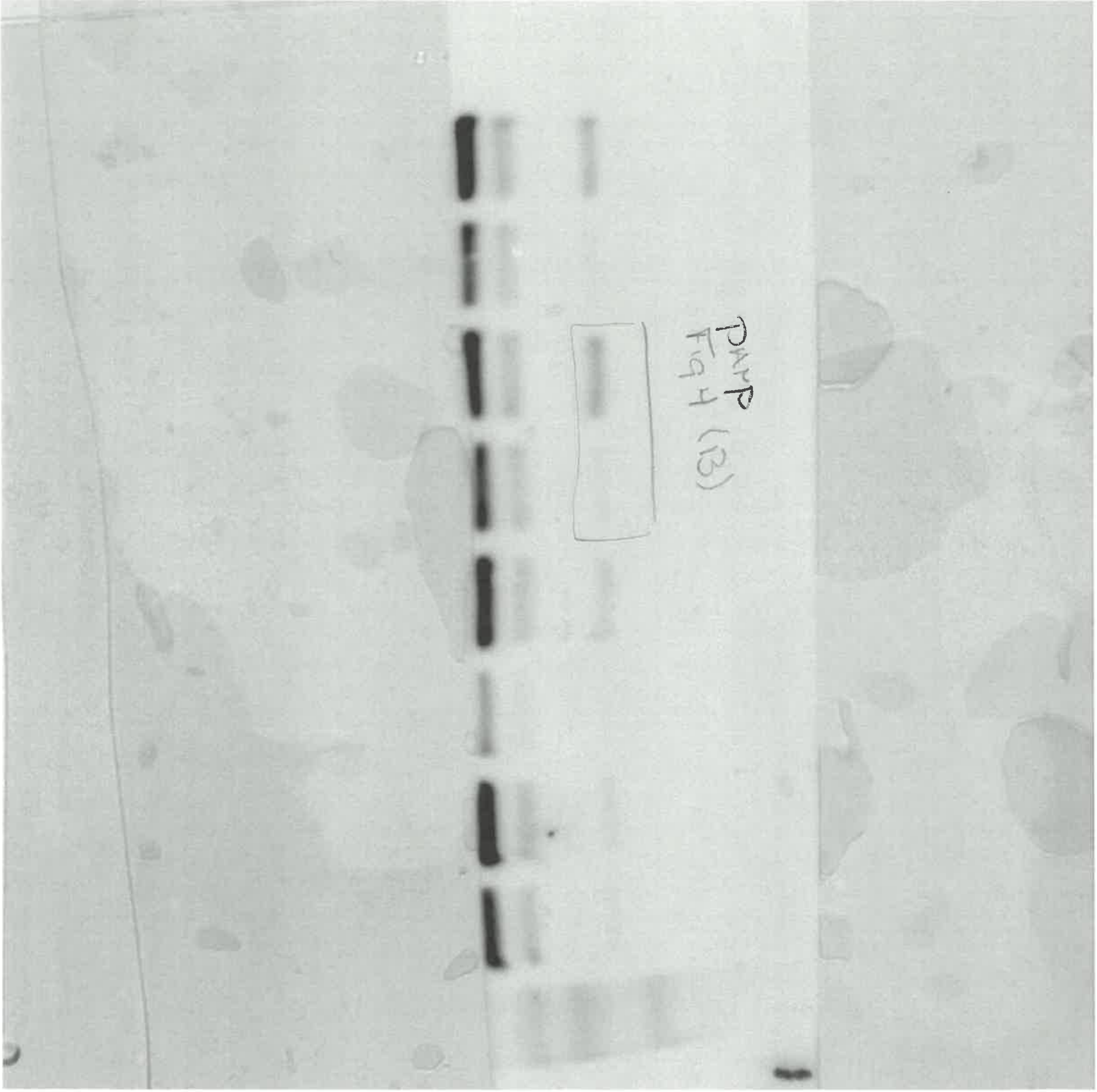
PARP
Fig 4 (B)

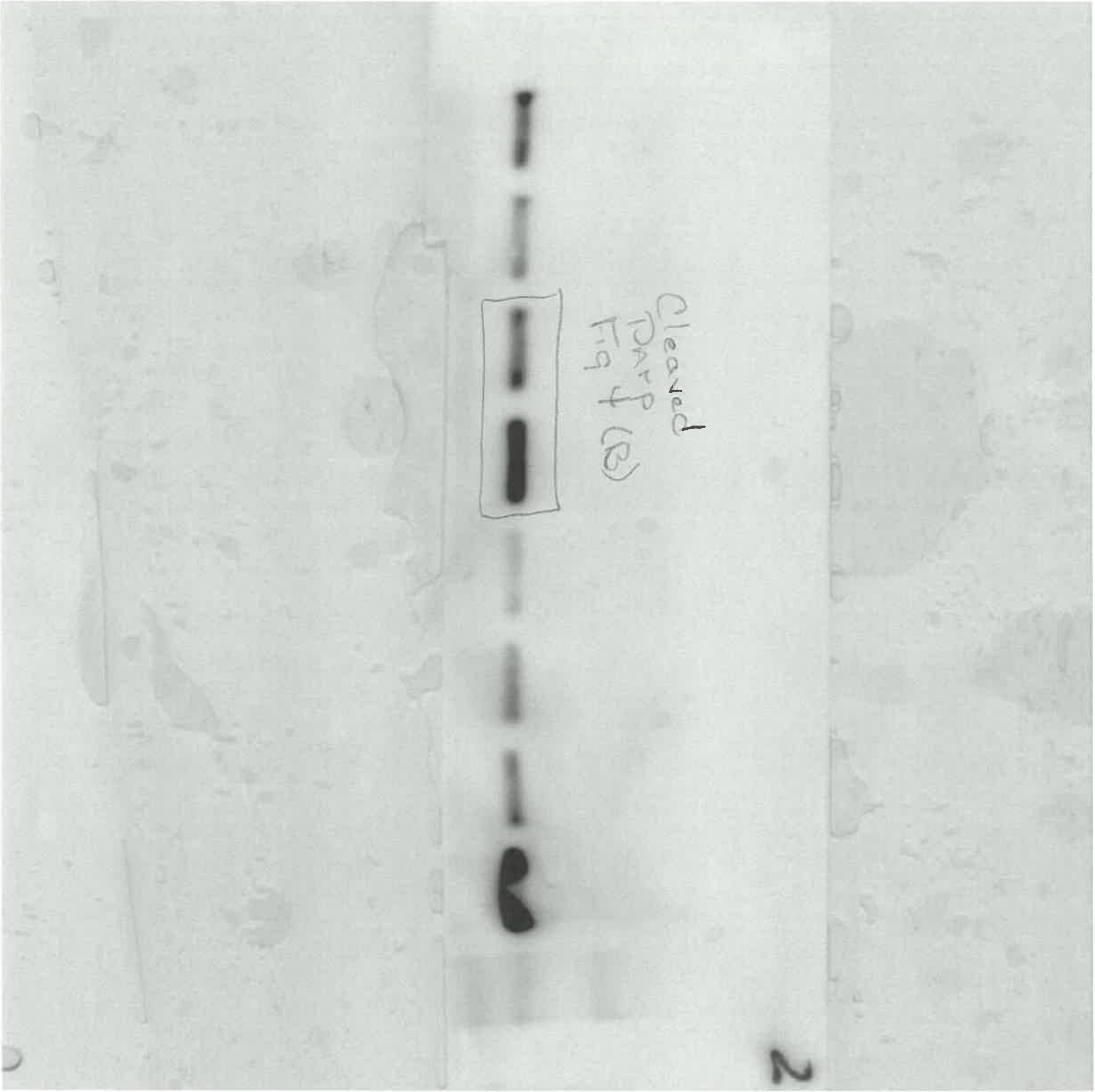
Cleaved
Parp
Fig 4 (B)
2

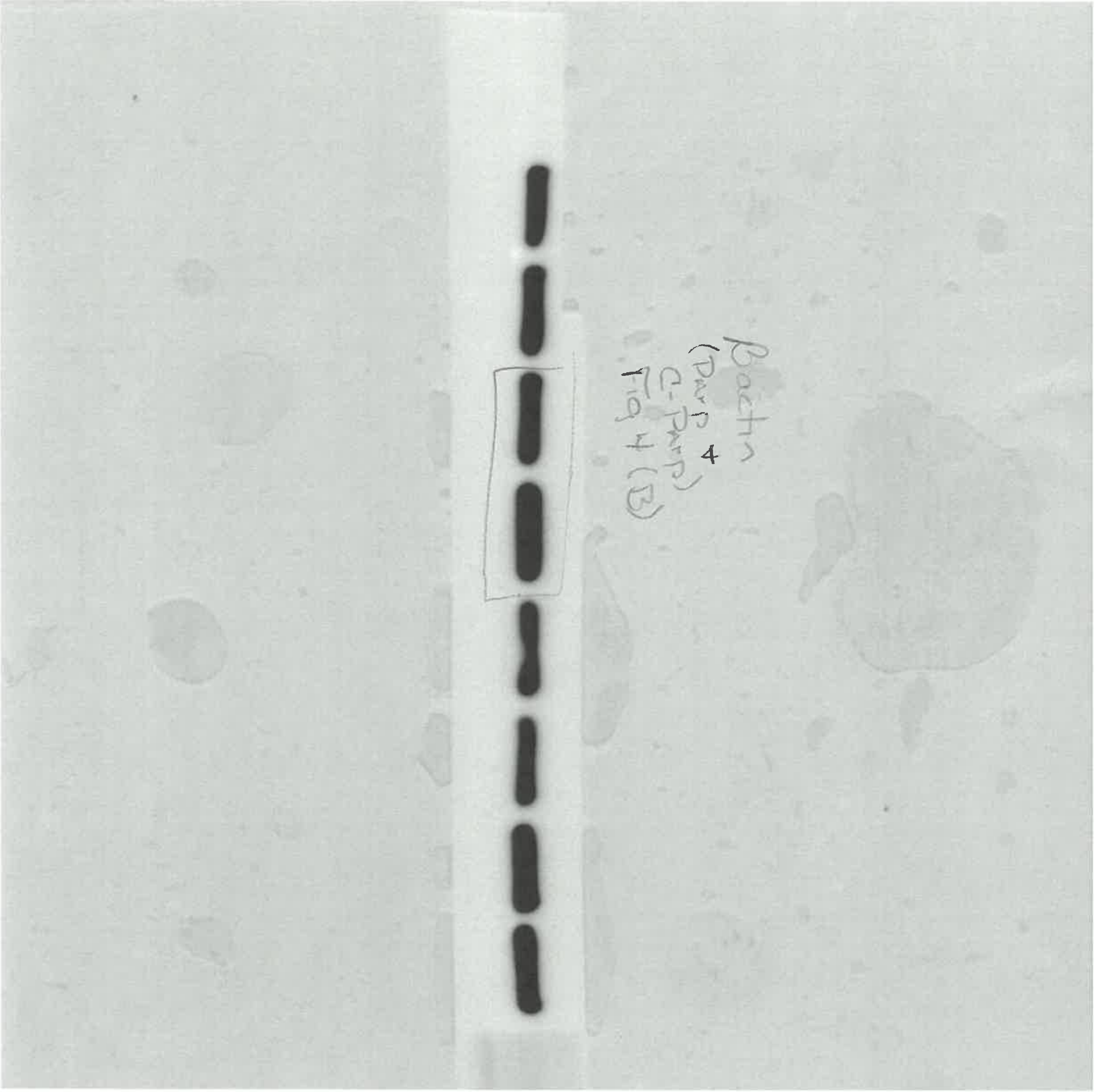
Bactin
(Parp
C-Parp)
Fig 4 (B)

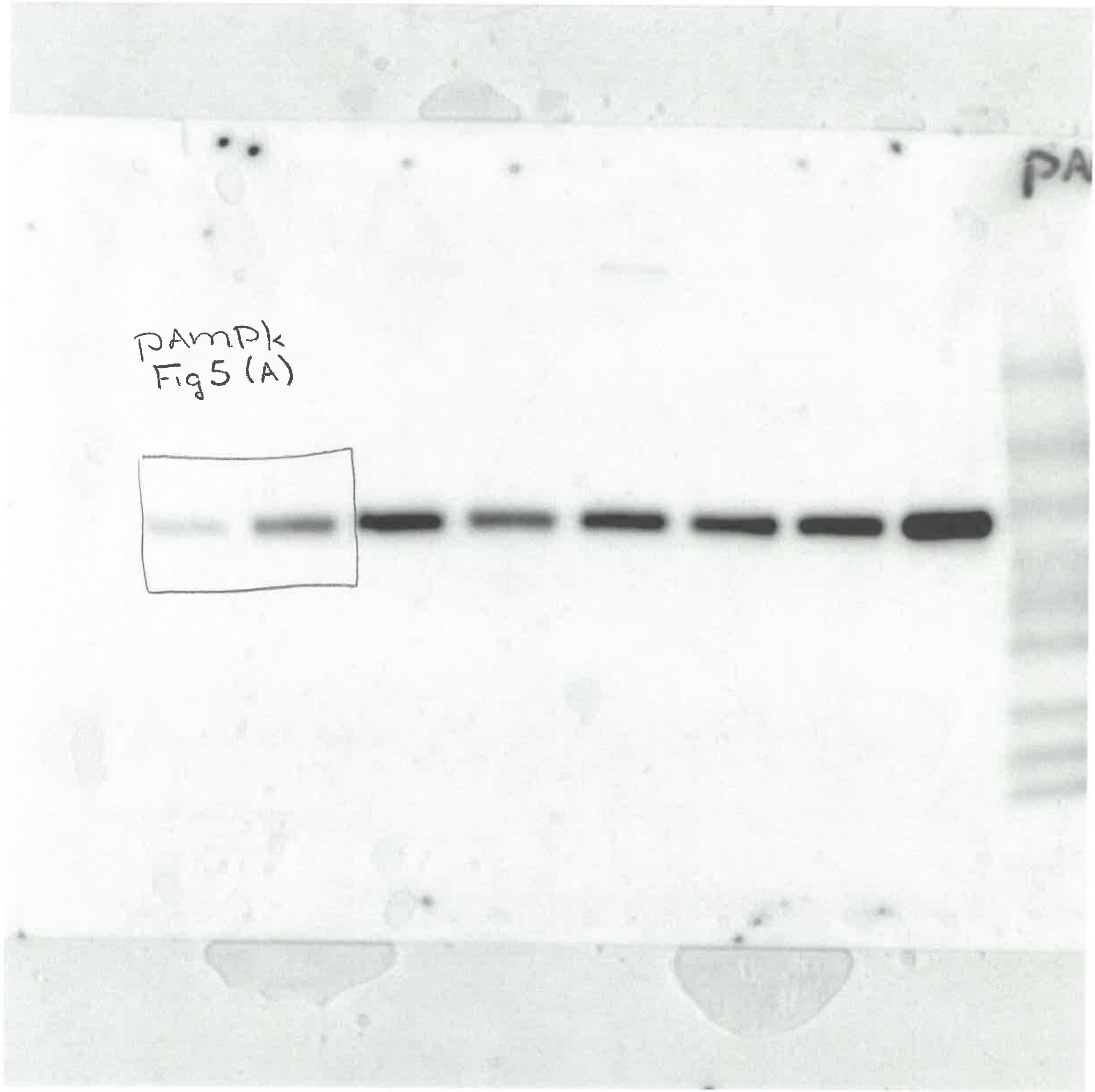
pAmPk
Fig 5 (A)
PA

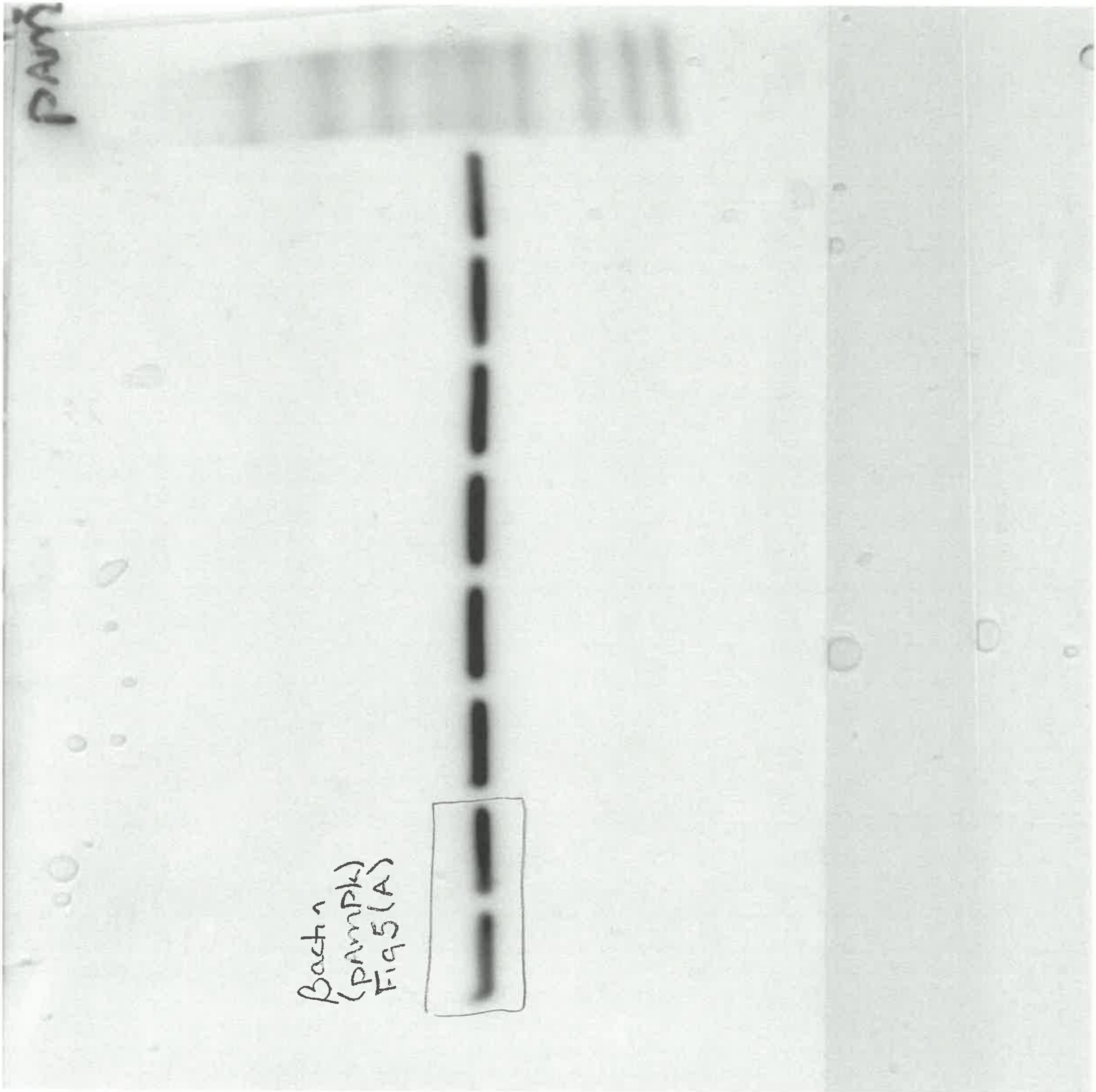
pAM
Bactin
(pAMPK)
Fig 5(A)

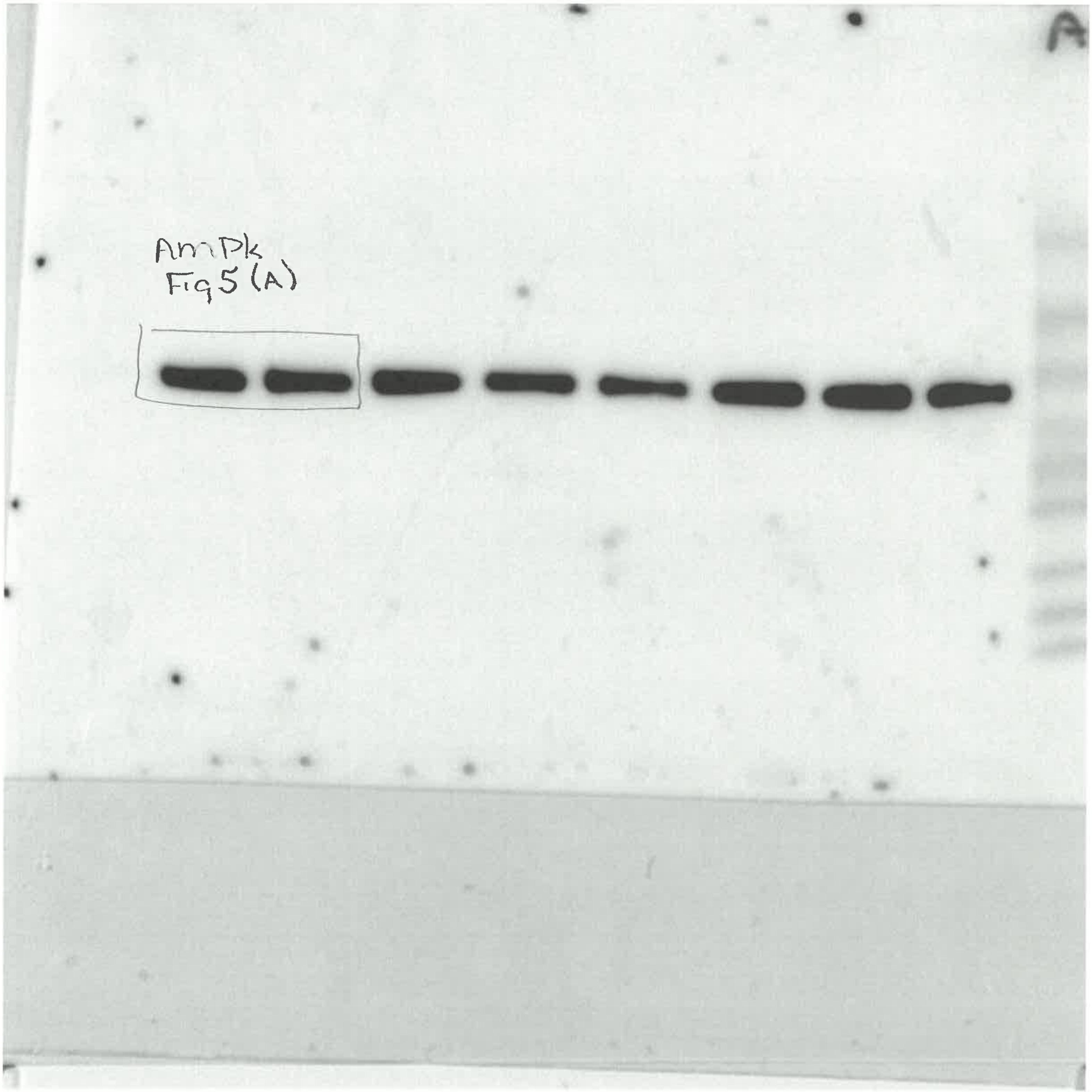
AmPk
Fig 5 (A)
A

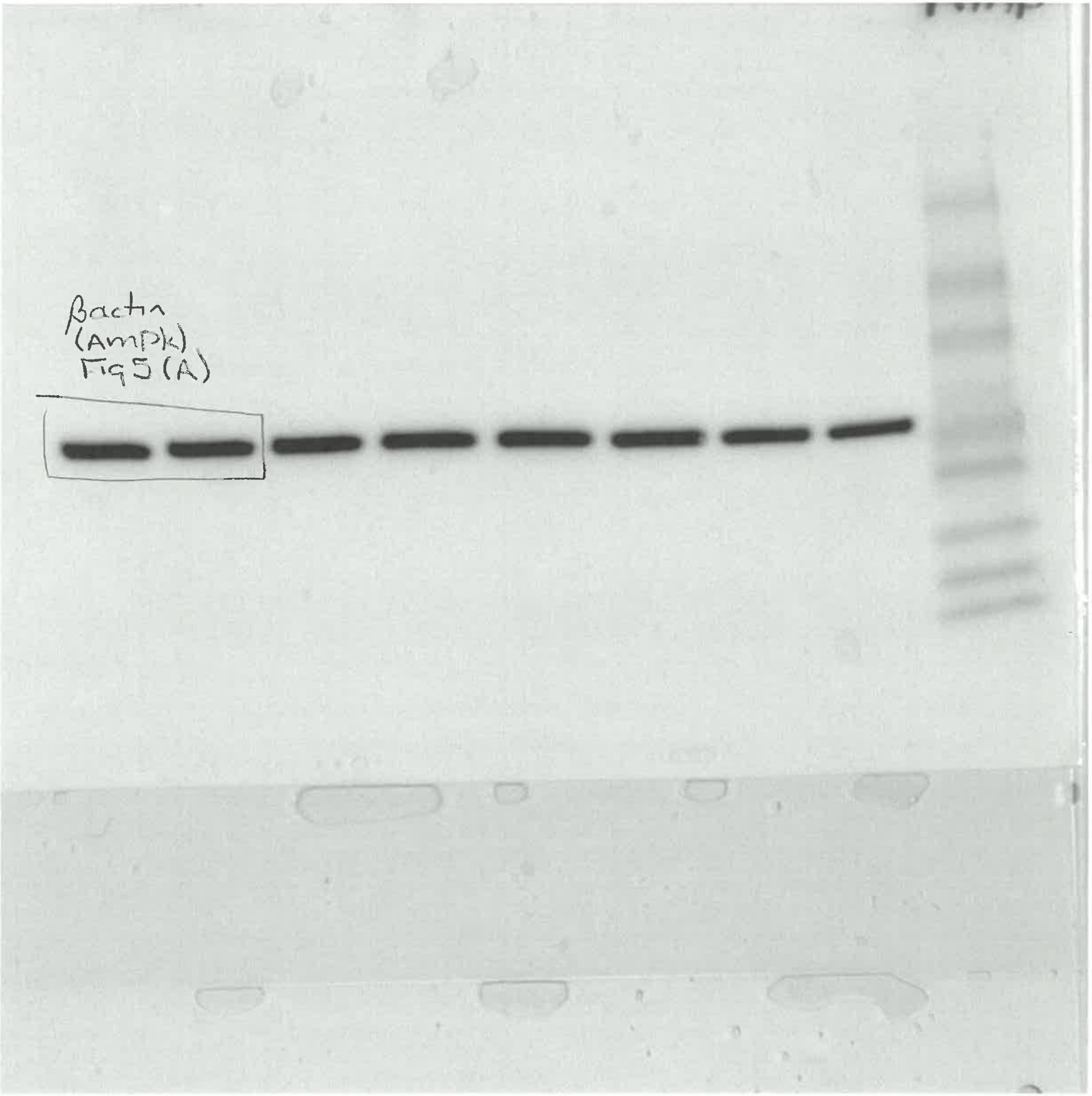
βactin
(Ampk)
Fig 5 (A)

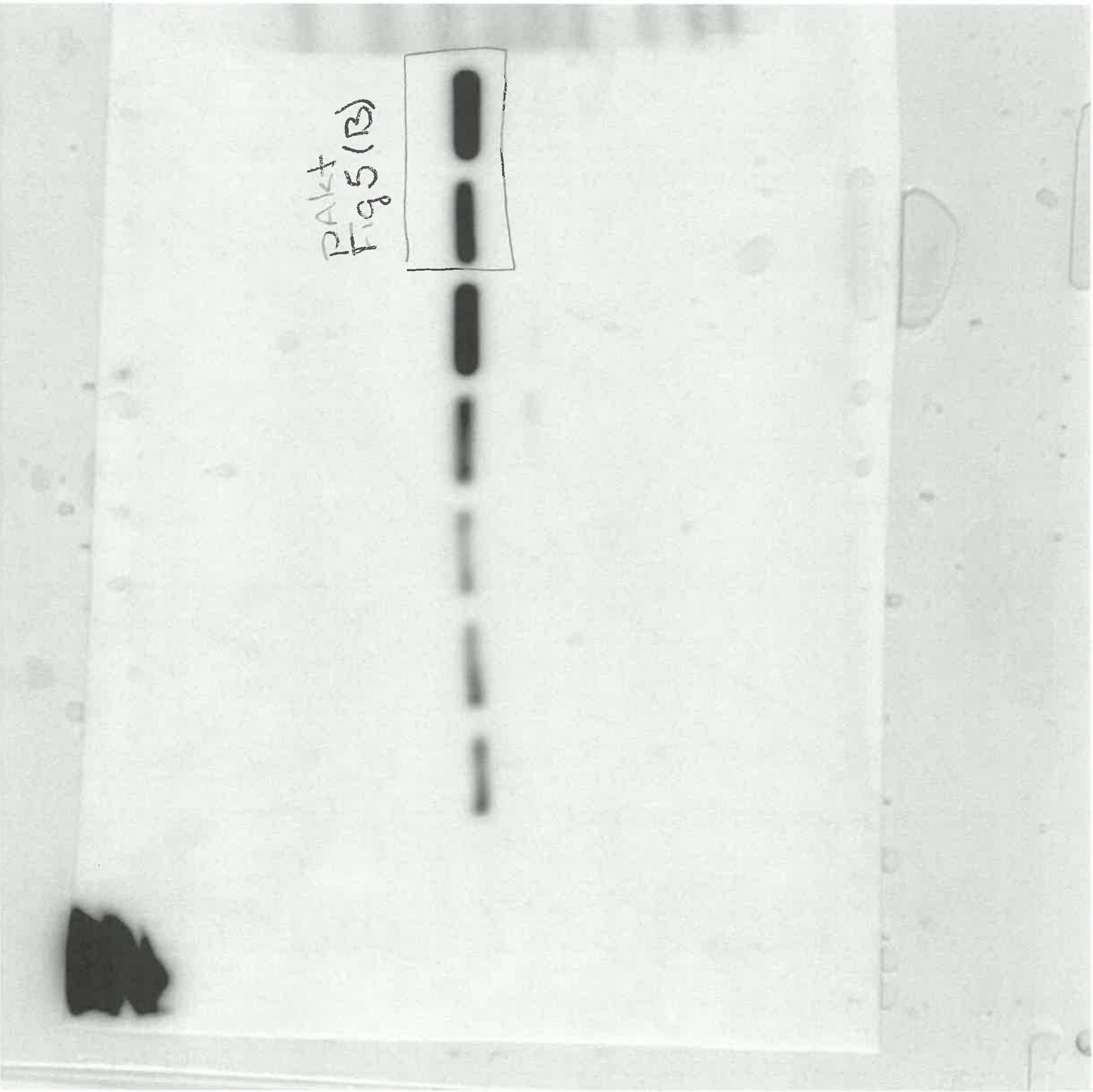
pAkt
Fig 5(B)

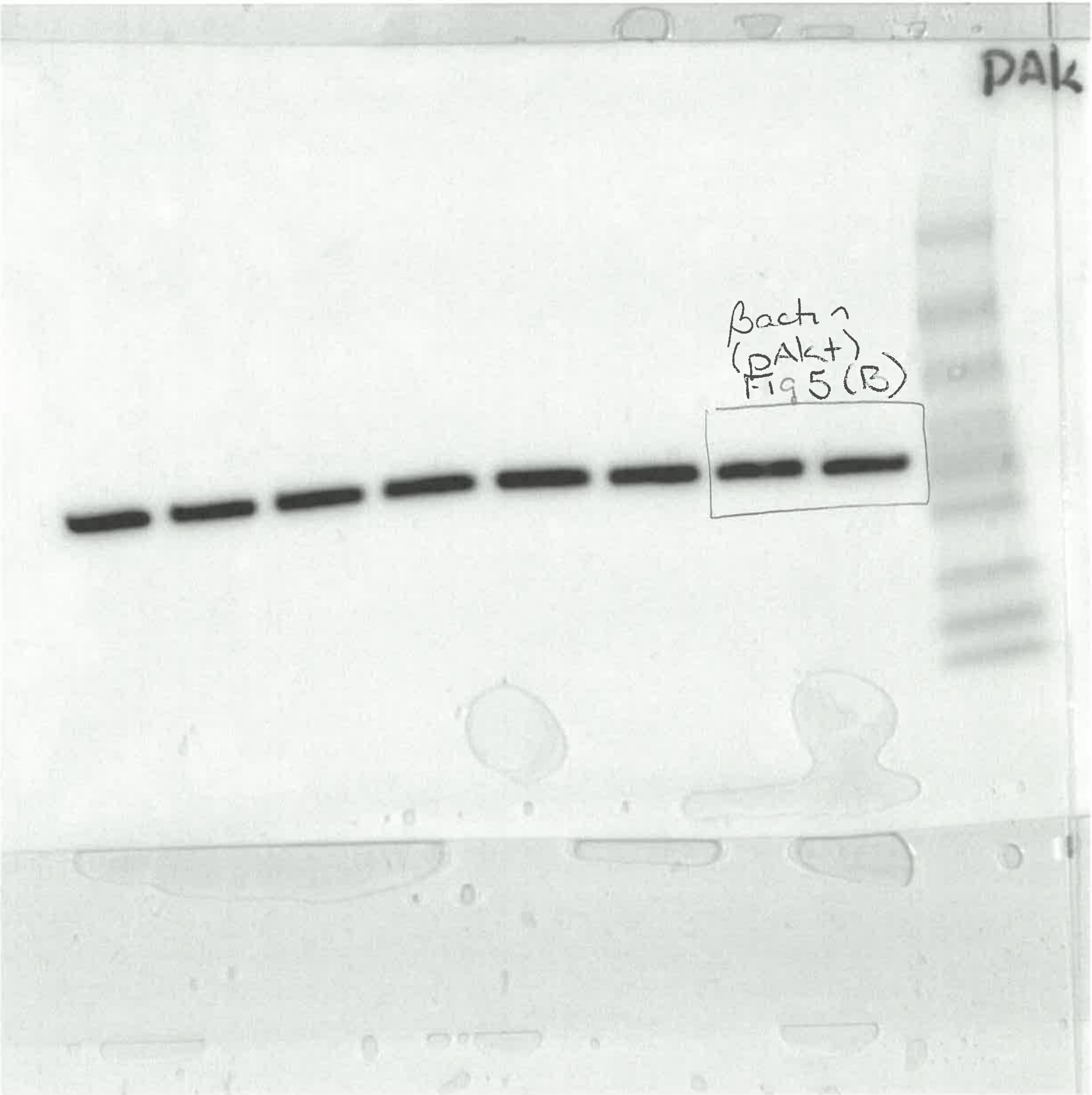

PAK
βactin
(pAkt)
Fig 5 (B)

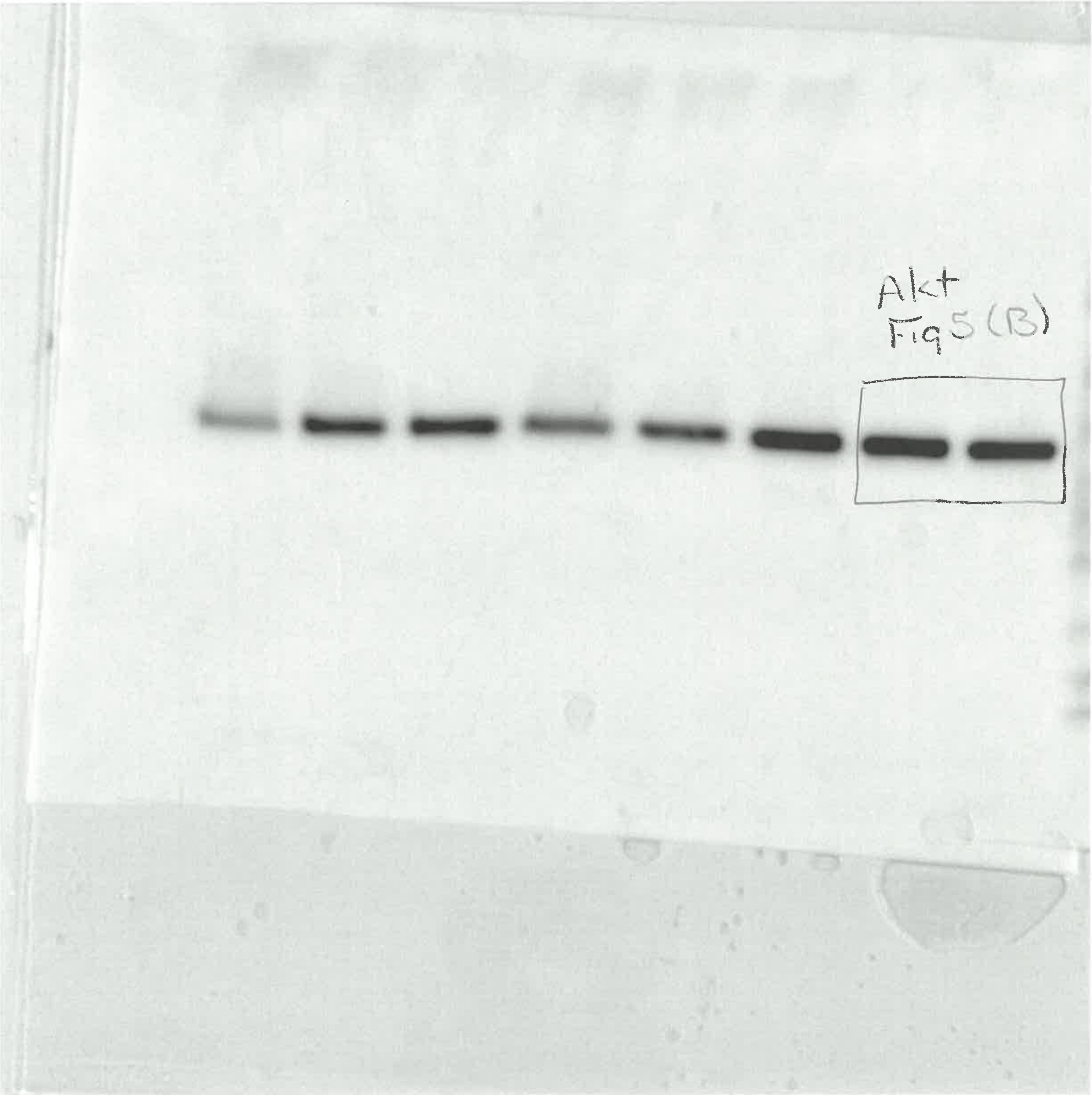
Akt
Fig 5 (B)

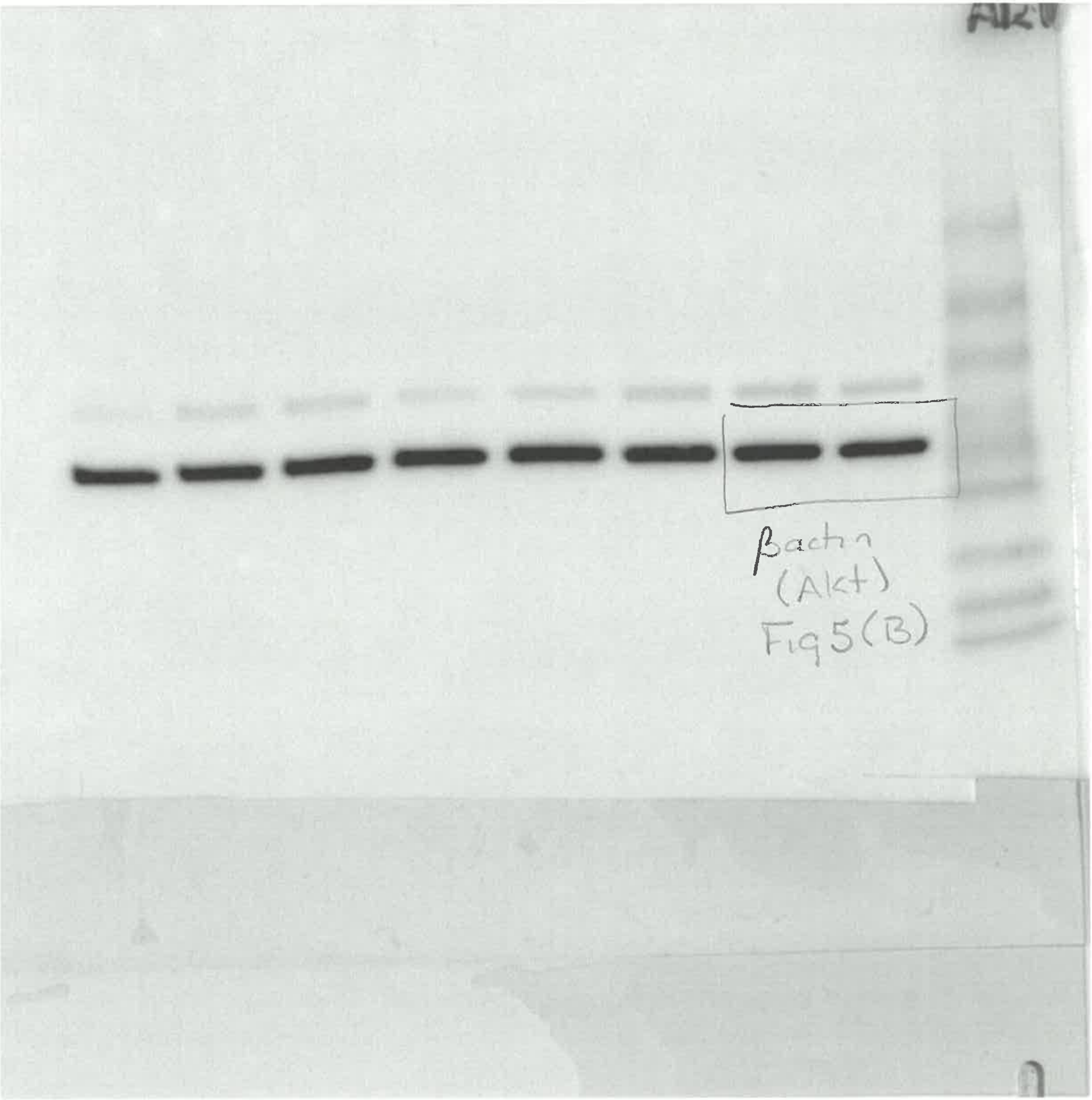
βactin
(Akt)
Fig 5(B)

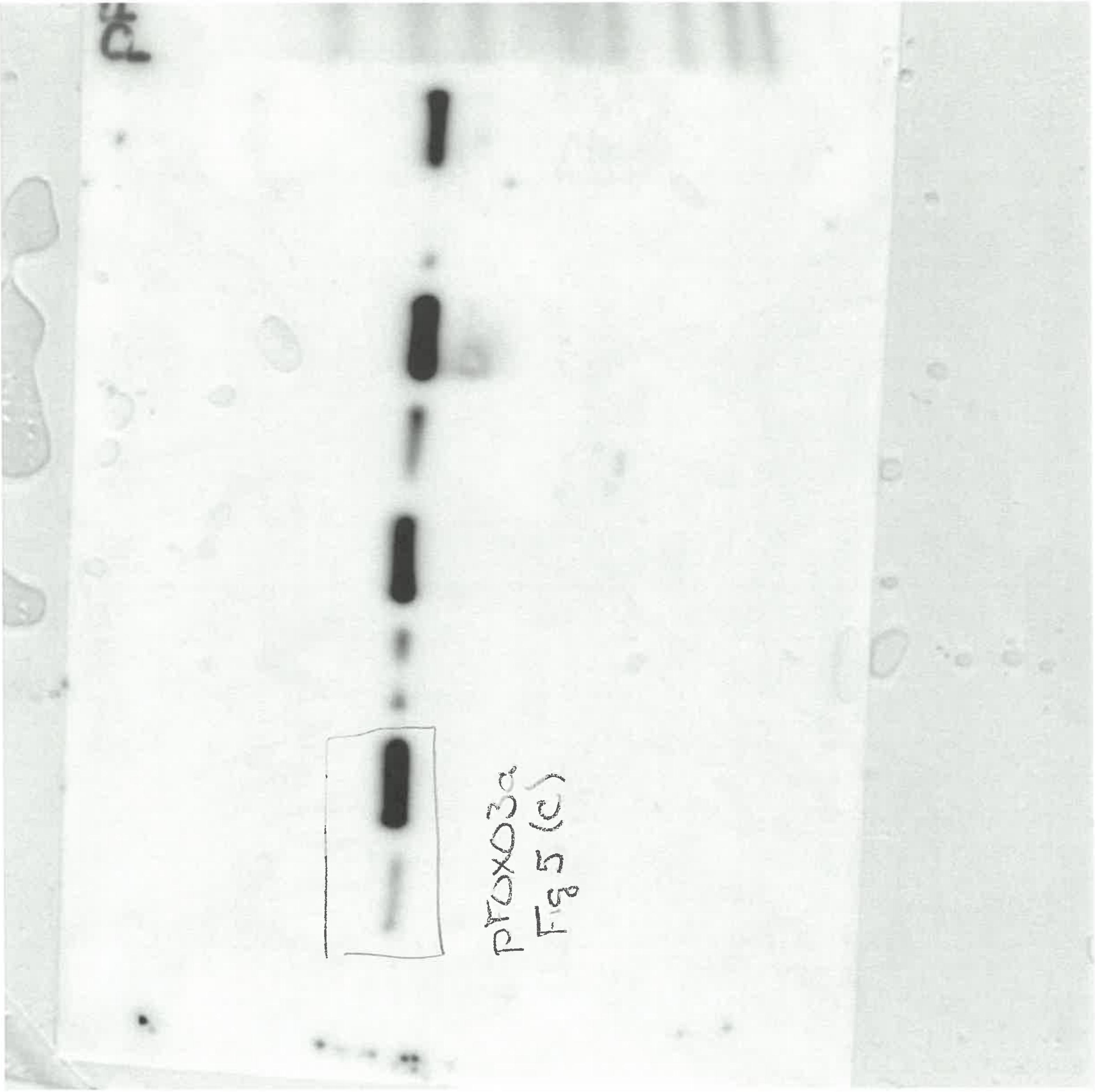
pFOXO3a
Fig 5 (c)

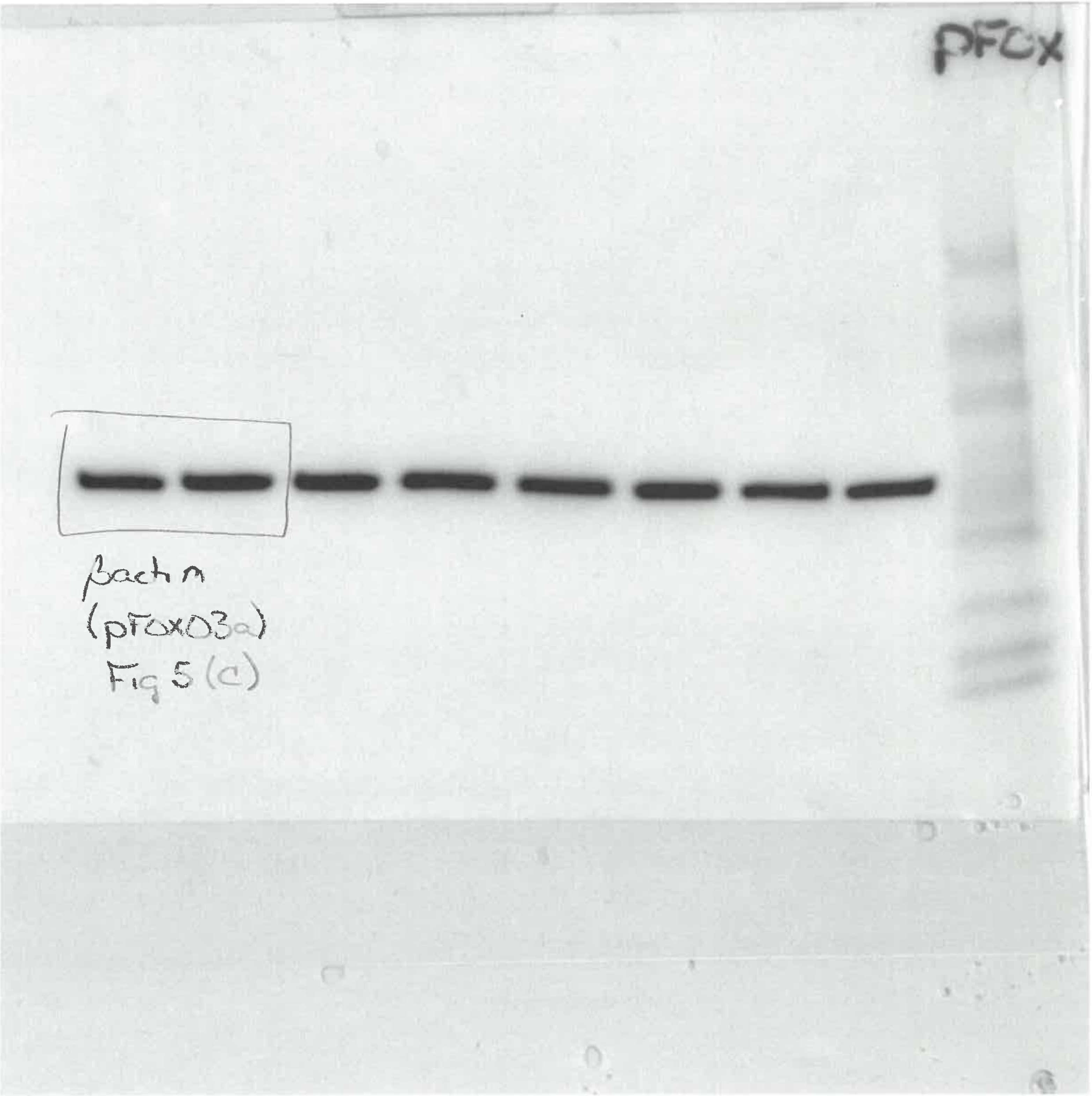
pFOX
βactin
(pFOXO3a)
Fig 5(c)

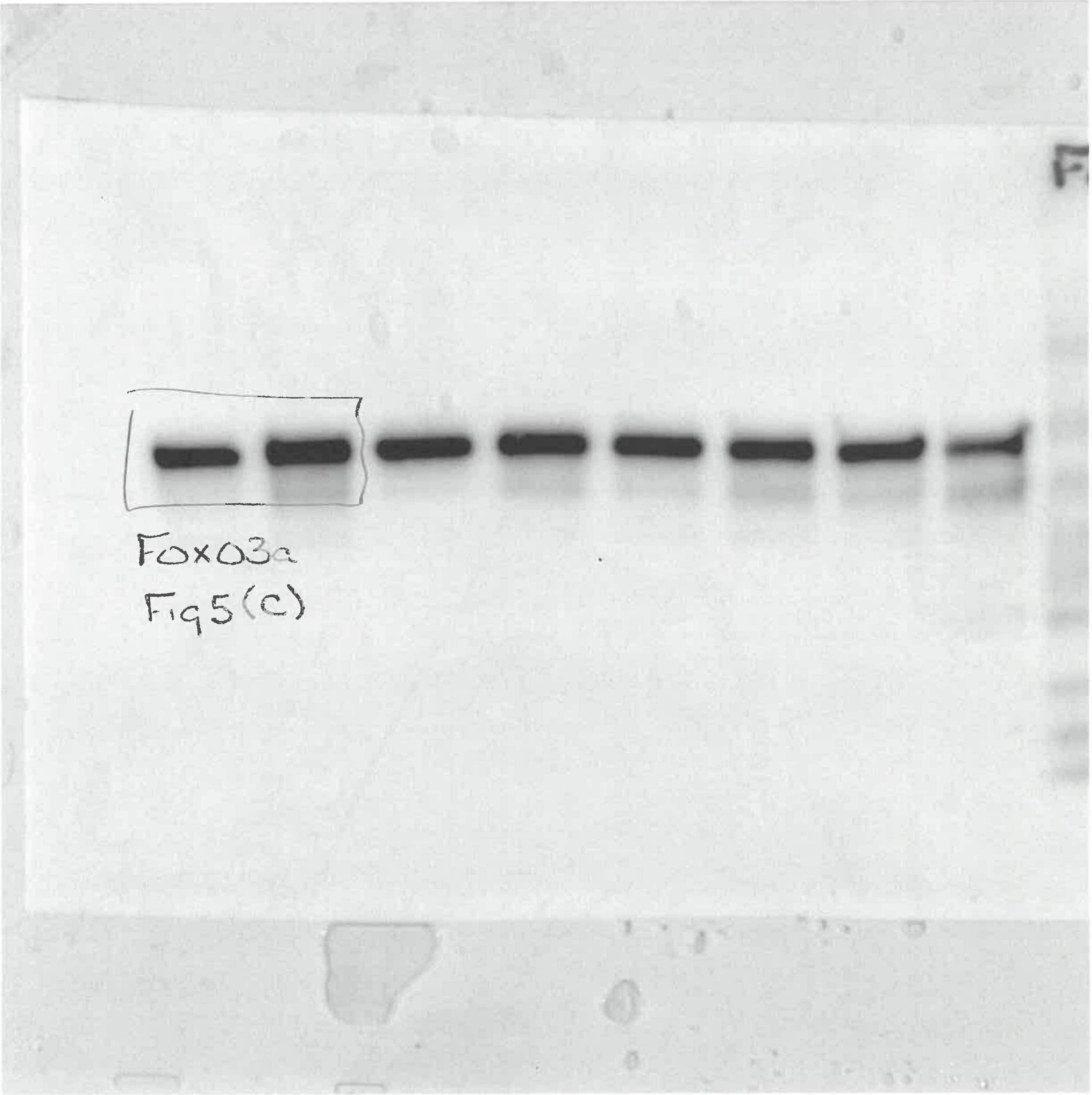
Foxo3a
Fig5(c)

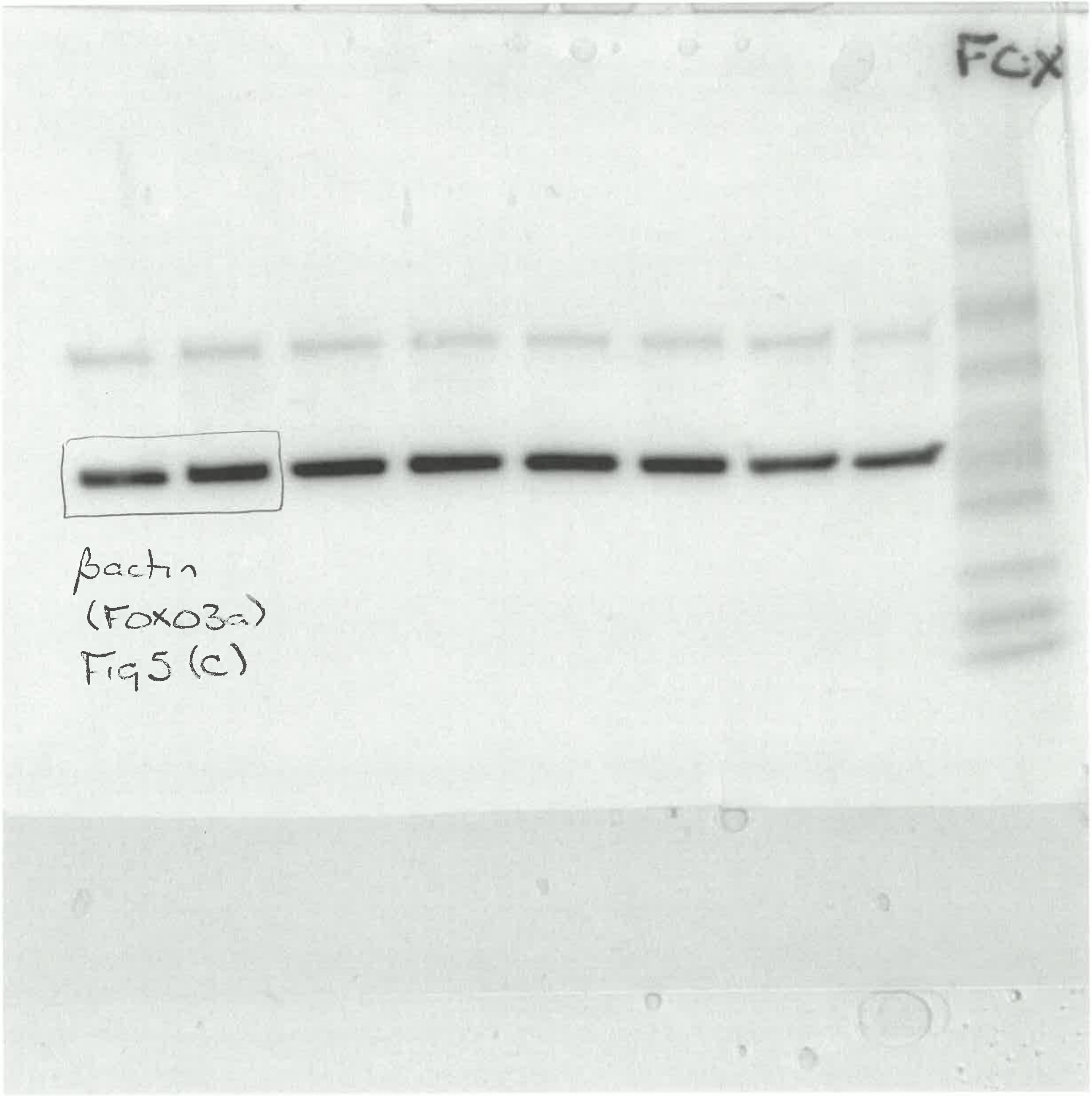
FOX
βactin
(FOXO3a)
Fig5 (C)

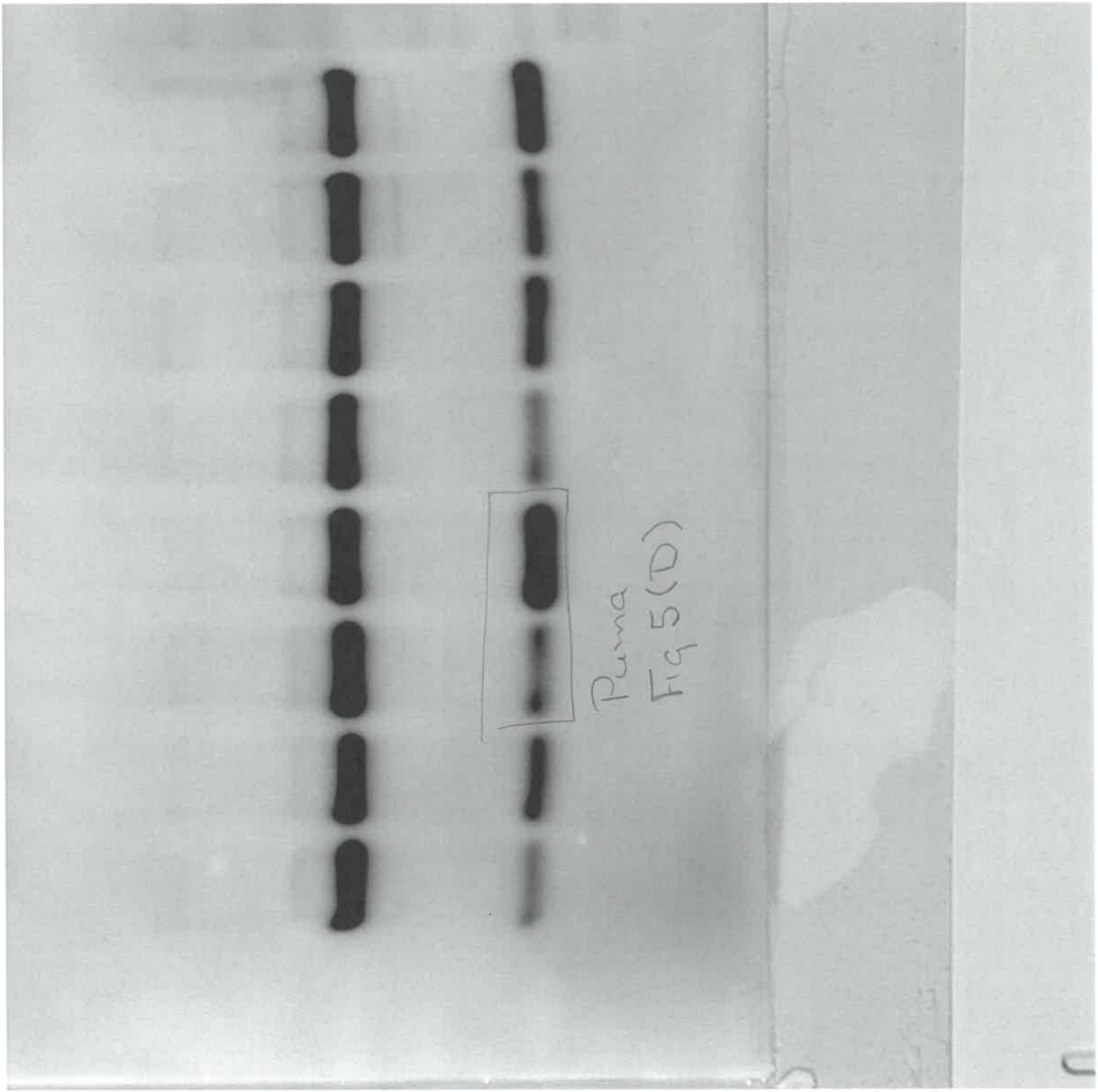
Puma
Fig 5(D)

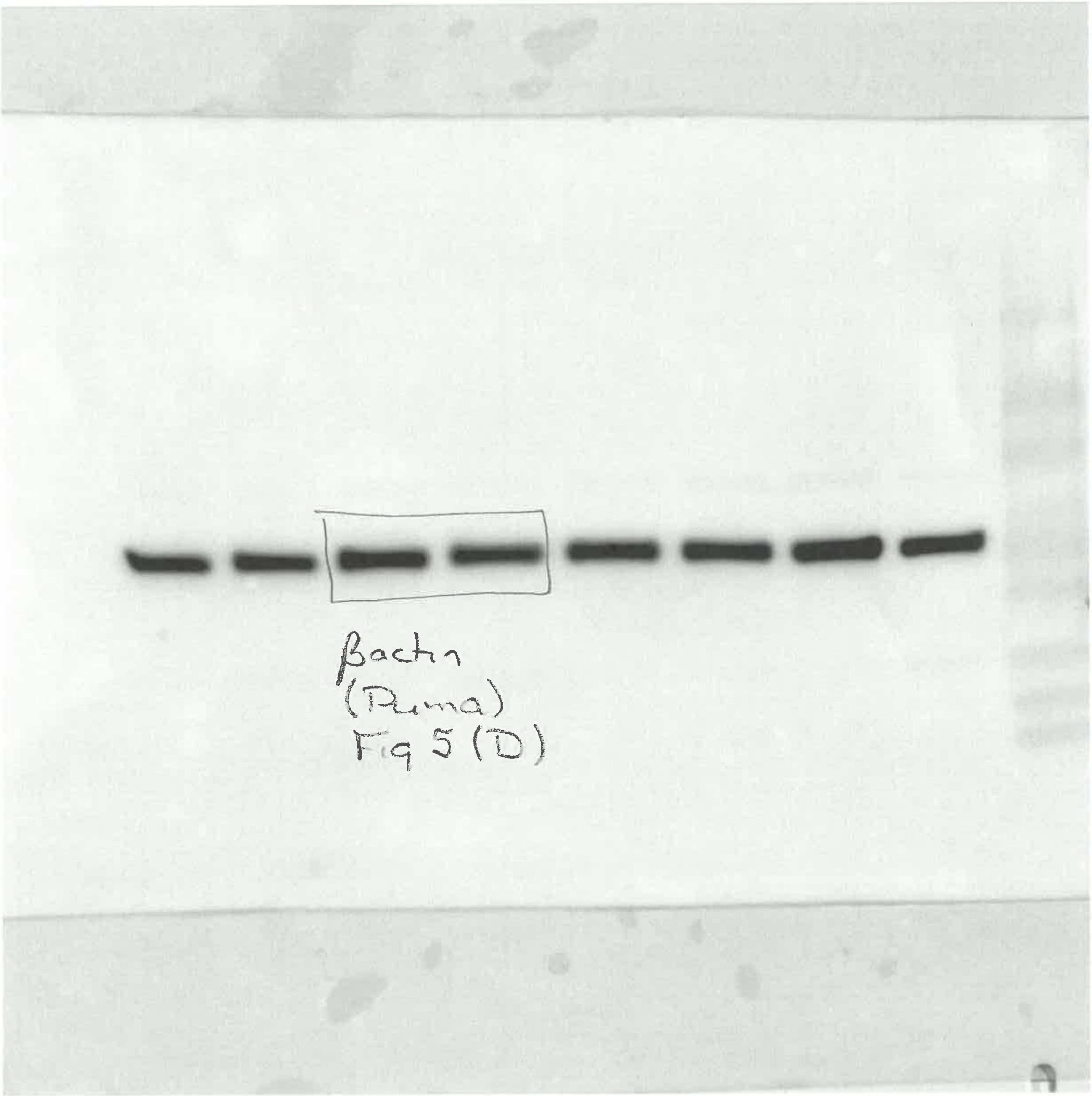
βactin
(Puma)
Fig 5 (D)

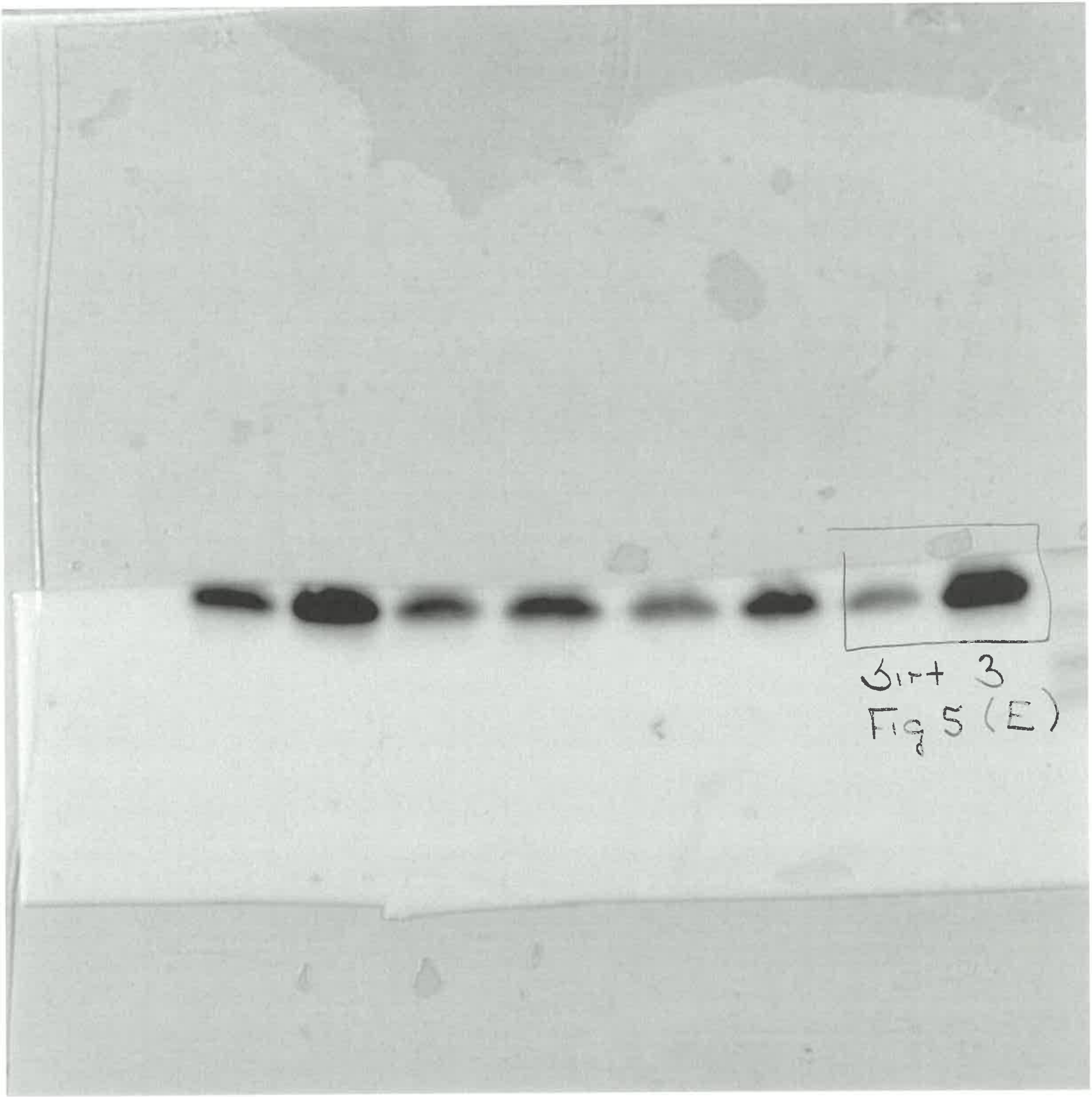
Sirt 3
Fig 5 (E)

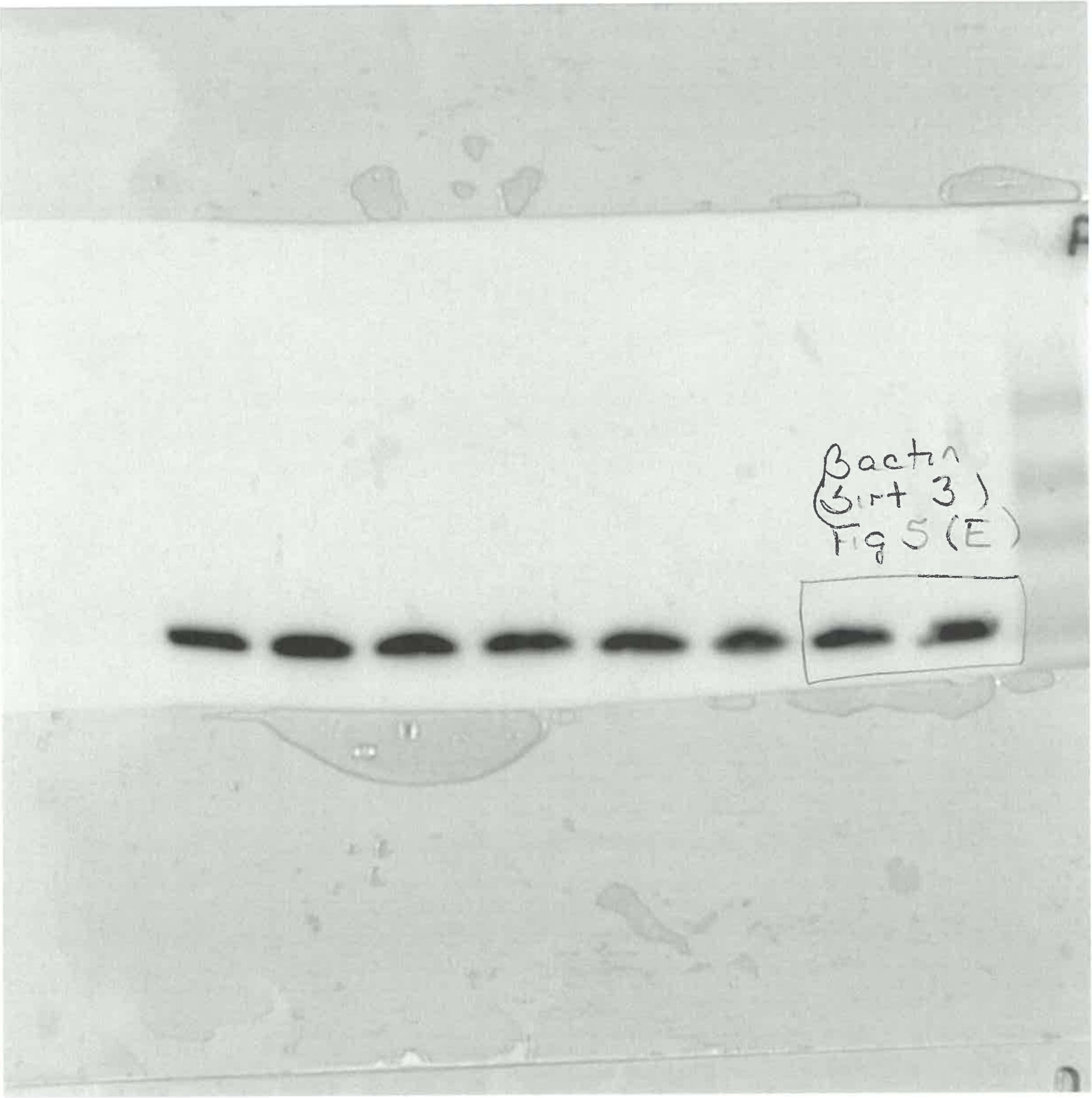
βactin
(Sirt 3)
Fig 5(E)

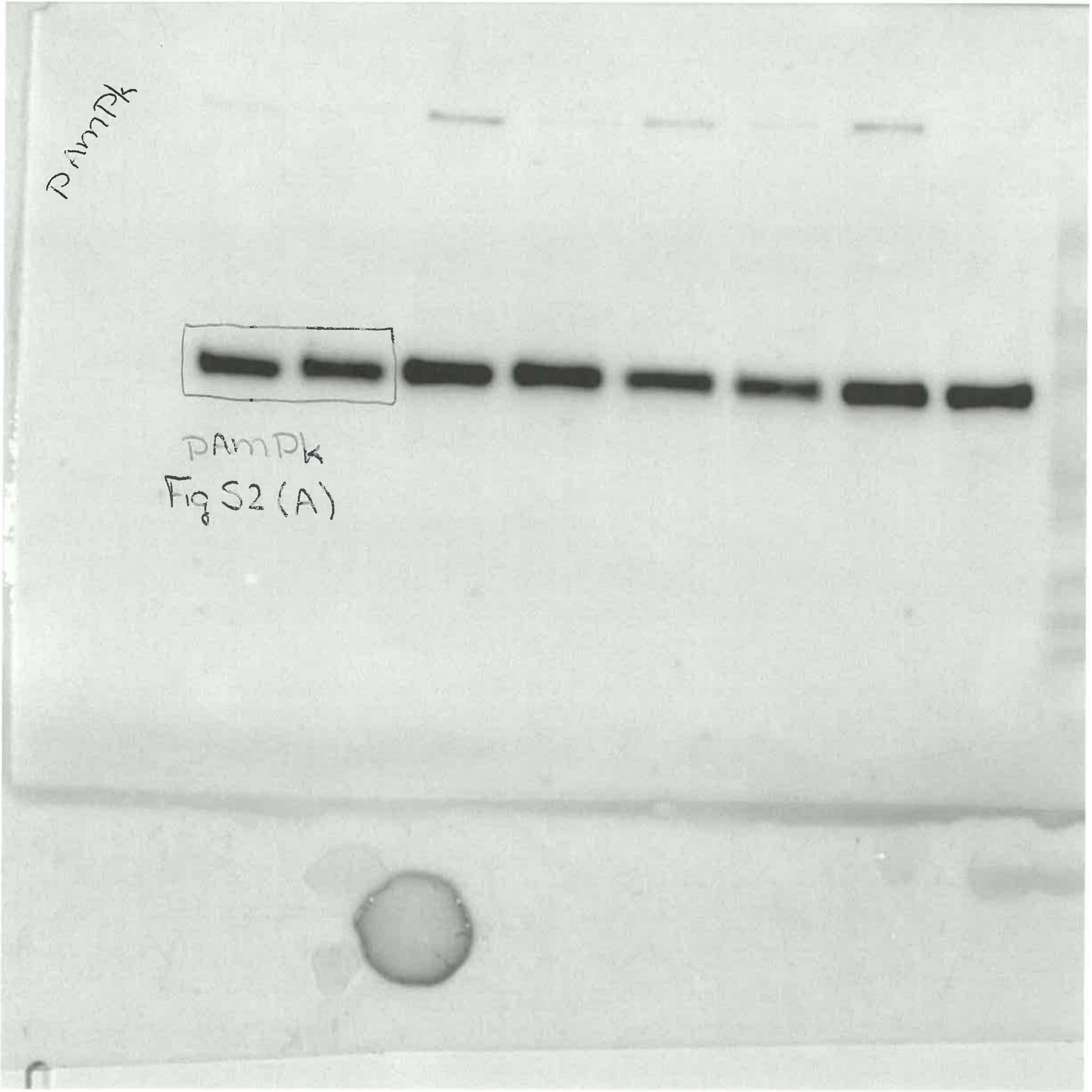
pAmPk
pAmPk
Fig S2 (A)

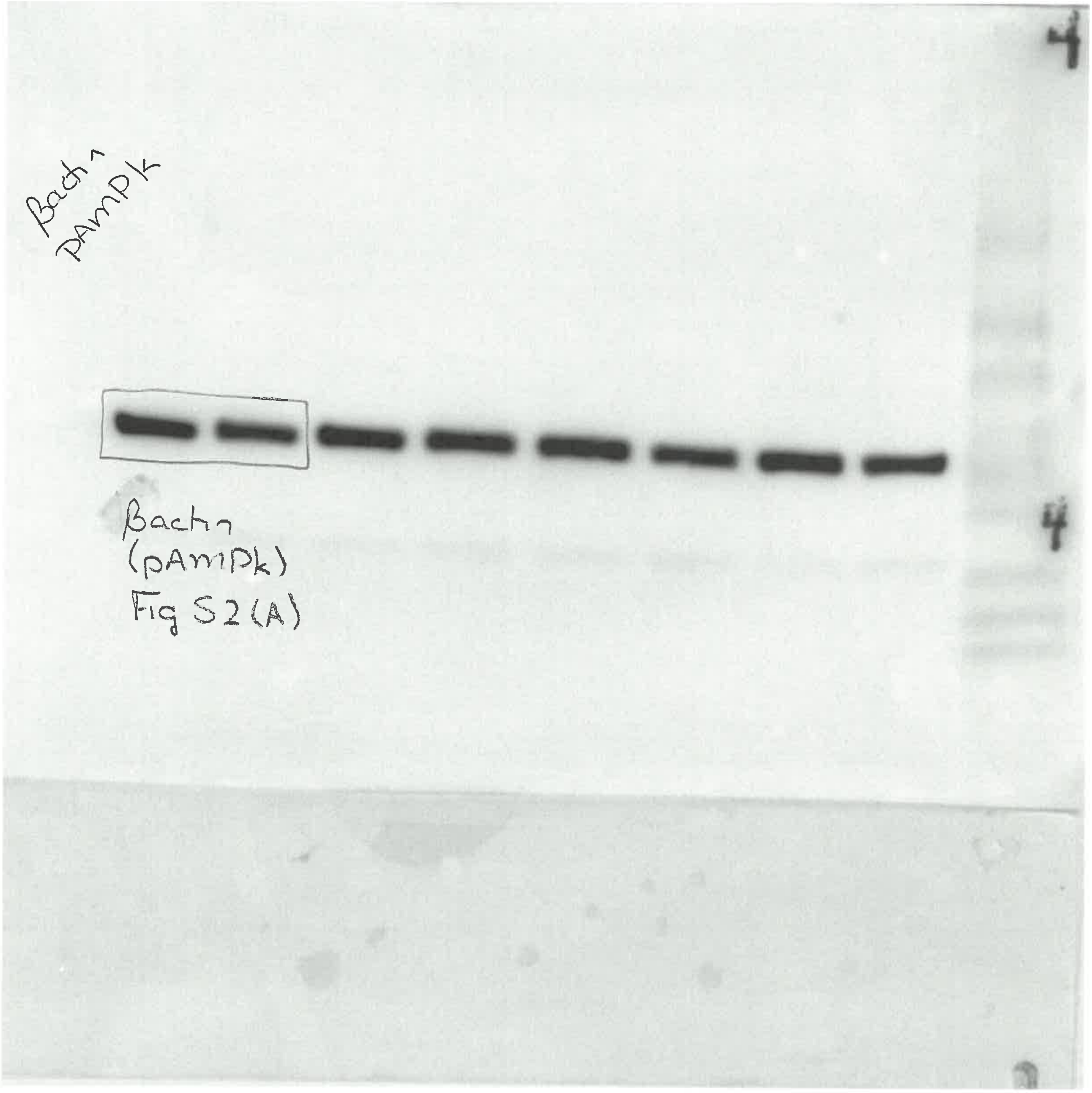
Bach1
pAMPK
Bach1
(pAMPK)
Fig S2(A)

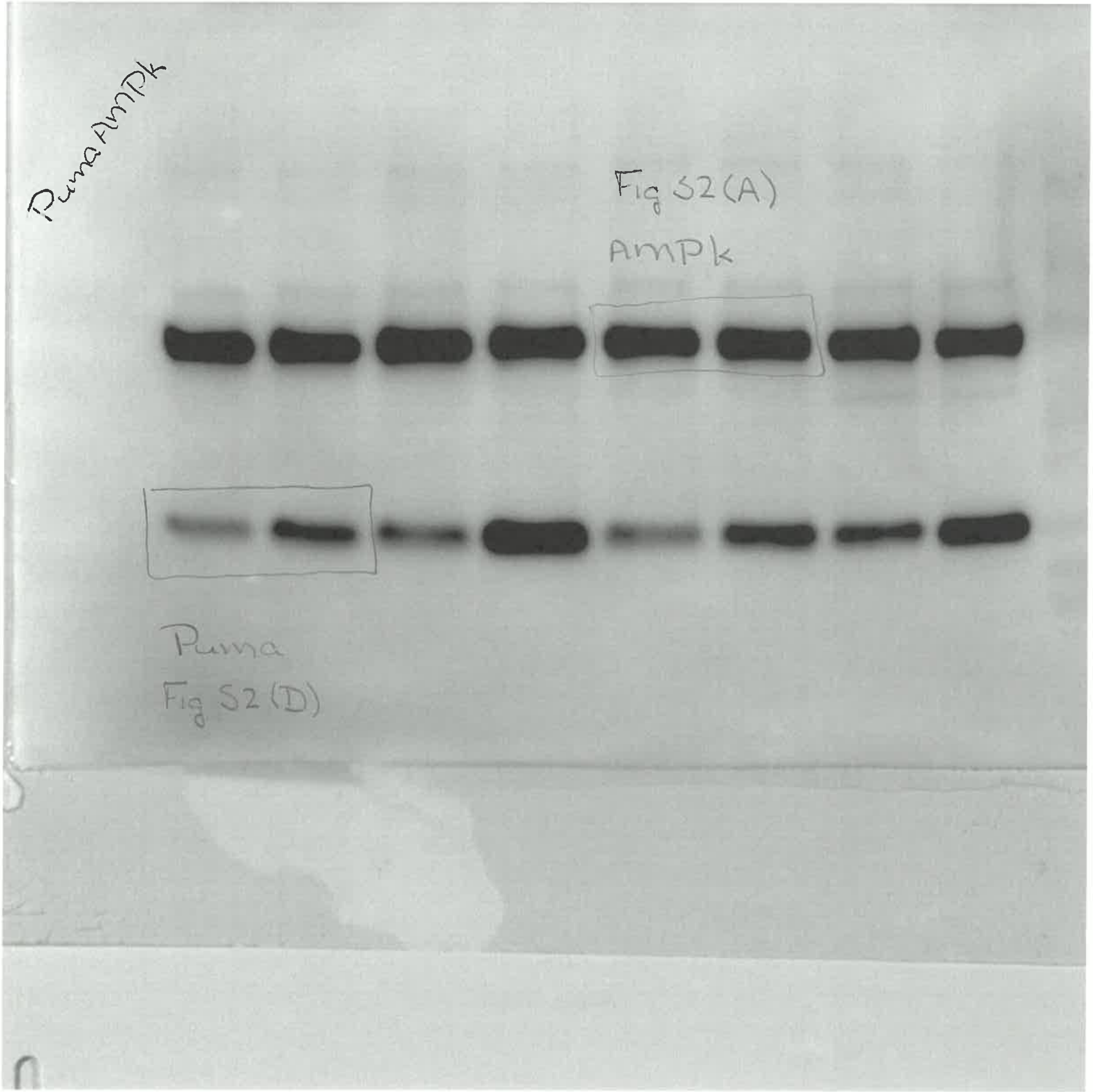
Puma AMPk
Fig S2 (A)
AMPk
Puma
Fig S2 (D)

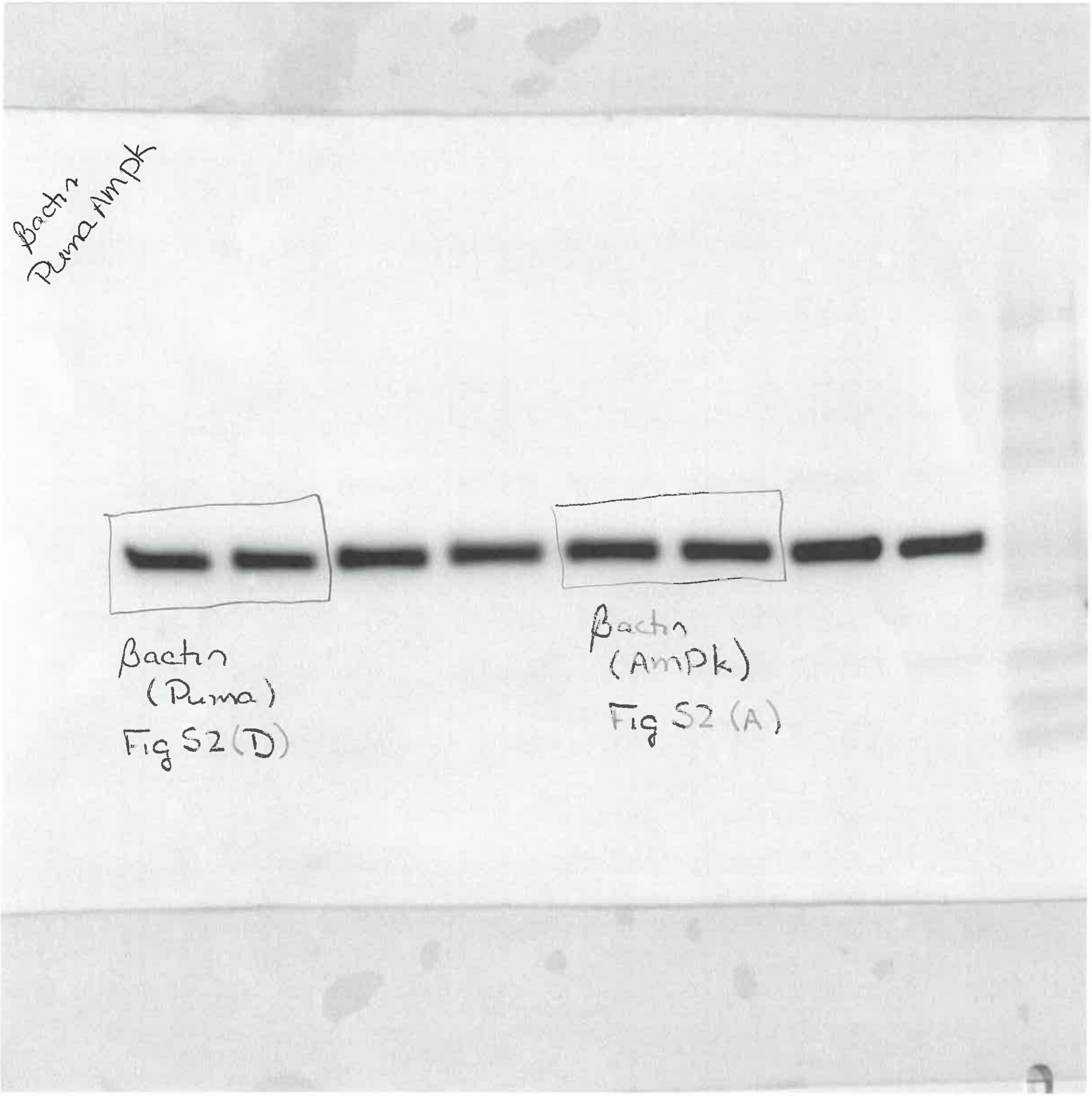
βactin
Puma AMPK
βactin
(Puma)
Fig S2 (D)
βactin
(AMPK)
Fig S2 (A)

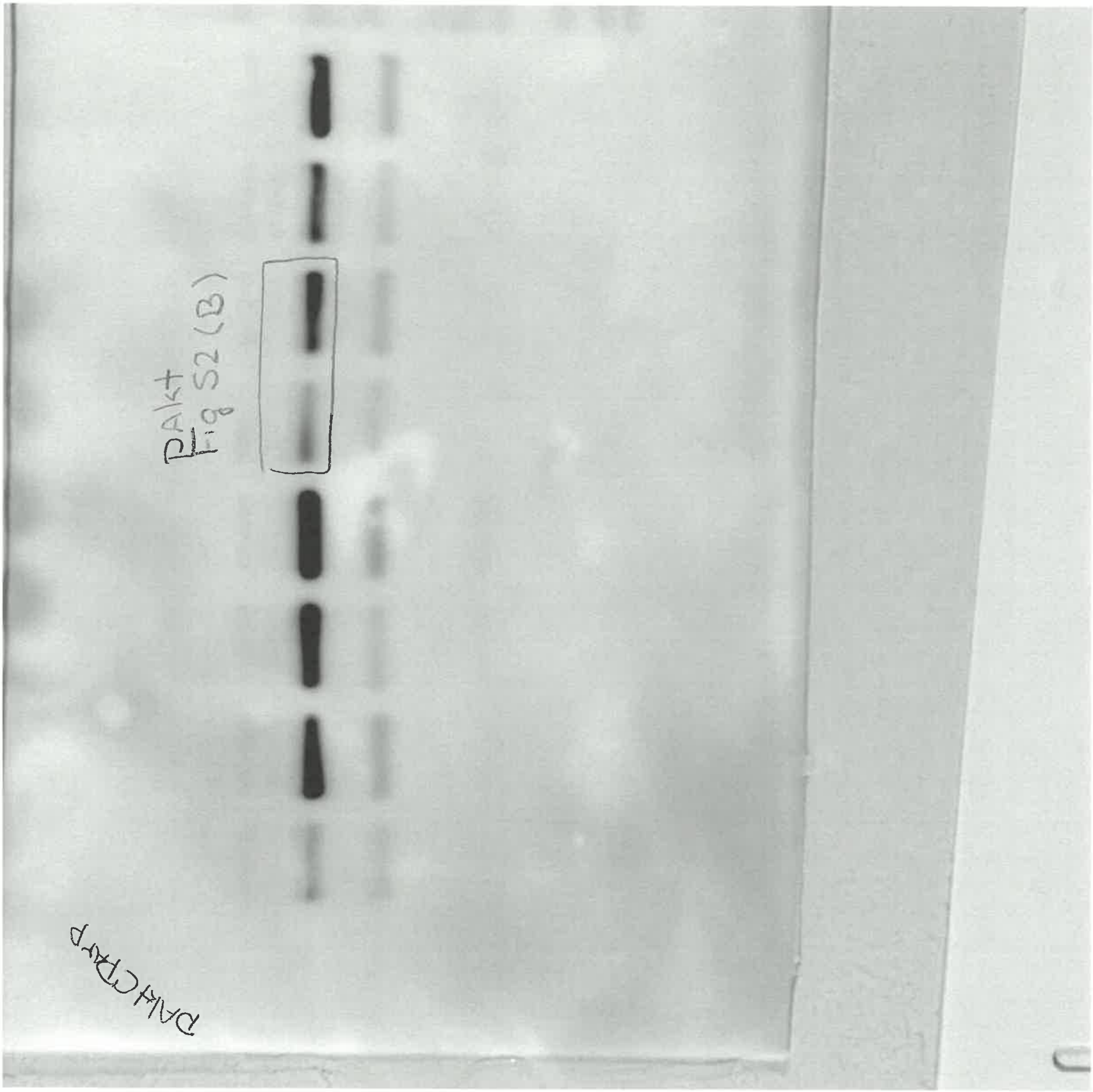
PAkt
Fig S2 (B)

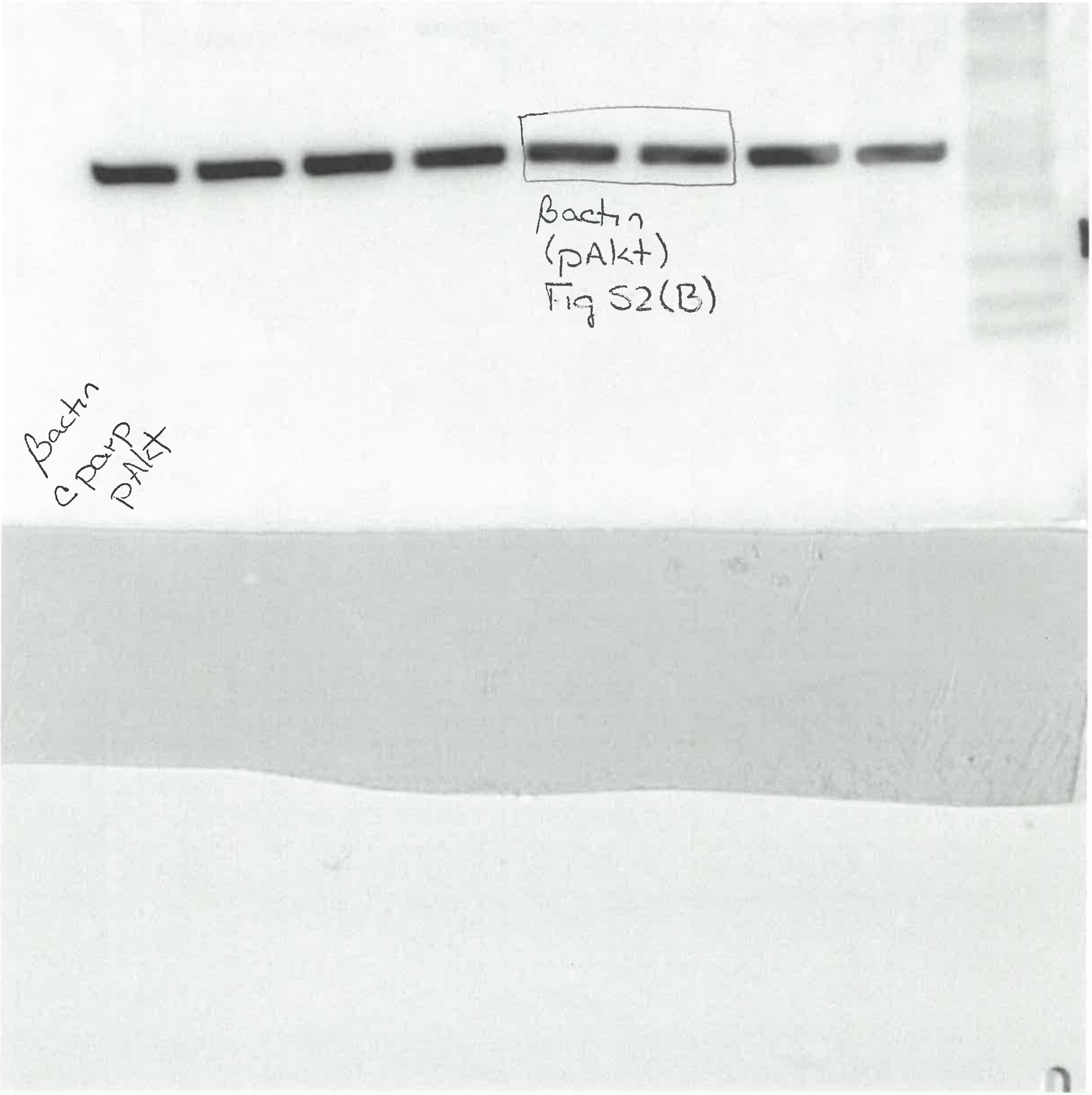
Bactin
(pAkt)
Fig S2(B)
Bactin
c parp
pAkt

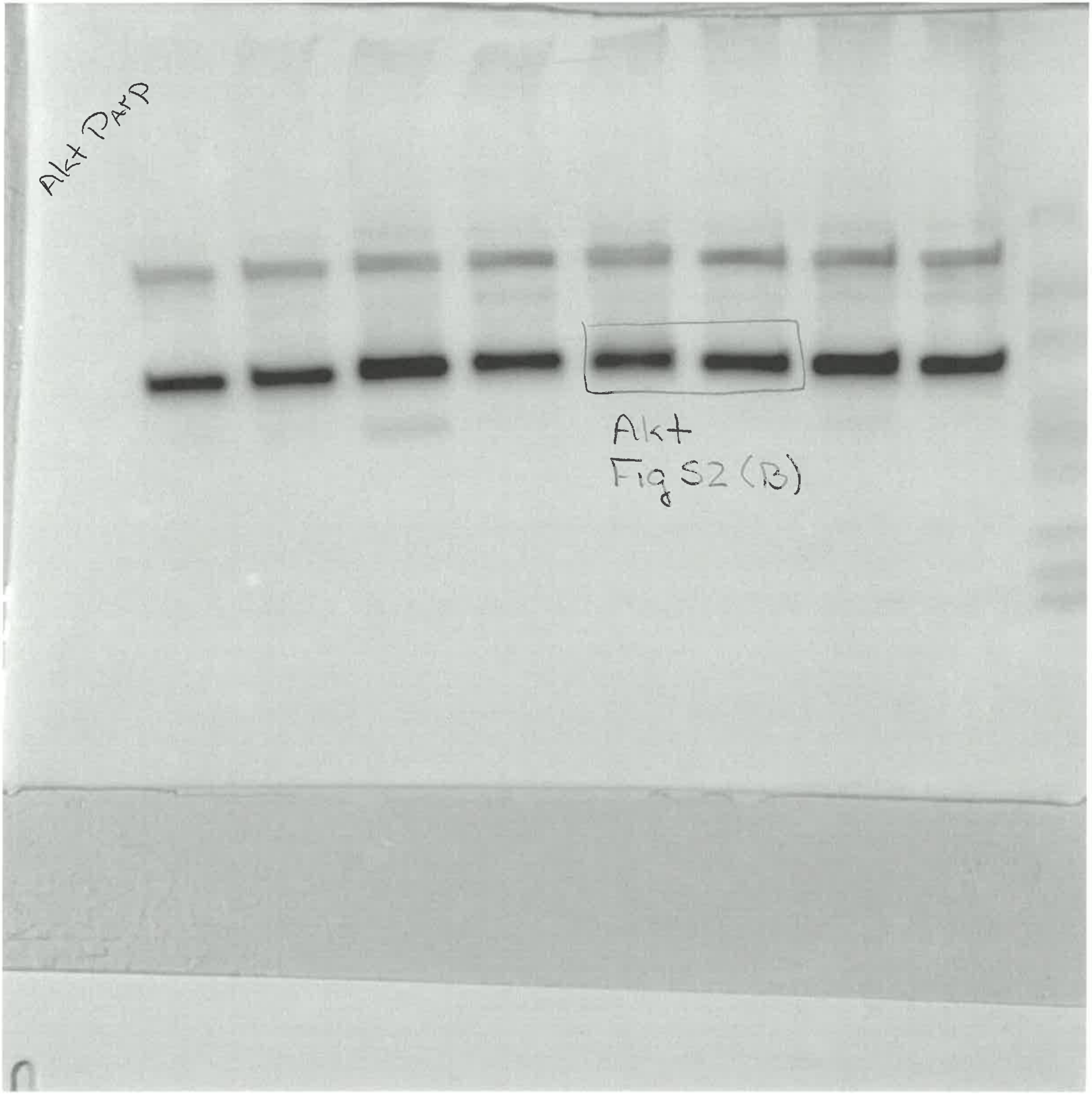
Akt Parp
Akt
Fig S2 (B)

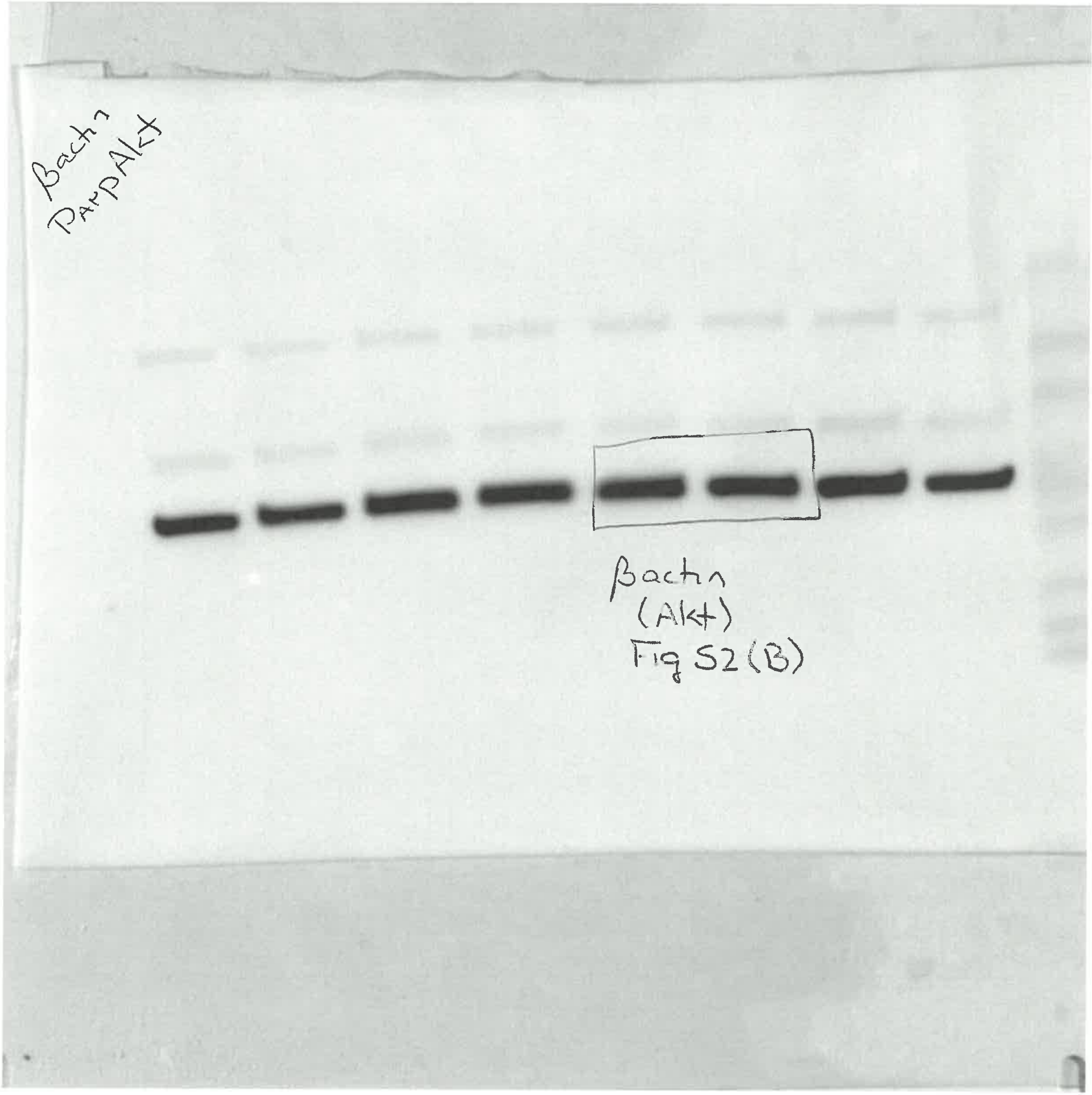
βactin
ParpAlkt
βactin
(Akt)
Fig S2(B)

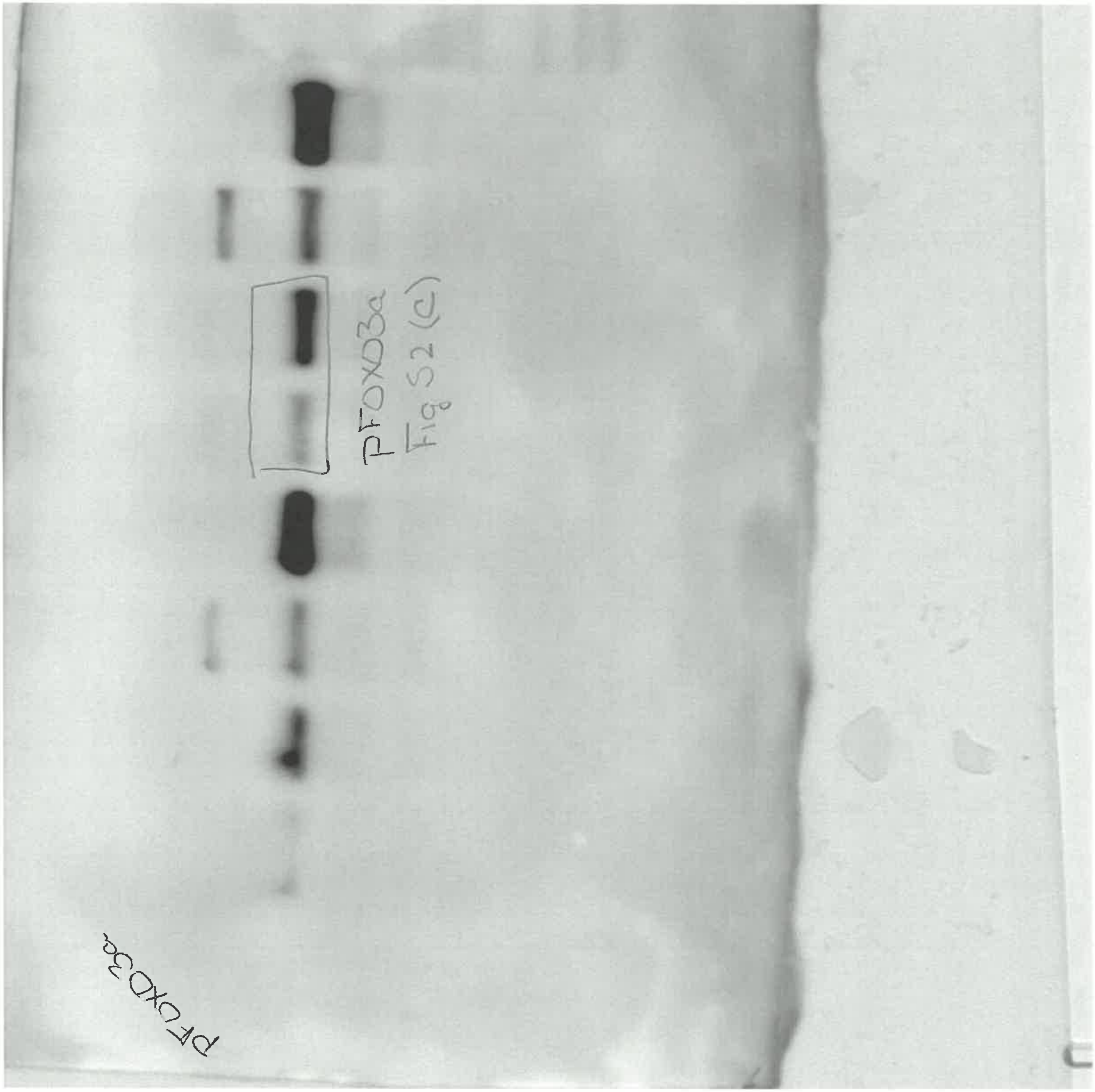
pFOXO3a
Fig S2 (c)
pFOXO3a

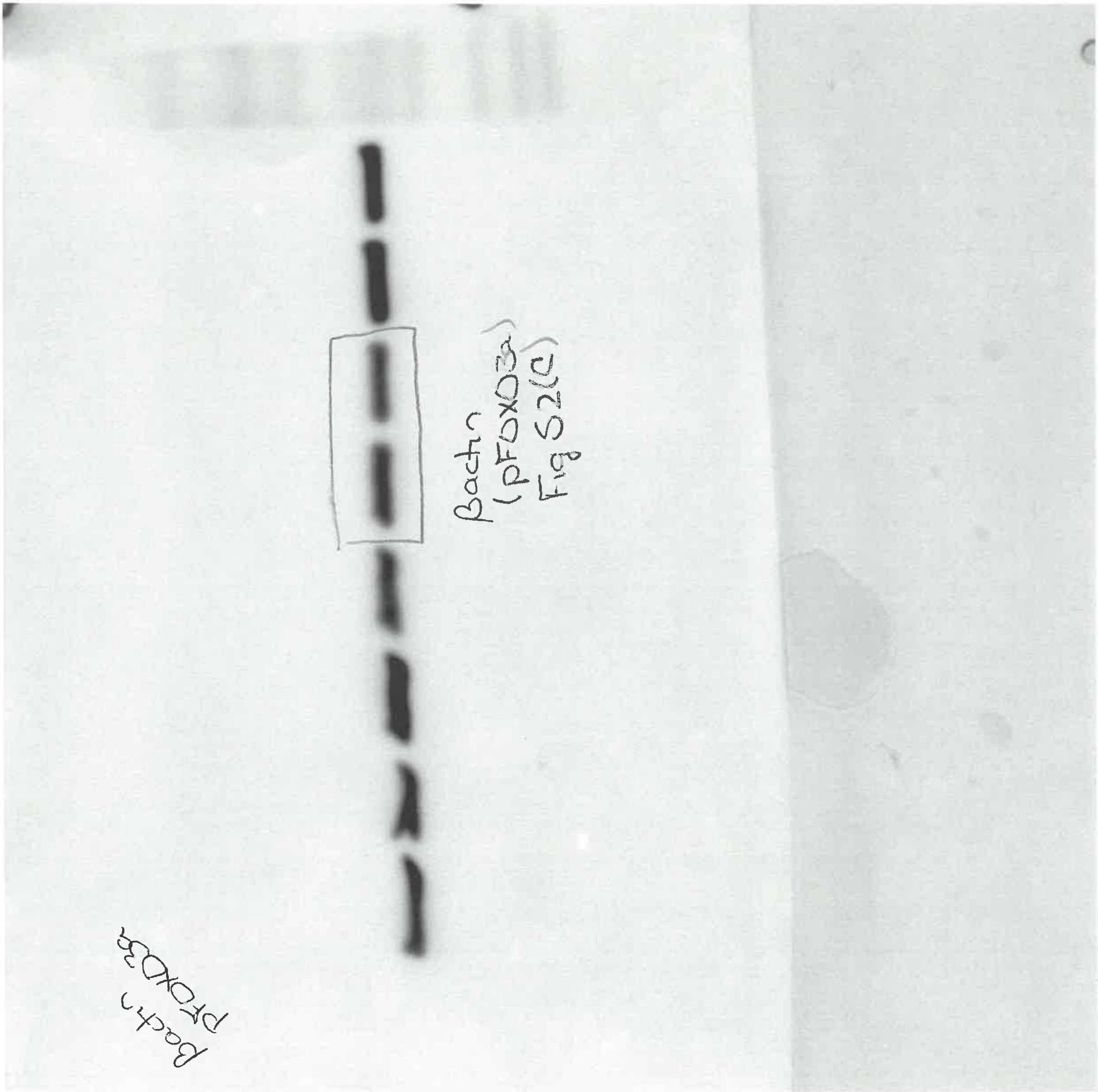
Bactin
(pFOXO3a)
Fig S2(C
Bactin
pFOXO3

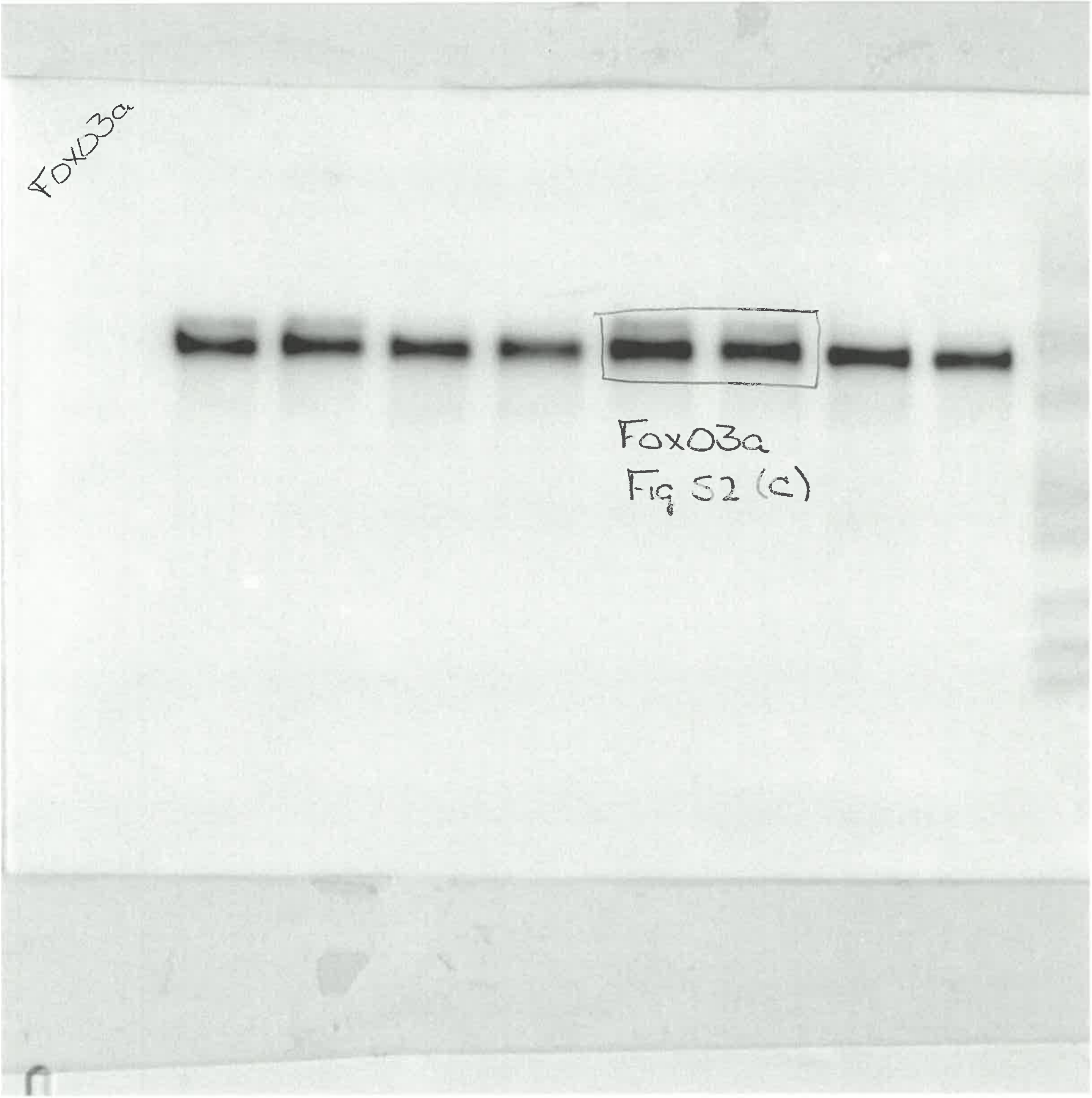
FoxO3a
FoxO3a
Fig S2 (c)

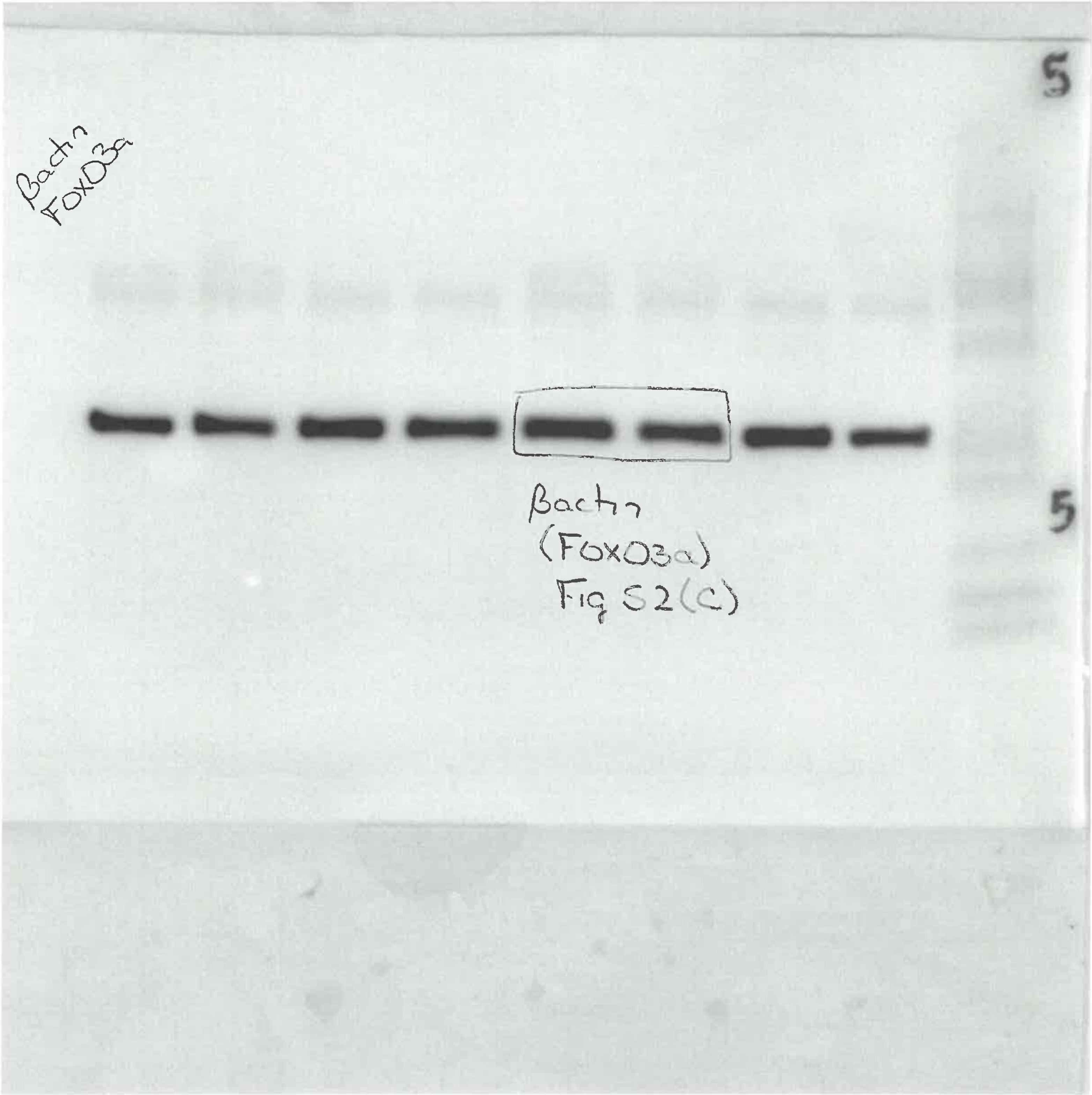
βactin FoxO3a
5
βactin (FoxO3a) Fig S2(c)
5

Sirt 3

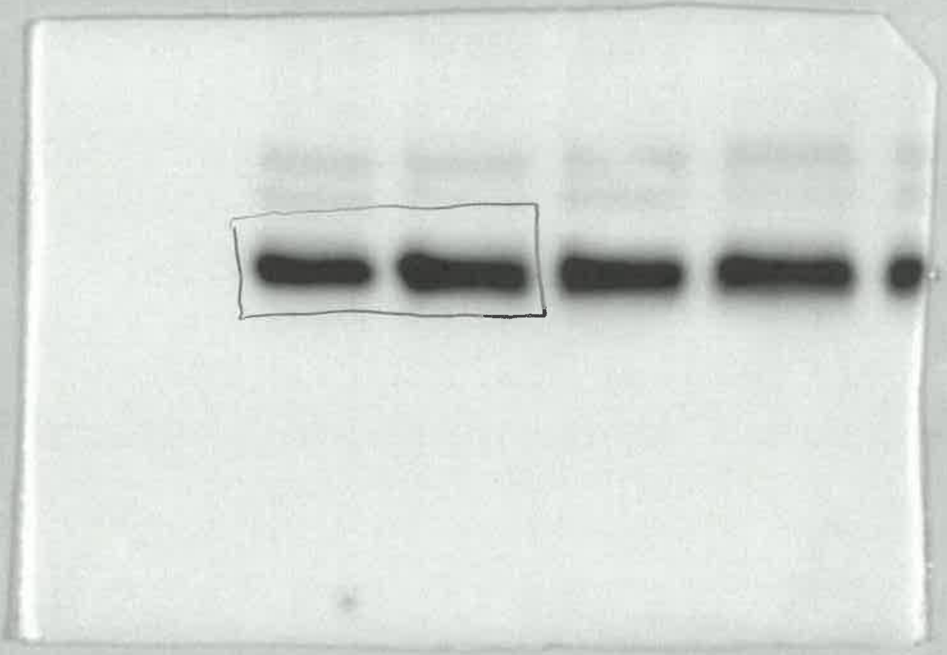

Fig S2 (E)

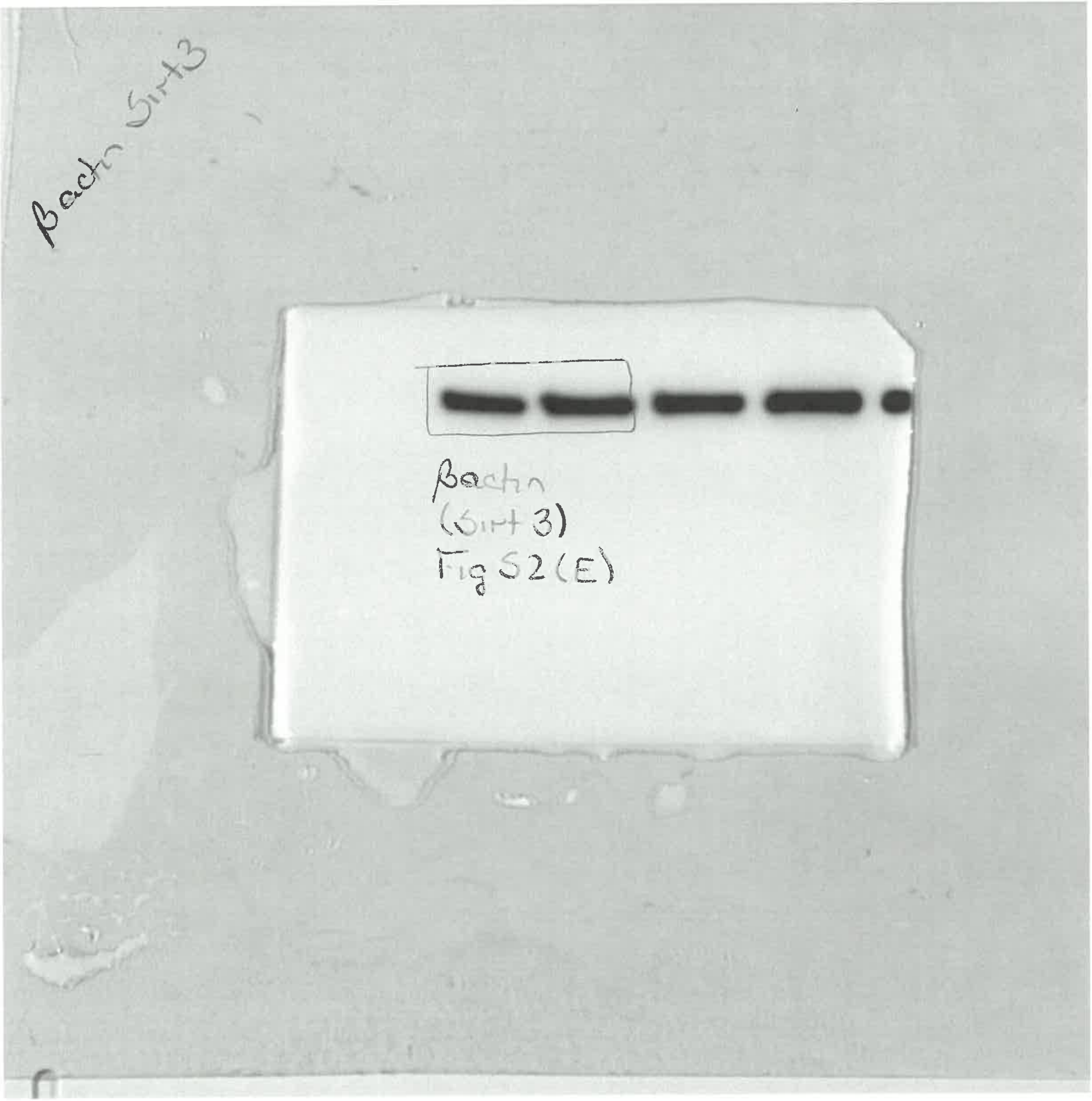
βactn Sirt3
βactn
(Sirt 3)
Fig S2(E)

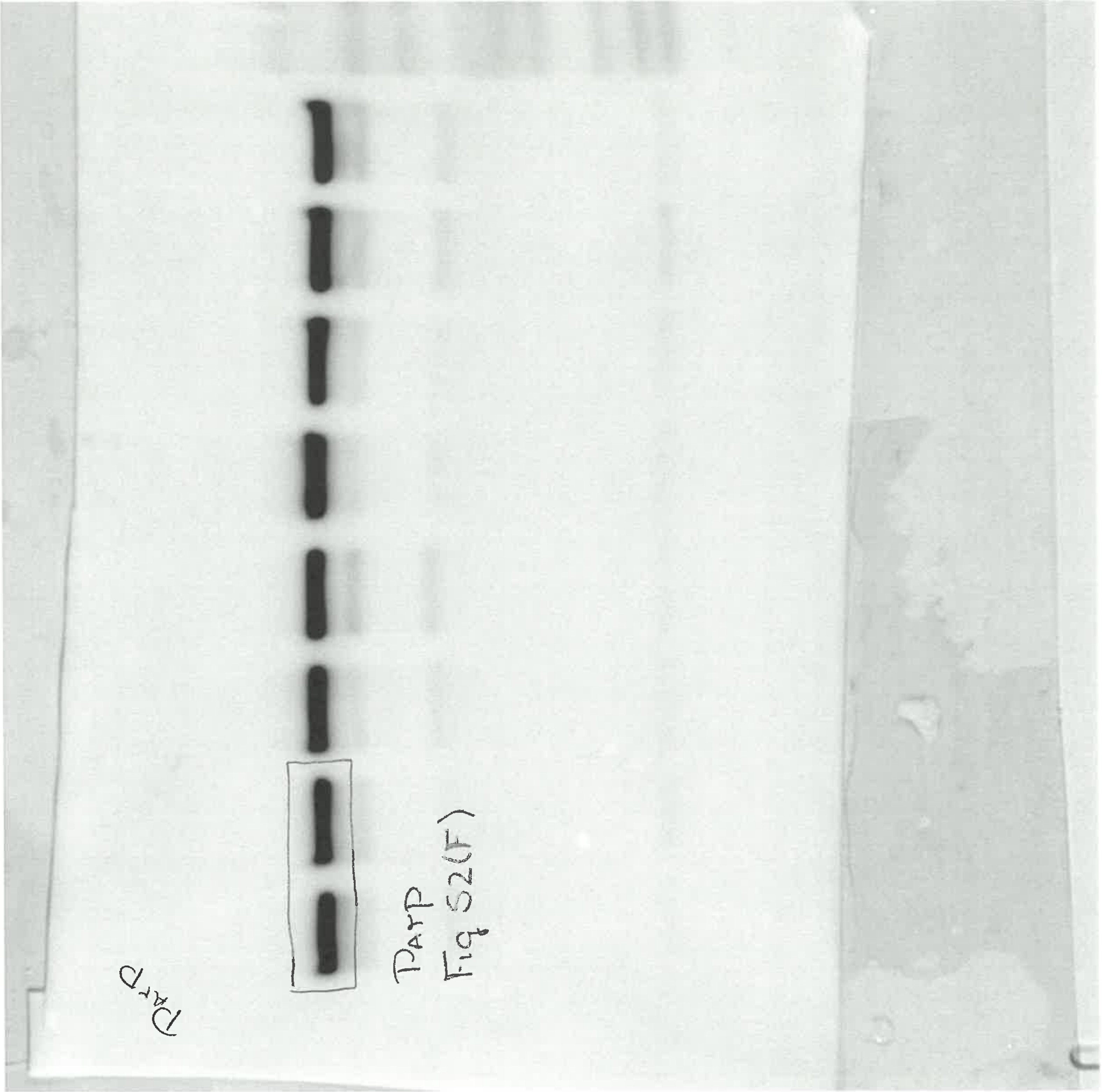
Parp
Parp
Fig S2(F)

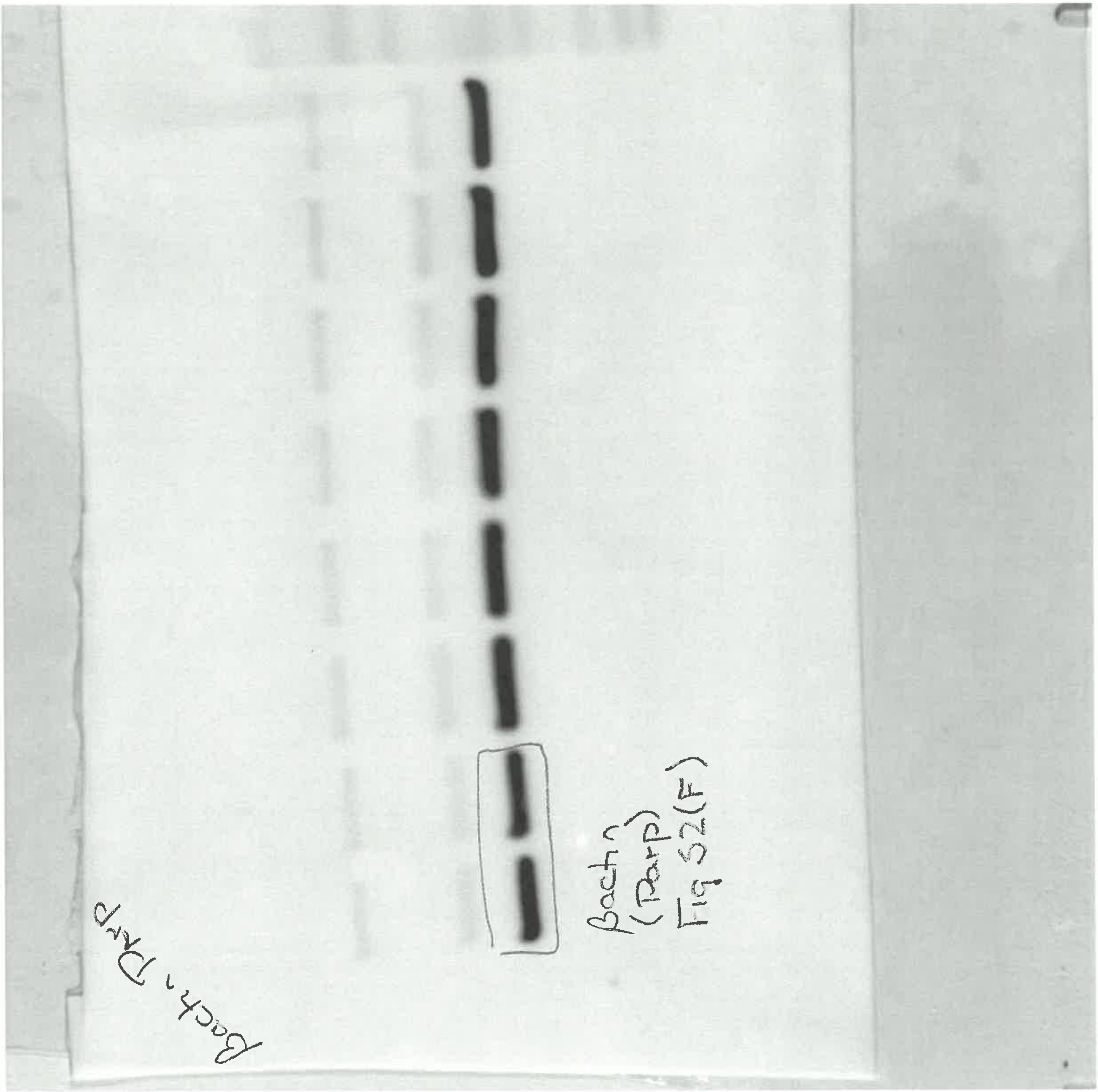
Bach1
(Parp)
Fig S2(F)
Bach1, Parp

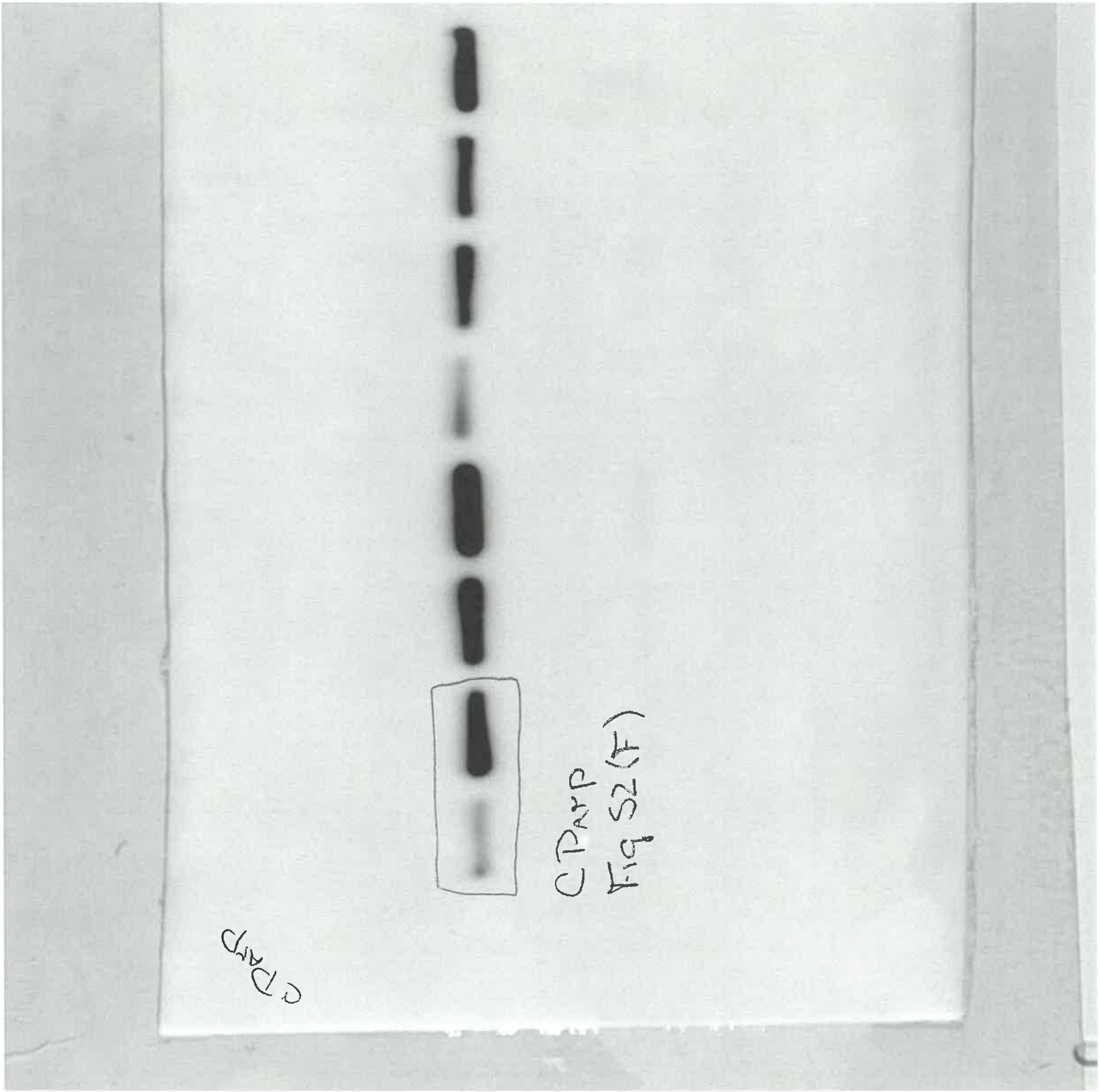
C Parp
Fig S2 (F)
C Parp

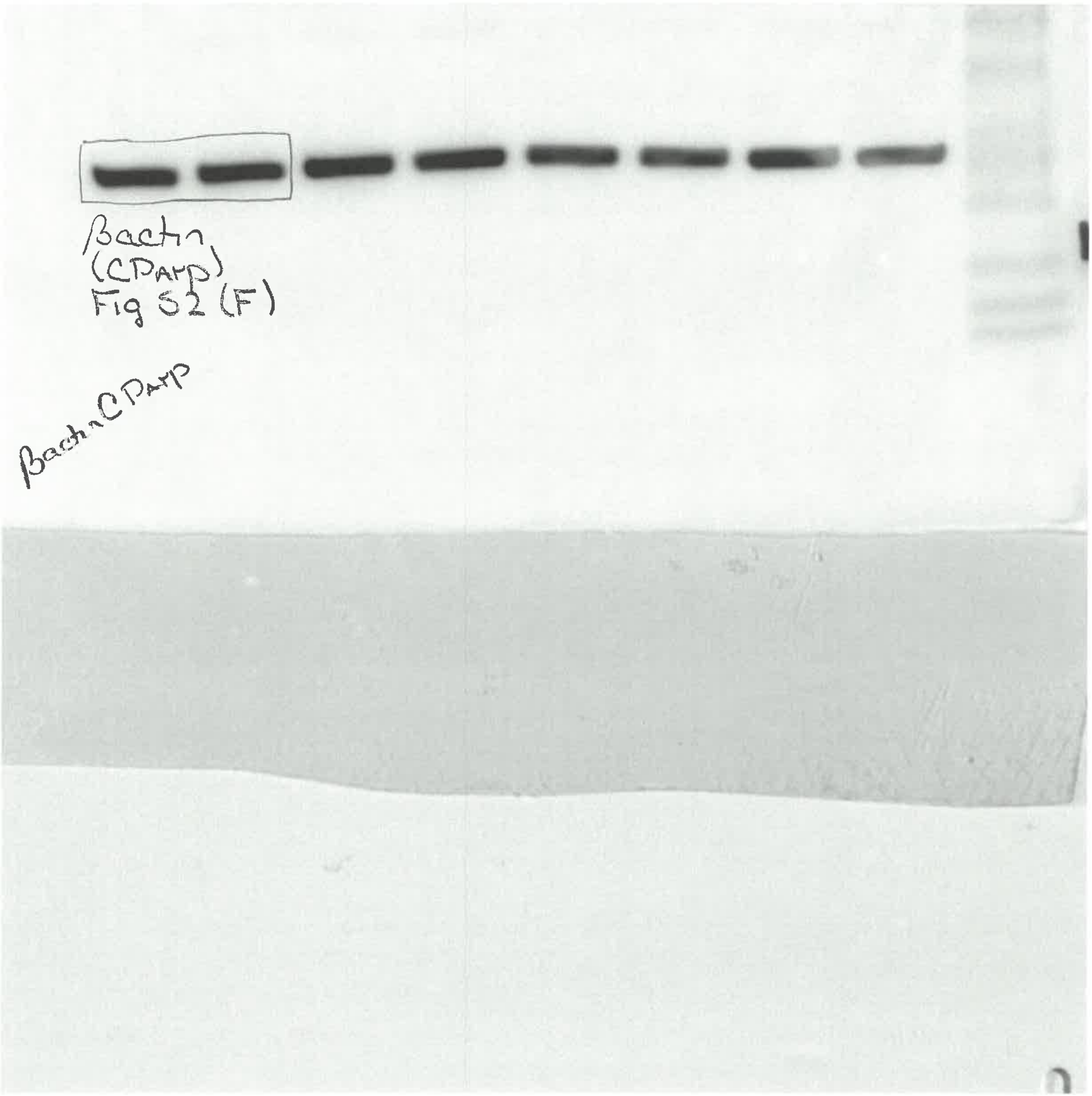
βactin
(CParp)
Fig S2 (F)
βactin CParp

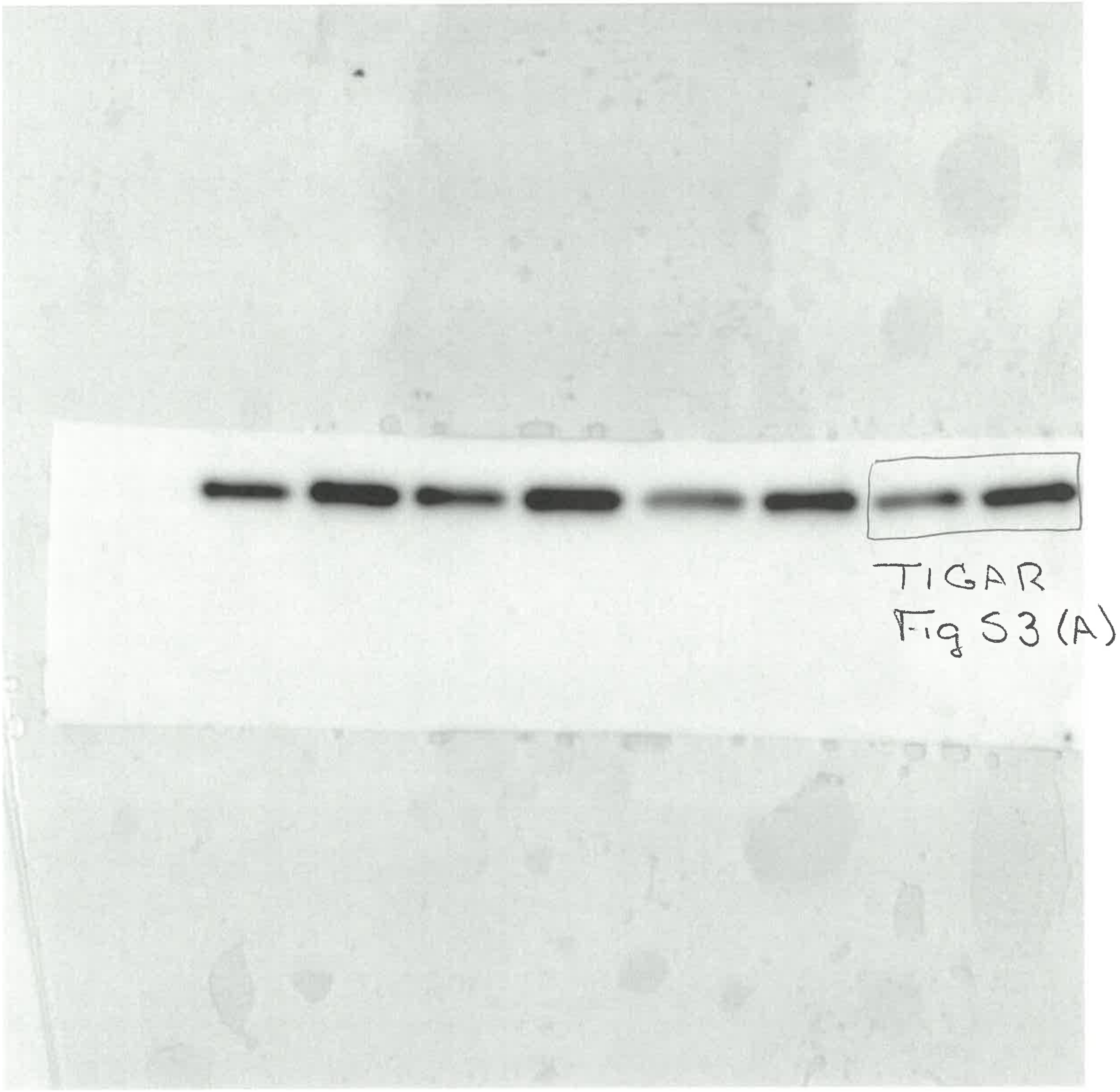
TIGAR
Fig S3 (A)

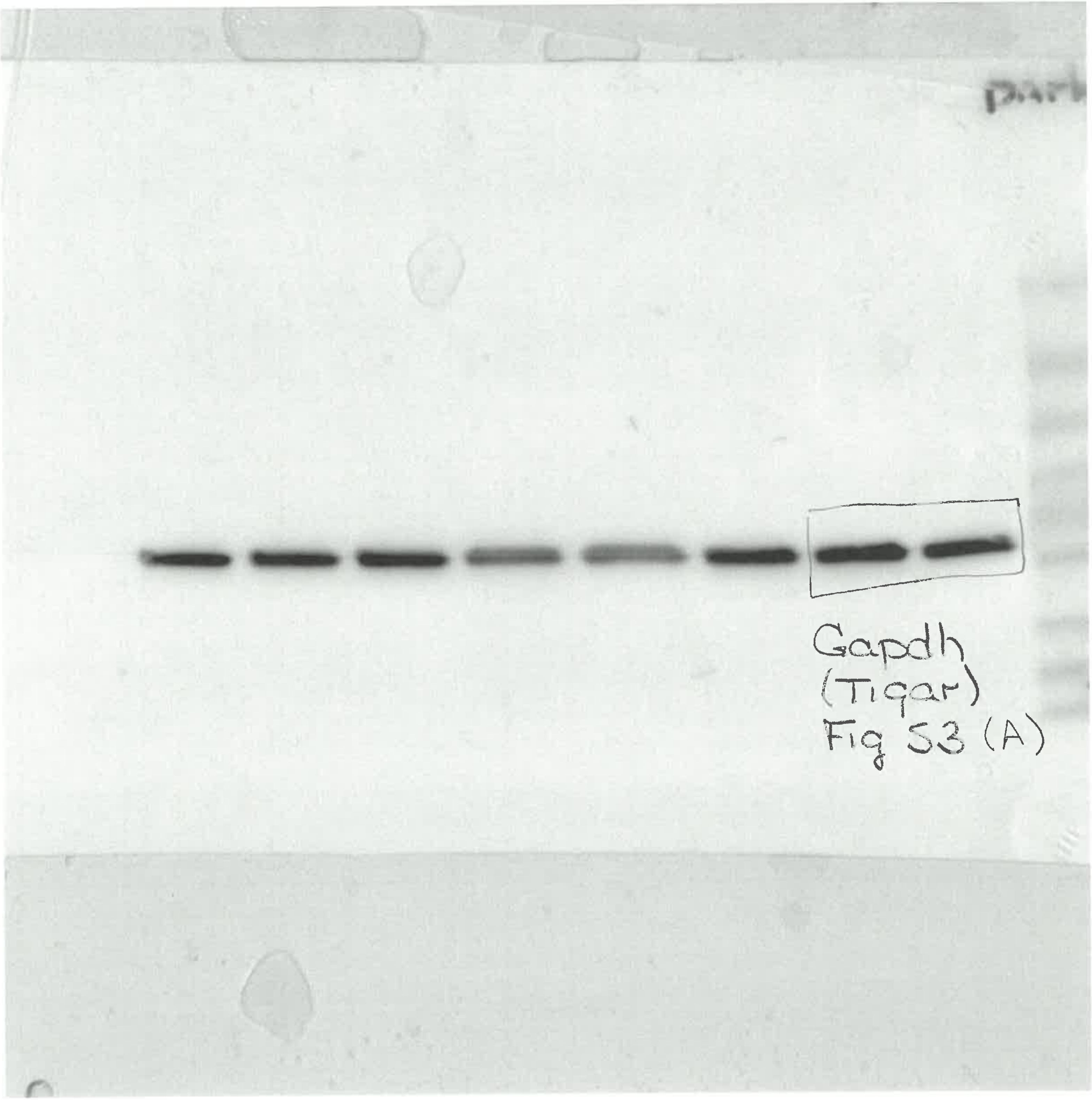
Gapdh
(Tigar)
Fig S3 (A)

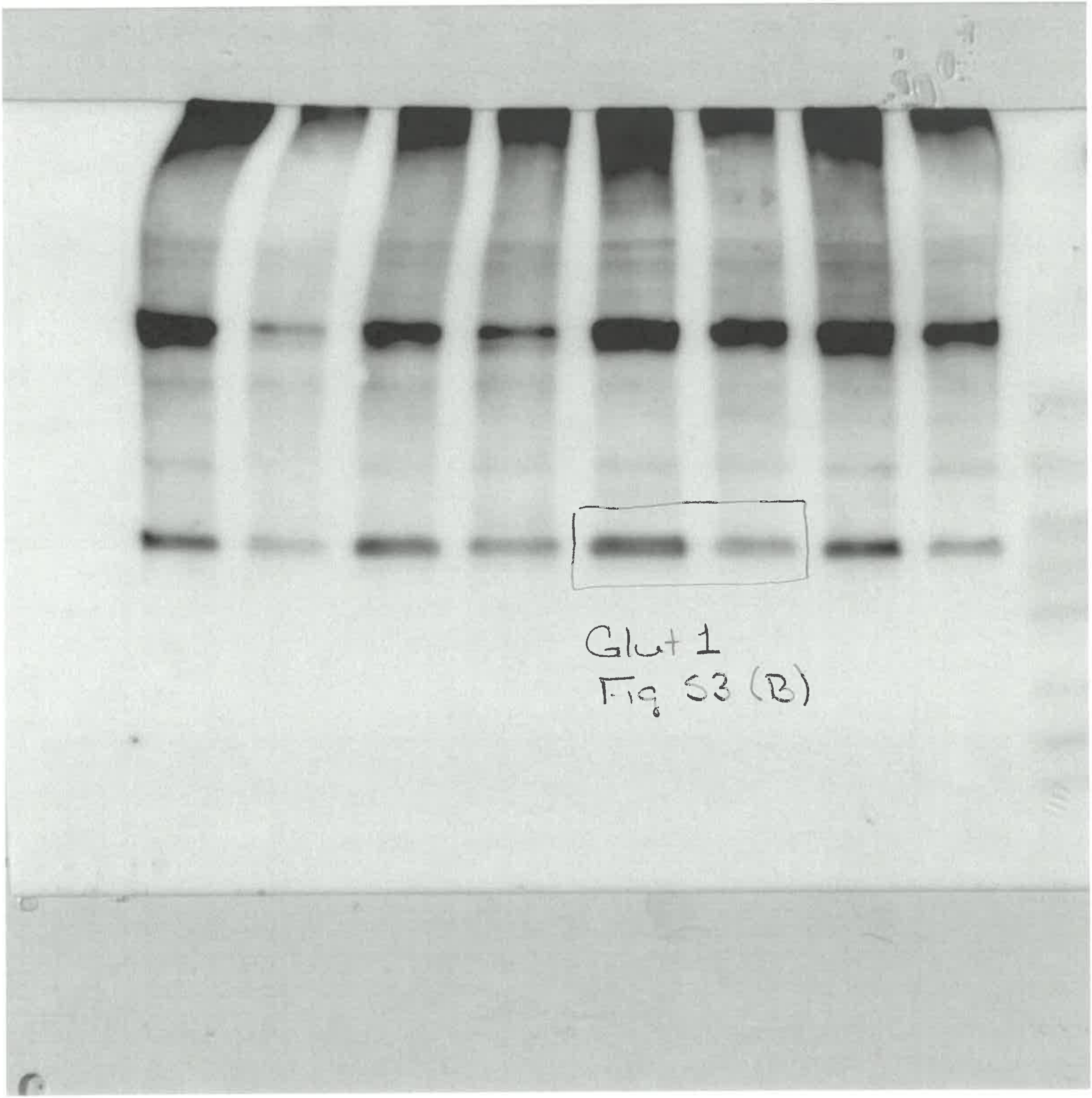
Glut 1
Fig S3 (B)

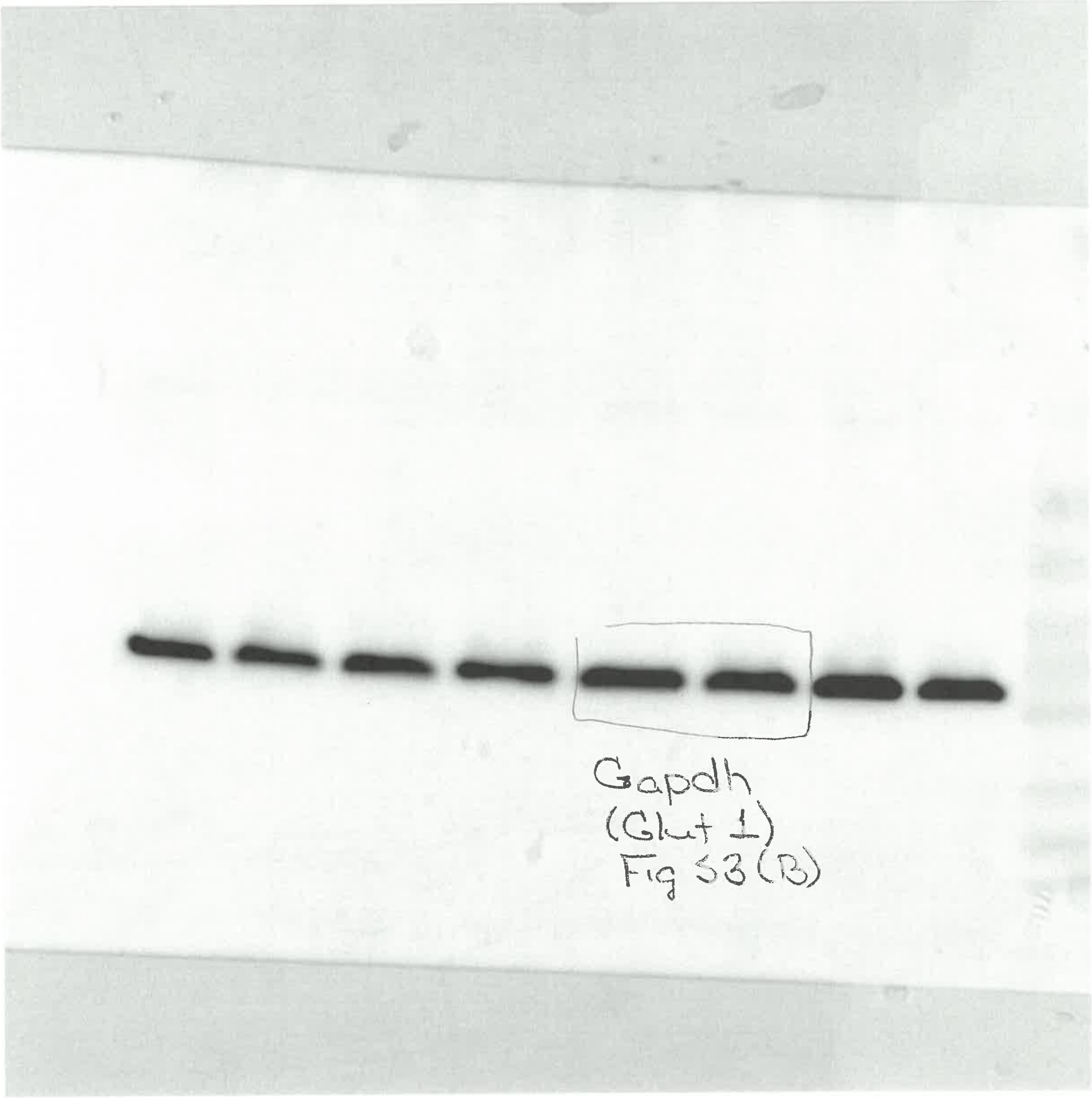
Gapdh
(Glut 1)
Fig S3 (B)

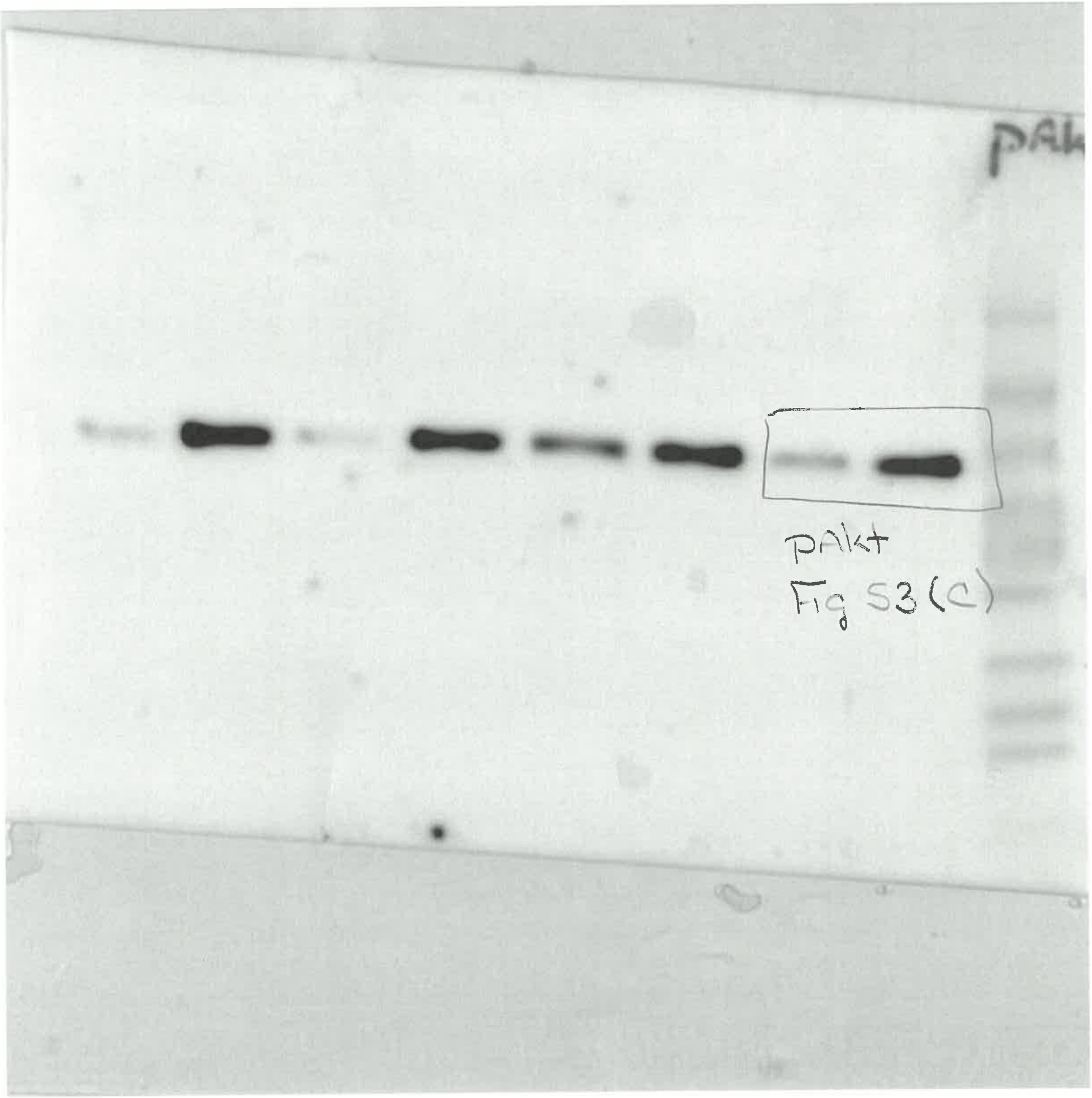
pAkt
Fig S3(c)

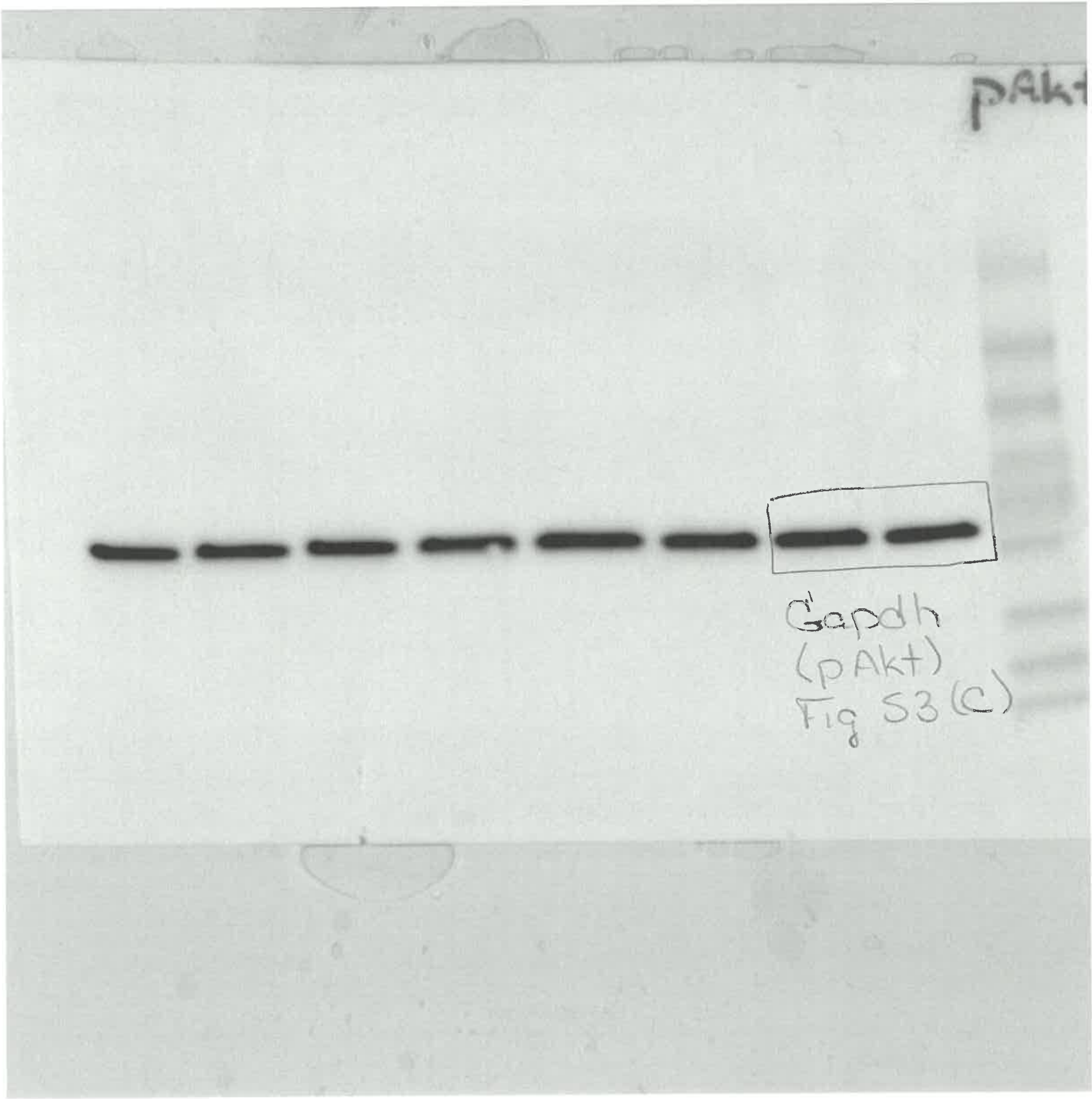
pAkt
Gapdh
(pAkt)
Fig S3 (c)

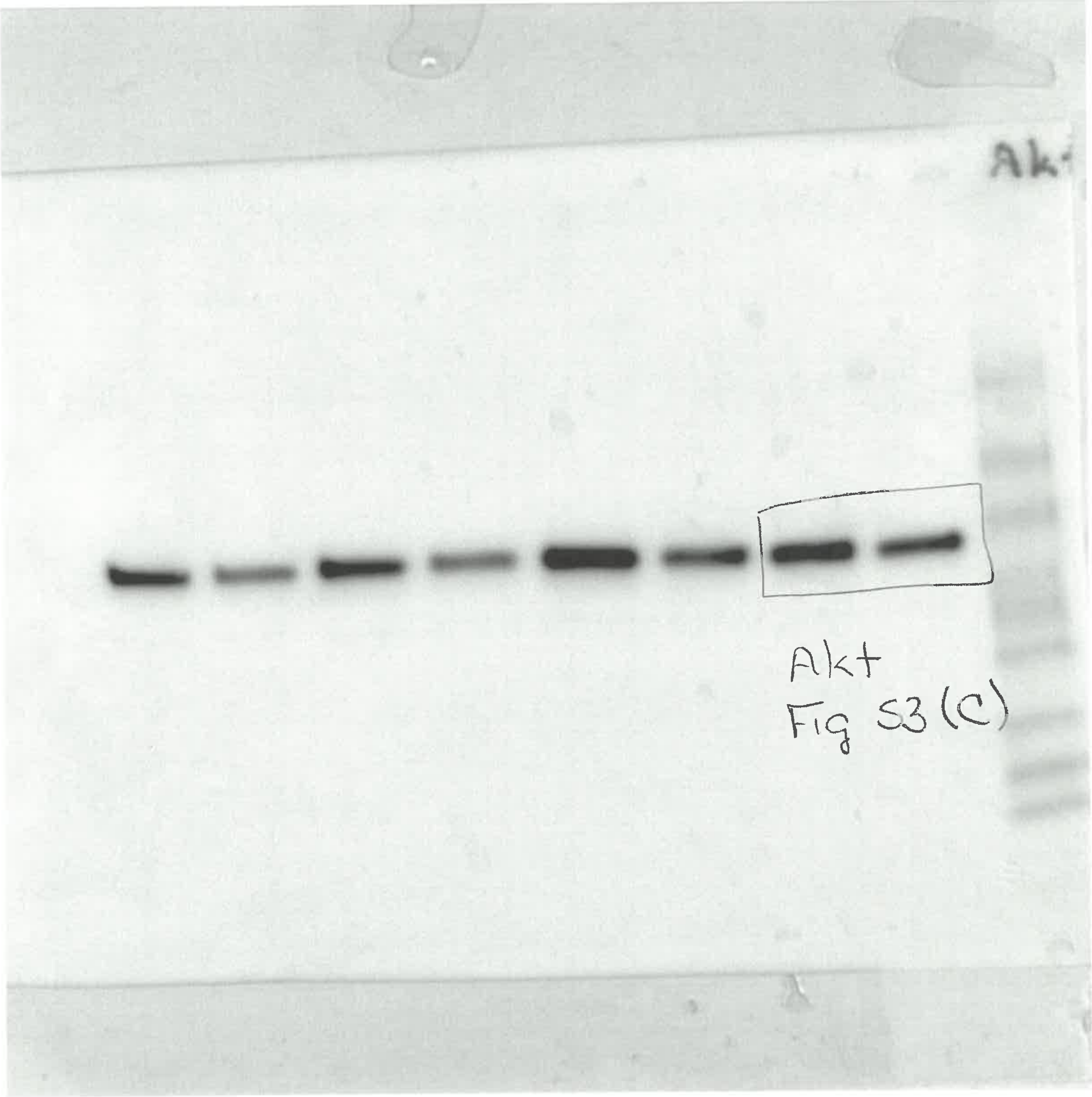
Ak
Akt
Fig S3 (c)

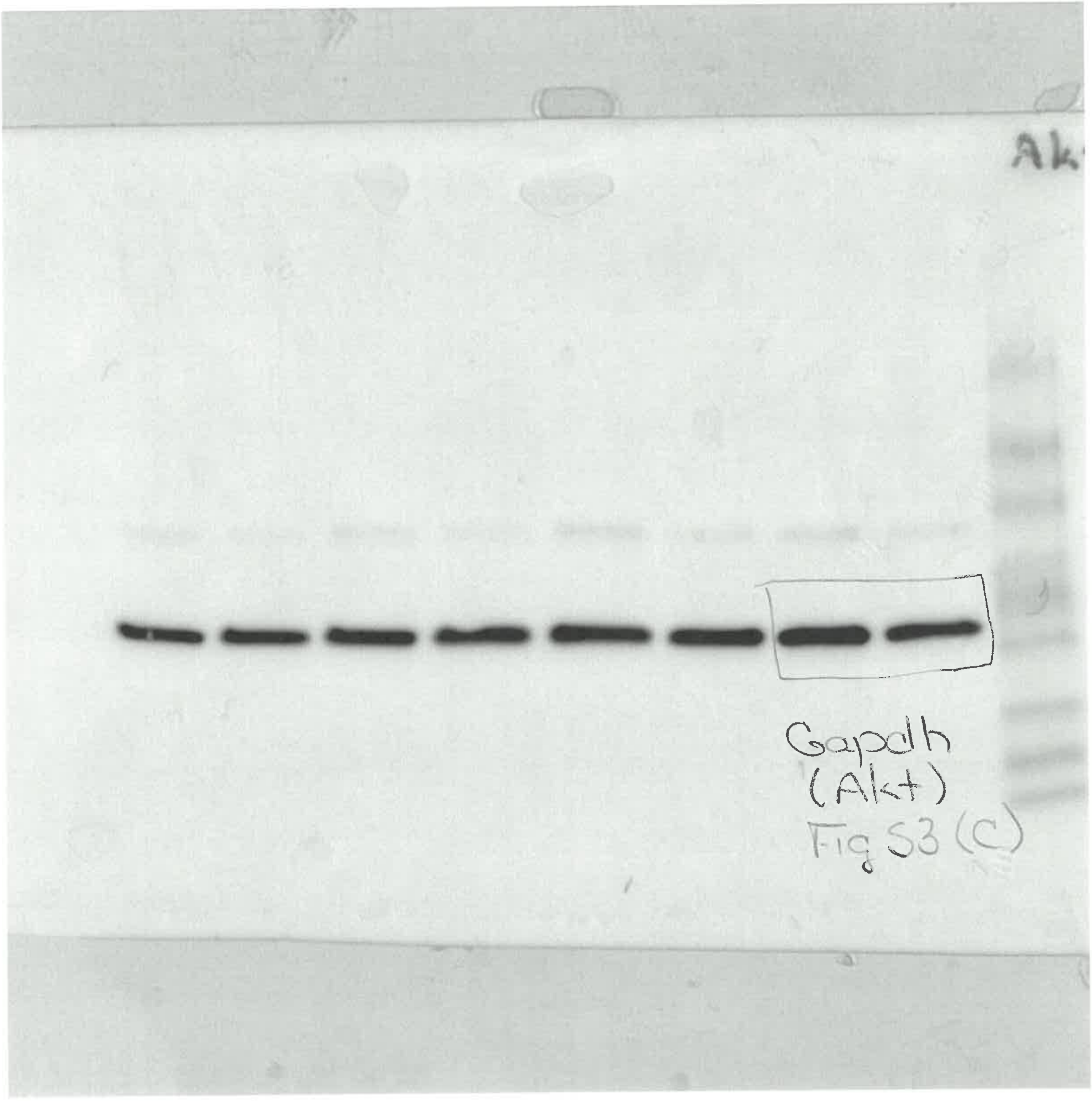
Ak
Gapdh
(Akt)
Fig S3 (C)

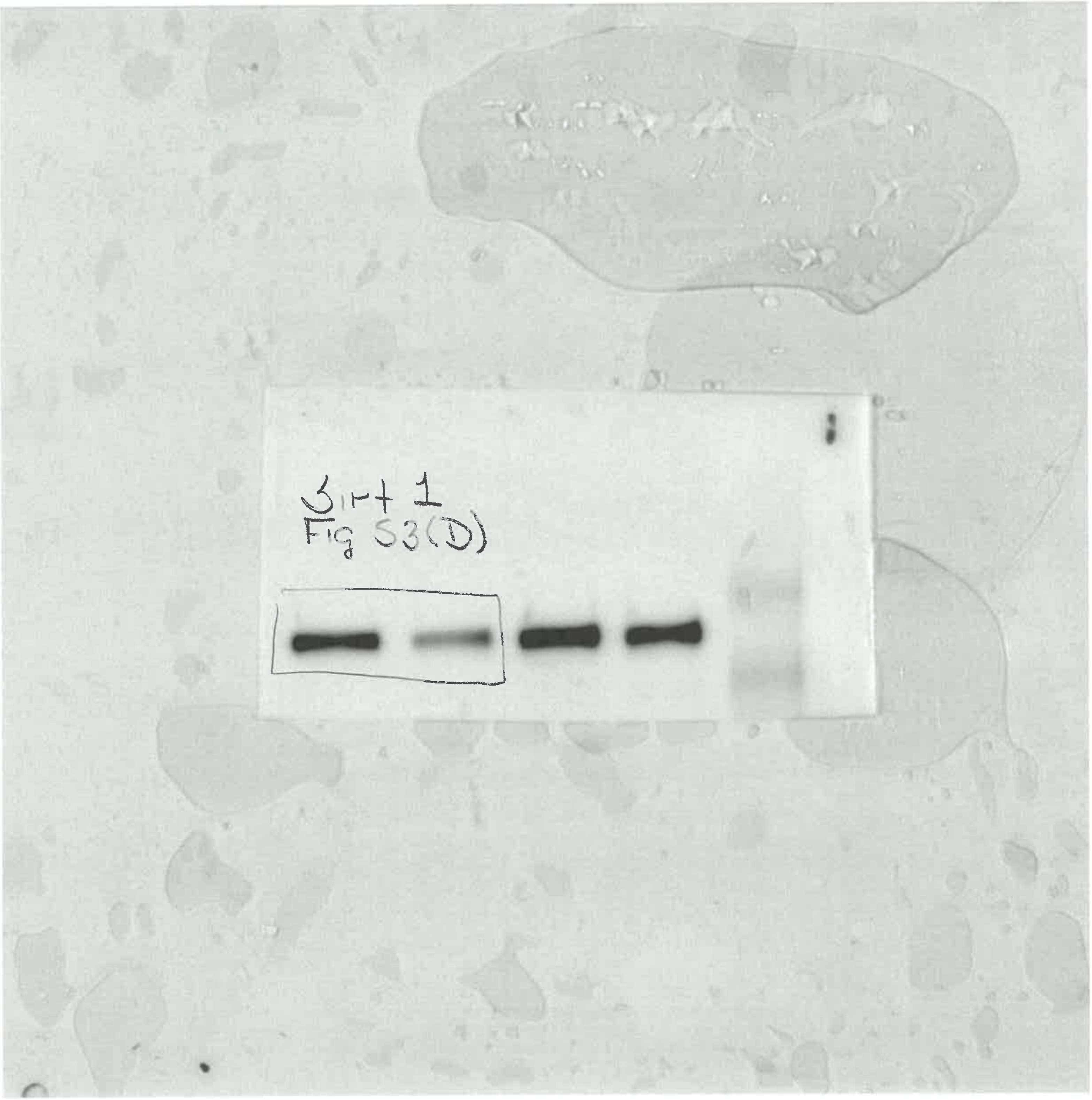
Sirt 1
Fig S3(D)

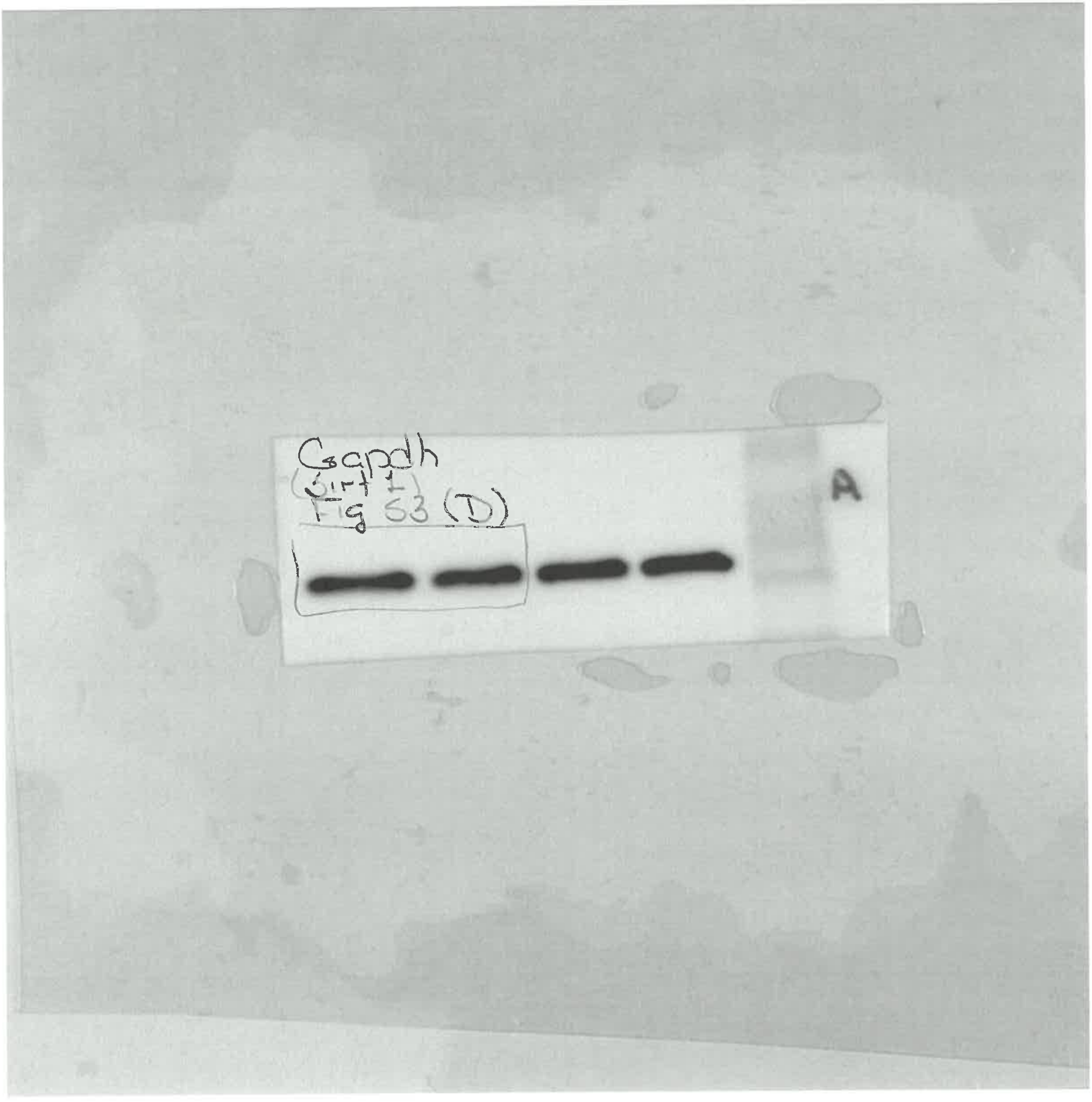
Gapdh
(Sirt 1)
Fig S3 (D)
A

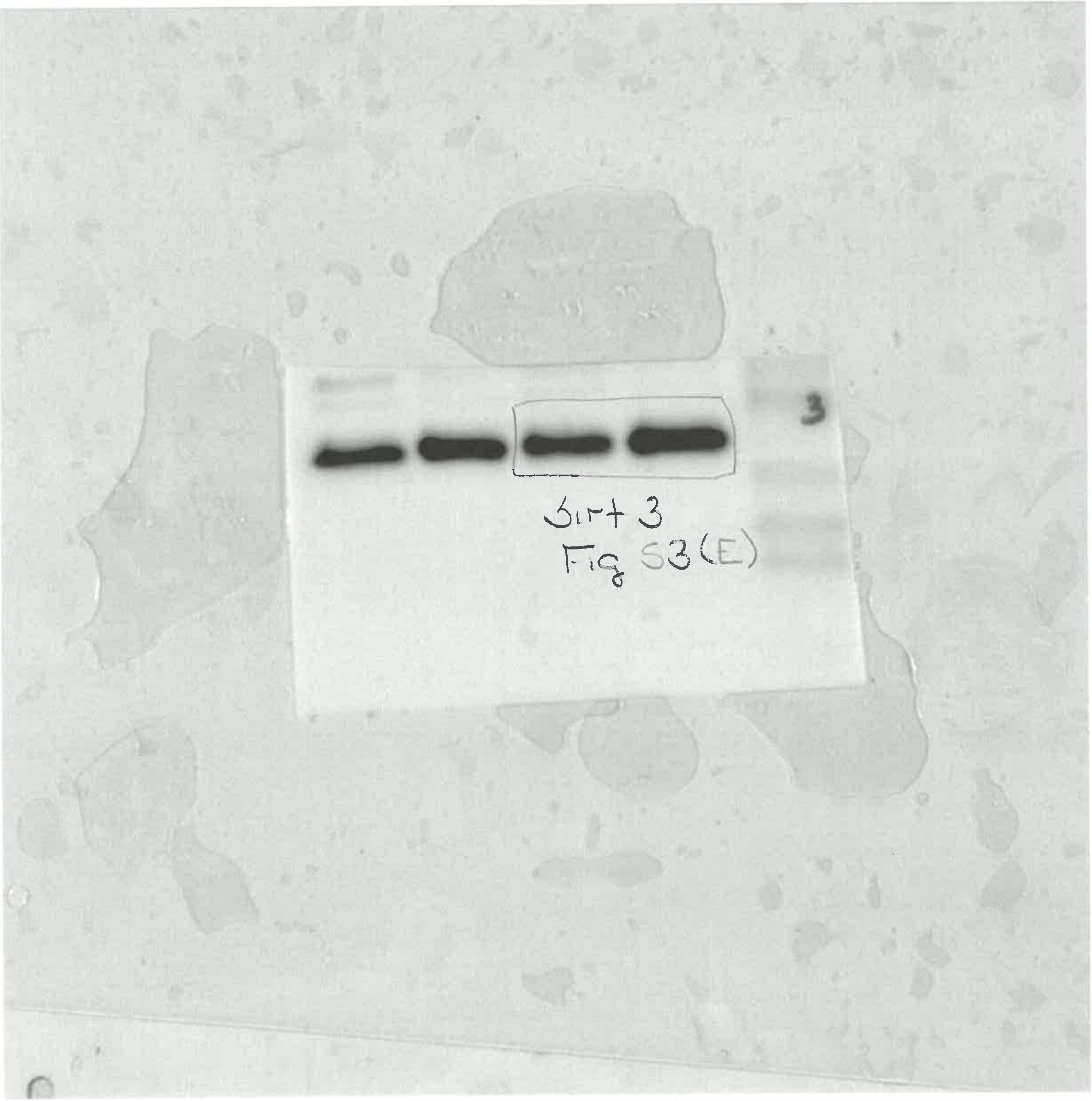
3
Sirt 3
Fig S3(E)

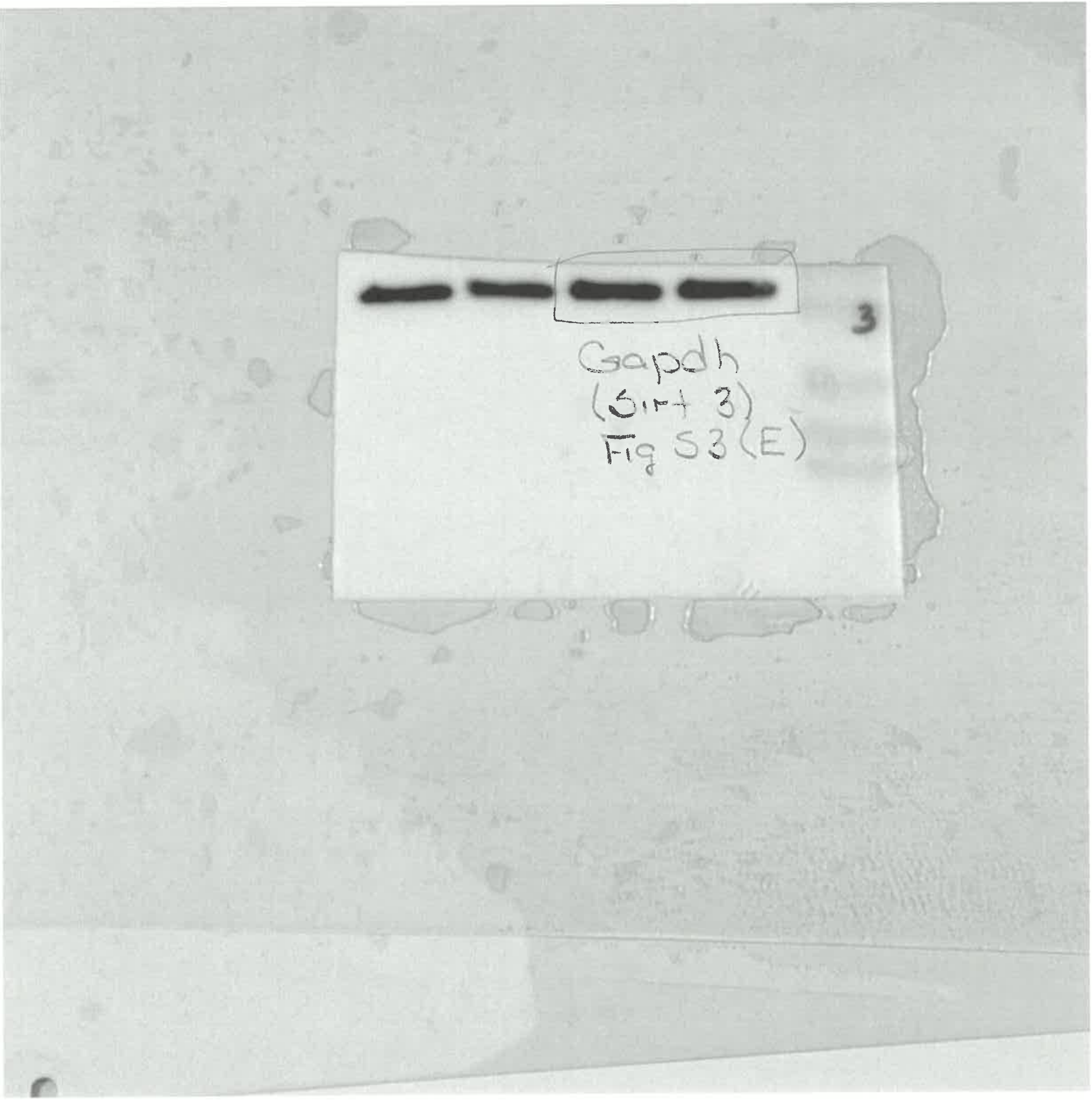
3
Gapdh
(Sirt 3)
Fig S3 (E)

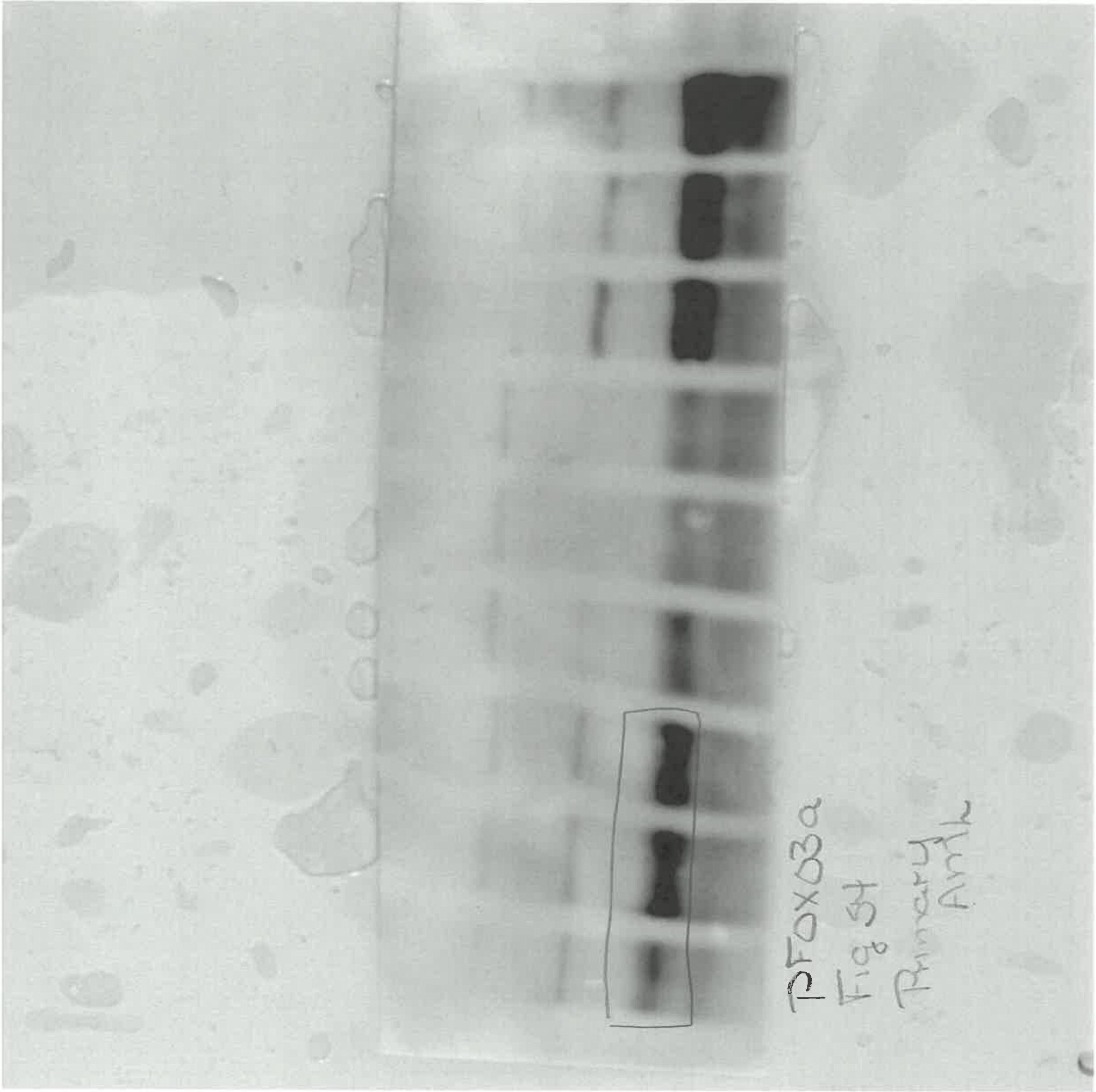
pFOXO3a
Fig 3I
Primary
Antib

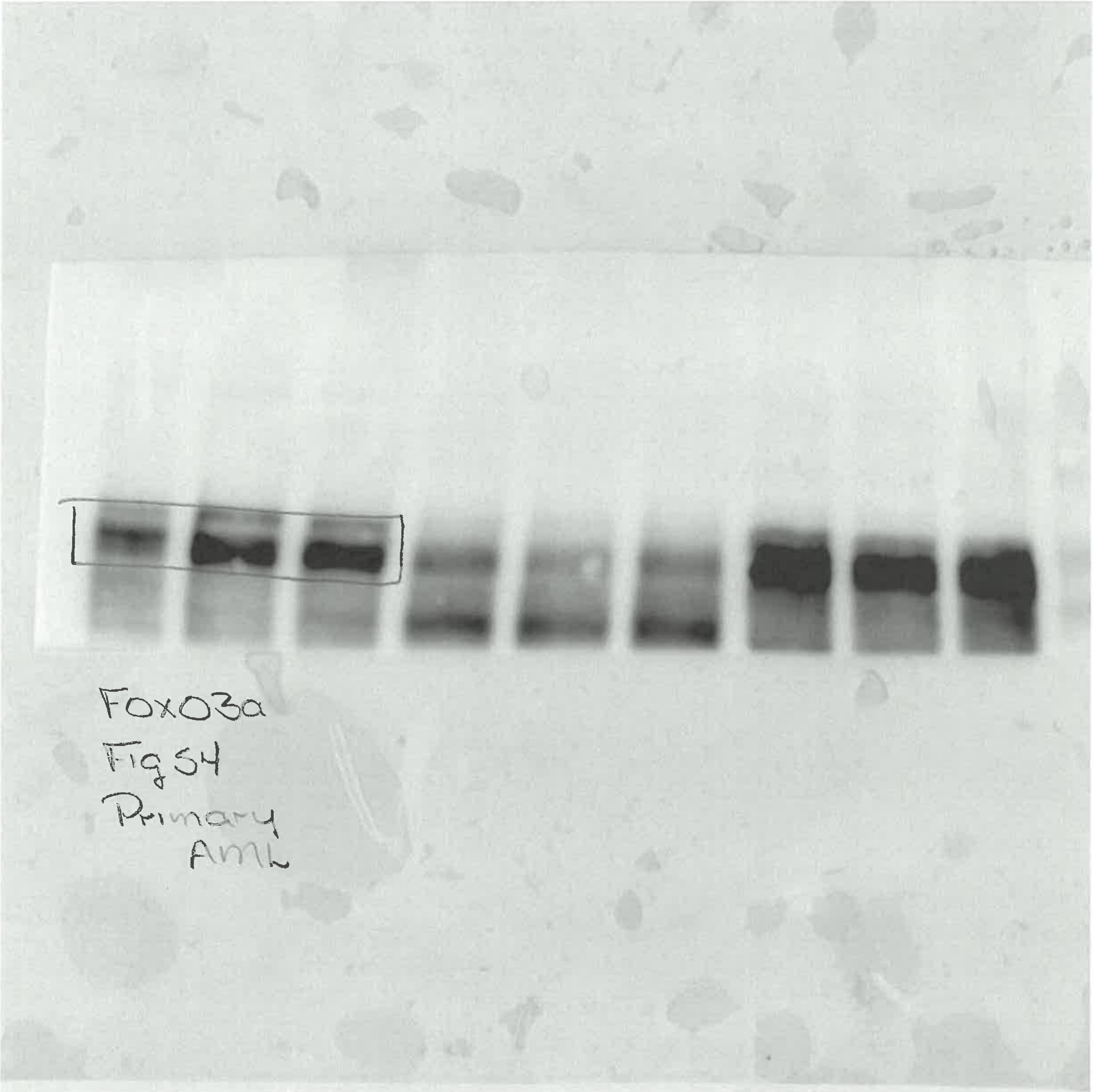
FoxO3a
Fig S4
Primary
AML

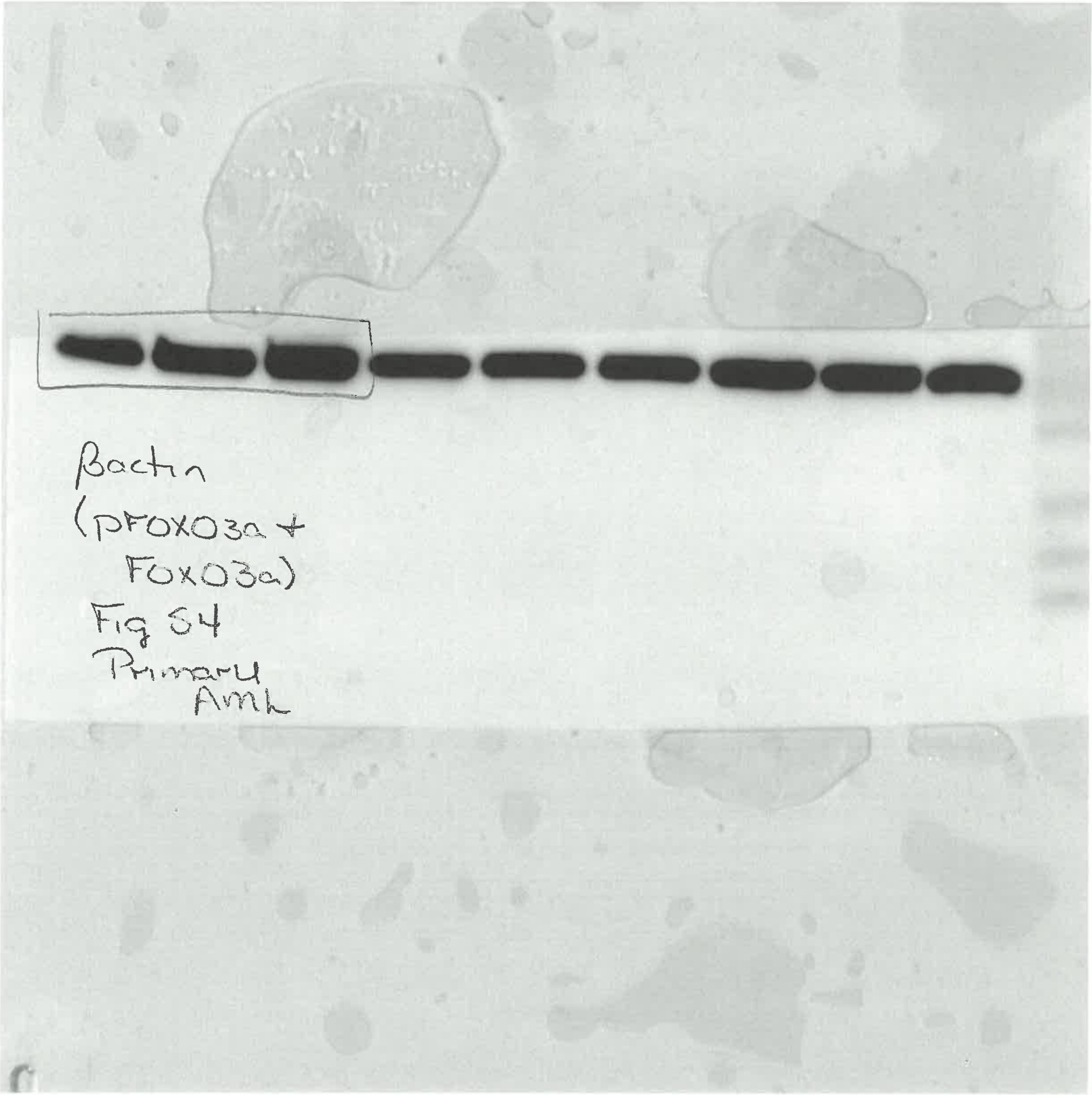
βactin
(pFOXO3a +
FOXO3a)
Fig S4
Primary
AML

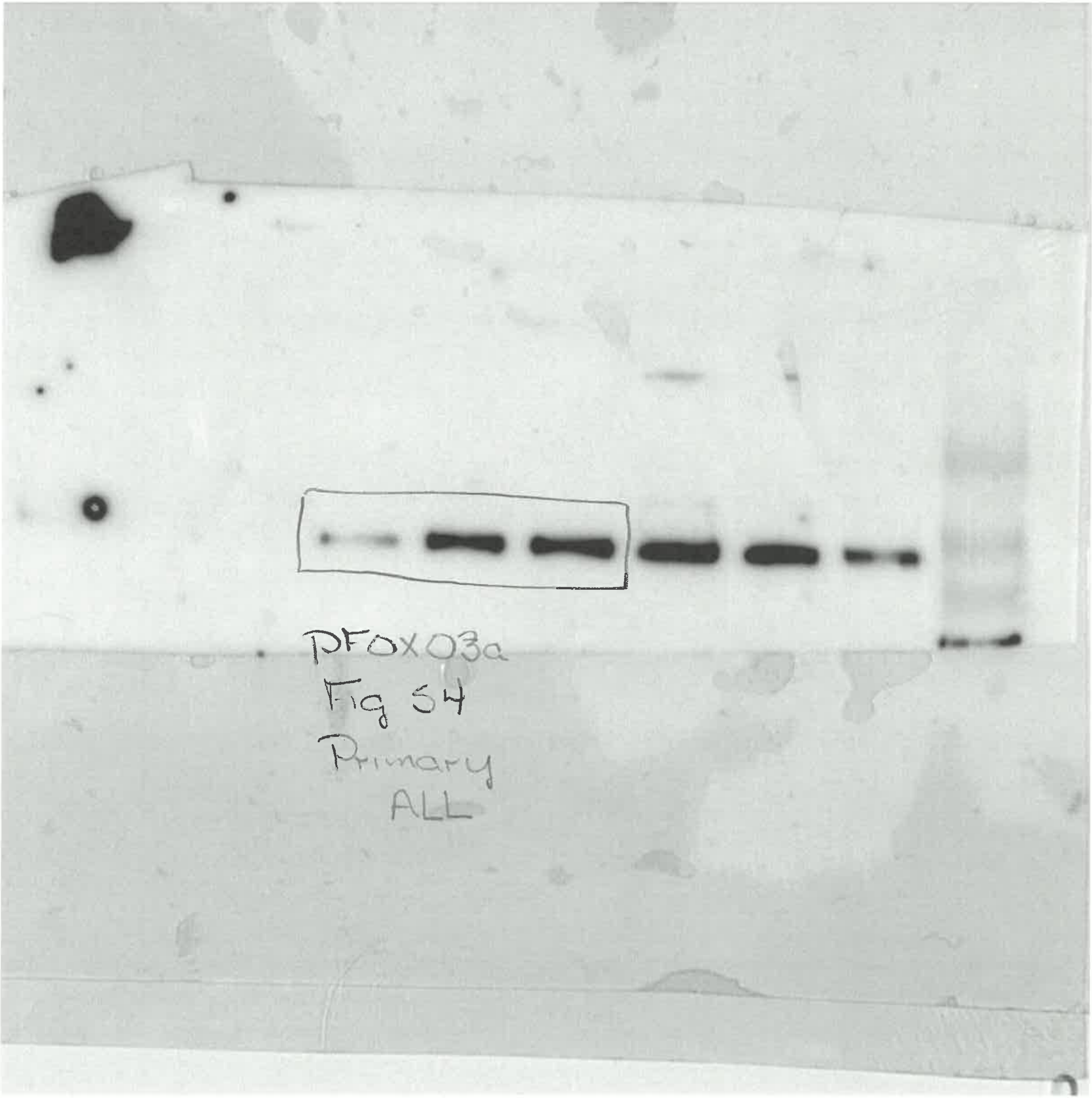
PFOXO3a
Fig 54
Primary
ALL

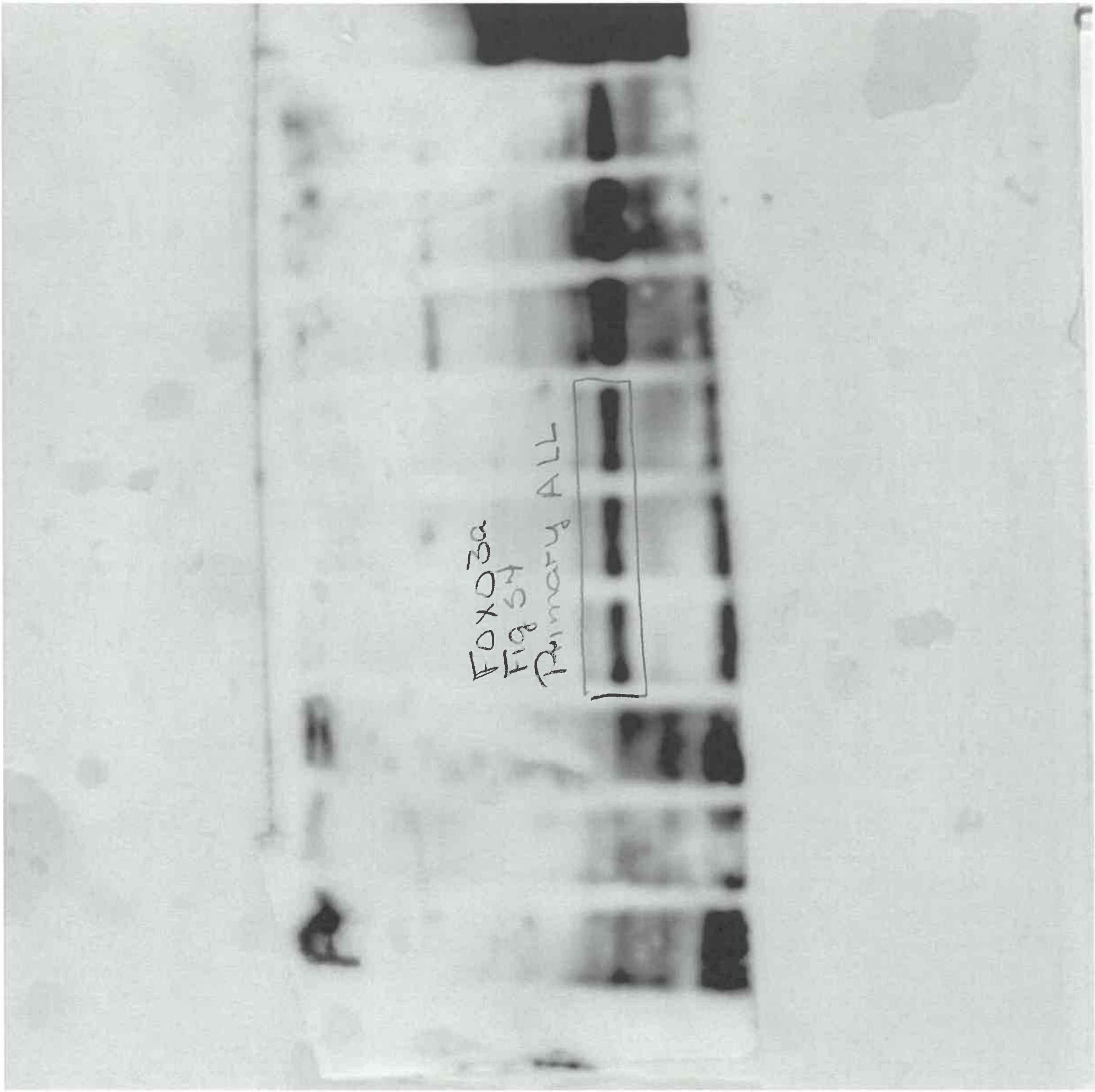
Foxo3a
Fig 54
Primary ALL

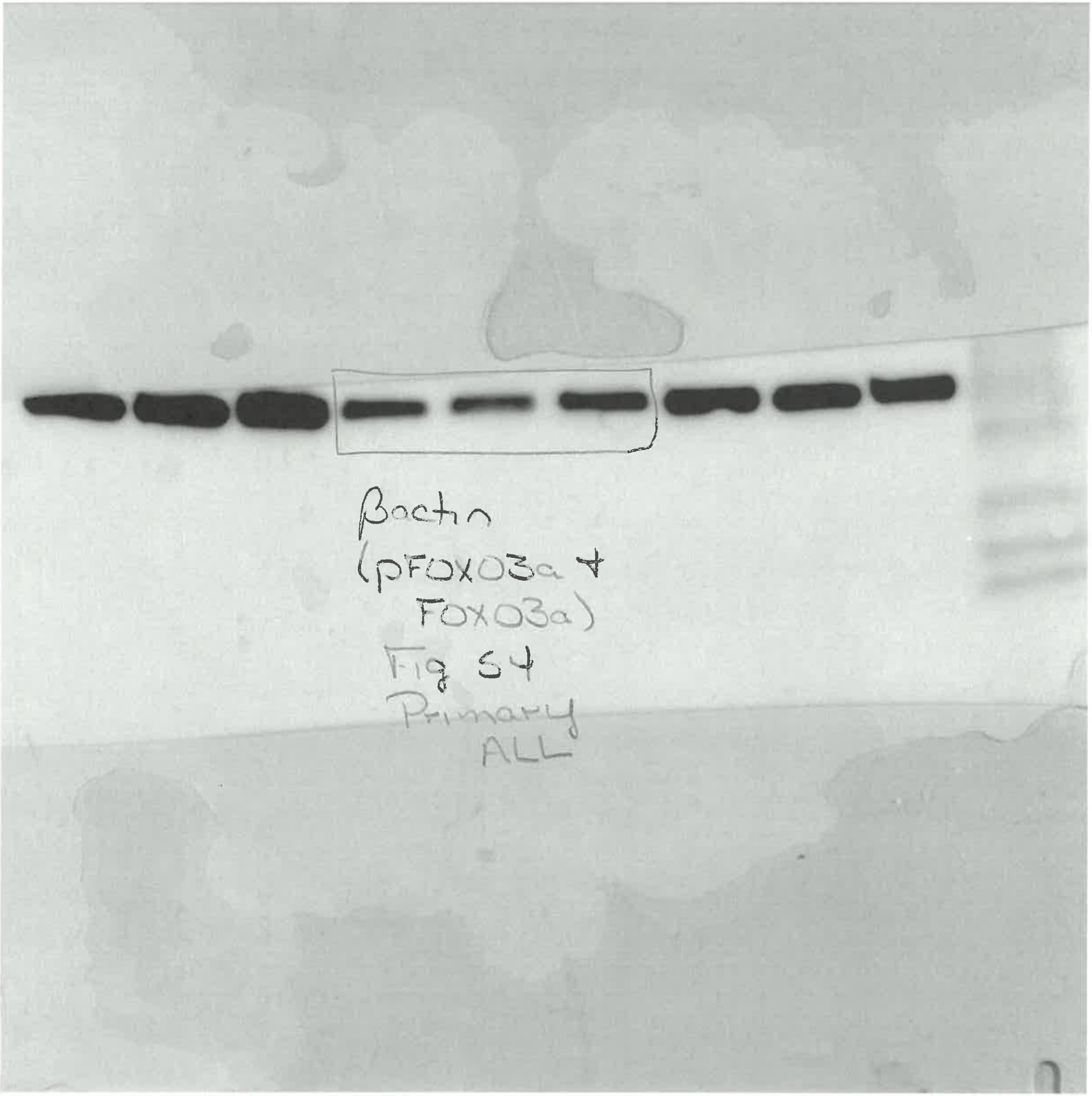
βactin
(pFOXO3a +
FOXO3a)
Fig S4
Primary
ALL
