# Supplementary material for: Chemotherapeutic Activity of Pitavastatin in Vincristine Resistant B-Cell Acute Lymphoblastic Leukemia
Source: Cancers (Basel). 2023 Jan 24;15(3):707. doi: 10.3390/cancers15030707 (PMC9913300; doi:10.3390/cancers15030707)
Supplement: Supplementary file 1 [file cancers-15-00707-s001.zip › cancers-2070903-supplementary.pdf]

Supplementary Materials

Chemotherapeutic Activity of Pitavastatin in Vincristine Resistant B-Cell Acute Lymphoblastic Leukemia

Debbie Piktel [1,2], Javohn C. Moore [1], Sloan Nesbit [1], Samuel A. Sprowls [3,4,5], Michael D. Craig [1,6], Stephanie L. Rellick [1,2], Rajesh R. Nair [1], Ethan Meadows [7,8], John M. Hollander [7,8], Werner J. Geldenhuys [3,8,9], Karen H. Martin [1,2] and Laura F. Gibson [1,2,*]

1 West Virginia University Cancer Institute, Robert C. Byrd Health Sciences Center, West Virginia University, Morgantown, WV 26506, USA
2 Department of Microbiology, Immunology and Cell Biology, West Virginia University School of Medicine, Morgantown, WV 26506, USA
3 Department of Pharmaceutical Sciences, West Virginia University School of Pharmacy, Morgantown, WV 26506, USA
4 Departments of Cardiovascular and Metabolic Sciences, Lerner Research Institute, Cleveland Clinic, Cleveland, OH 44195, USA
5 Case Comprehensive Cancer Center, Cleveland, OH 44195, USA
6 Queen's Health System, Honolulu, HI 96813, USA
7 Department of Physiology and Pharmacology, School of Medicine, West Virginia University, Morgantown, WV 26506, USA
8 Mitochondria, Metabolism & Bioenergetics Working Group, West Virginia University, Morgantown, WV 26506, USA
9 Department of Neuroscience, School of Medicine, West Virginia University, Morgantown, WV 26506, USA
* Correspondence: lgibson@hsc.wvu.edu; Tel.: +1-(304)-293-7206

Supplemental figures

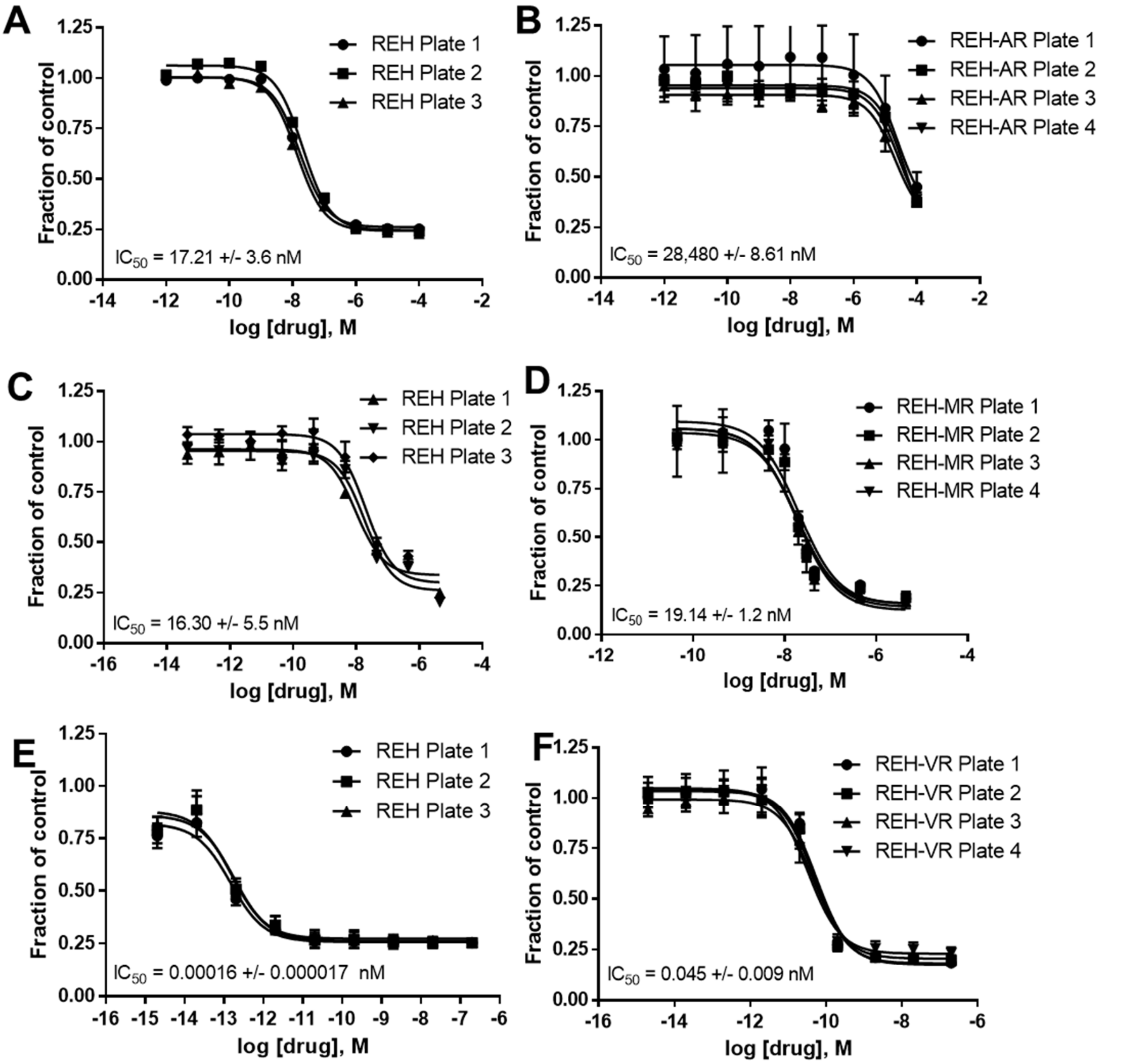

Figure S1. Dose-response effect of chemotherapeutics on cell proliferation, using CCK-8 (WST-8) reagent. Ara-C treatment in (A) REH and (B) REH-AR resistant cells; MTX treatment in C) REH and D) REH-MR resistant cells; and vincristine treatment in (E) REH and (F) REH-VR resistant cells. The IC_{50} for only Ara-C and vincristine was significantly different ($p < 0.05$; Student-t test), but not for MTX. Each data point is avg $\pm$ S.D., with N = 4. To show reproducibly of the assay, we repeated the experiments 3-4 times.

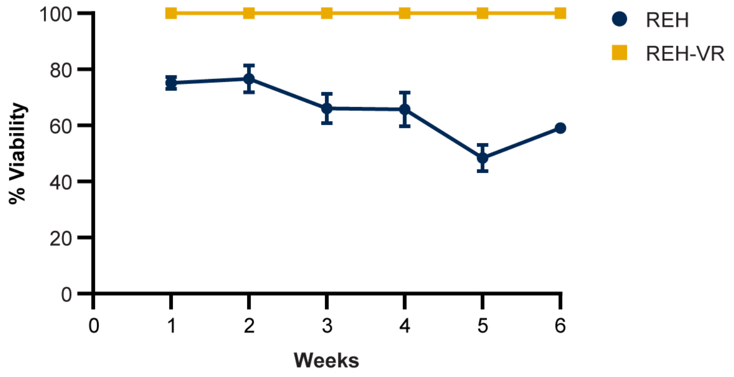

Figure S2. Stability of vincristine resistance. The vincristine resistance remained stable throughout the experimental design. Vincristine treatment was 2 nM. Percent viability determined using Trypan Blue exclusion method. N = 4. Data represents average $\pm$ S.D.

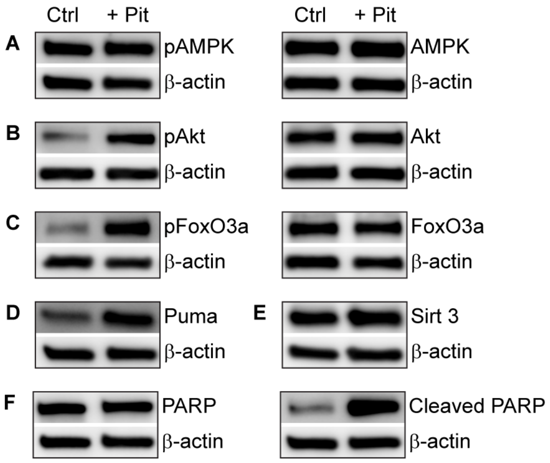

Figure S3. Pitavastatin effects on metabolic regulators. REH cells were treated with 450 nM pitavastatin (+ Pit) or DMSO vehicle control (Ctrl). Western blot analysis shows changes in phosphorylated and total protein levels following 48 hours treatment. We observed an increase in phospho-AKT, PARP cleavage, phospho-FoxO3a (Ser413), and Puma with pitavastatin treatment.

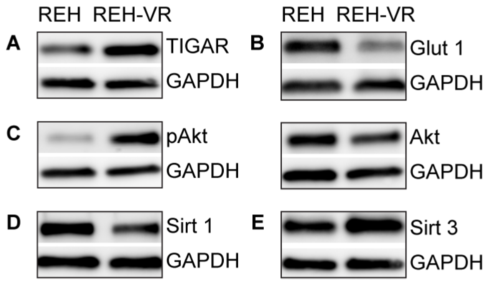

Figure S4. Differential expression of proteins in the vincristine-resistant cells. Western blot analysis of proteins in REH and REH-VR cells.

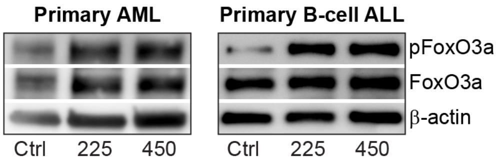

Figure S5. Pitavastatin treatment of primary leukemia samples. Primary human samples from AML and B-cell ALL patients were treated with pitavastatin at 225 nM and 450 nM and the protein expression levels of phospho-FoxO3a determined by Western Blot analysis**.** Control treatment (Ctrl) was DMSO vehicle**.**
